# Climate change and sexual and reproductive health and rights research in low-income and middle-income countries: a scoping review

**DOI:** 10.1136/bmjph-2024-001090

**Published:** 2024-08-24

**Authors:** Malachi Ochieng Arunda, Rachael Sorcher, Ana Paula Finatto Canabarro, Signe Svallfors, Margit Endler, Kristina Gemzell-Danielsson, Anna Kågesten, Moazzam Ali, Luis Bahamondes, María Barreix, Doris Chou, Lianne Gonsalves, Heidi Bart Johnston, James Kiarie, Caron Rahn Kim, Manjulaa Narasimhan, Christina Pallitto, Mehr Gul Shah, Lale Say, Anna Thorson, Anna Mia Ekström, Elin C Larsson, Vanessa Brizuela

**Affiliations:** 1 Global Public Health, Karolinska Institutet, Stockholm, Sweden; 2 Department of Sociology, Stanford University, Stanford, California, USA; 3 WHO Collaborating Centre for Research and Research Training in Human Reproduction, Department of Women’s and Children’s Health, Karolinska Institutet, Stockholm, Sweden; 4 Division of Gynecology and Reproductive Medicine, Karolinska Universitetssjukhuset, Karolinska University Hospital, Stockholm, Sweden; 5 UNDP/UNFPA/UNICEF/WHO/World Bank Special Programme of Research, Development and Research Training in Human Reproduction (HRP), Department of Sexual and Reproductive Health and Research, World Health Organization, Geneva, Switzerland; 6 Obst & Gynaecology, State University of Campinas Faculty of Medical Sciences, Campinas, Brazil; 7 Department of Infectious Diseases/Venhälsan, South General Hospital, Stockholm, Sweden

**Keywords:** Public Health, Social Medicine, Epidemiologic Study Characteristics

## Abstract

**Introduction:**

This study aimed to provide an overview of the research landscape and to identify research gaps linking climate change events and sexual and reproductive health and rights (SRHR) in low-income and middle-income countries (LMICs), where the negative impacts of climate change are most severe.

**Methods:**

We conducted a scoping review to map research studies that link climate change events or factors and SRHR aspects in LMICs. We performed a structured literature search across six databases to identify relevant peer-reviewed publications between January 1994 and 6 September 2023. The literature search yielded 14 674 peer-reviewed articles. After screening, 75 articles were included, spanning 99 countries across the globe.

**Results:**

Climate change events such as extreme temperatures, drought, rainfall shocks, cyclones and floods were found to be associated with negative maternal and newborn health outcomes ranging from reduced or low birth weight, preterm births and low Apgar scores, to lack of pregnancy care, pregnancy complications, stillbirths, and newborn and maternal deaths. Associations were also found between climate-related events and increased gender-based violence and HIV prevalence, as well as fertility decisions and harmful practices such as female genital mutilations and early and forced marriages. About two-thirds (48/75) of the articles were from the African or Western Pacific regions. The main research gaps on climate change-related events and SRHR included abortion, reproductive cancers and contraception use.

**Conclusion:**

Complementing existing evidence with targeted research to fill these knowledge gaps could enhance mitigation programmes and policies.

**WHAT IS ALREADY KNOWN ON THIS TOPIC**

- Earlier systematic, scoping and narrative reviews have examined the impact of climate change or specific climate change phenomena such as extreme heat on general or specific health aspects such as mental health, or HIV, and pregnancy outcomes including preterm birth, or on specific groups such as children. However, there is a notable absence of review studies that map the existing research body concerning the impact of climate change on broader sexual and reproductive health and rights (SRHR) in low-income and middle-income countries (LMICs).

**WHAT THIS STUDY ADDS**

- Our study contributes a distinctive and extensive overview of existing research on the interconnections between various climate change phenomena and all major SRHR domains, highlighting existing evidence and specific knowledge gaps to guide future research and mitigation efforts in LMICs, where populations in the most vulnerable situations to the effects of climate change live.

**HOW THIS STUDY MIGHT AFFECT RESEARCH, PRACTICE OR POLICY**

- The review reveals under-researched or unexplored areas to guide future scientific investigations on climate change phenomena and SRHR such as abortion, contraception and reproductive cancers. It also highlights how methodologies and research collaborations may be expanded moving forward to enable a more comprehensive understanding of SRHR and climate to guide future policies and intervention programmes addressing critical climate change-related threats.

## Introduction

 Long-term shifts in temperatures and changing weather patterns (ie, climate change) pose a major challenge to public health in the 21st century.^1^ Rising temperatures, rainfall shocks and an increase in the intensity and frequency of extreme weather events such as cyclones, directly and indirectly, threaten the health and well-being of populations worldwide, particularly those that already face risks and vulnerabilities in low-income settings.^2^ Climate change affects the entire planet, but its effects are often more dramatic around the equator, and the negative impacts are more severe in low-income and middle-income countries (LMICs) with less financial, infrastructural and geographical resources to mitigate the consequences.^3^ LMICs are also often heavily reliant on climate-sensitive sectors for income and survival, such as agriculture, fishing/aquaculture and tourism and may have less resources and adaptive capacity to address the impacts of extreme weather changes than high-income countries.^3^

Climate change may further aggravate pre-existing disparities in health, related to factors such as age, socioeconomic status, ethnicity, race, disability, indigeneity, as well as sex and gender differences.^2^ The impacts of climate change on sexual and reproductive health and rights (SRHR) may be exacerbated due to gender inequalities.^4^ As a result, women and children, particularly girls, as well as other vulnerable populations, face additional challenges when it comes to mitigating climate-related poor health consequences.^4^,^6^ For instance, drought conditions can cause food insecurity which disproportionately impacts women and children.^6 7^ Situations of food insecurity can exacerbate existing nutritional vulnerabilities (eg, iron deficiency) among pregnant or breastfeeding women.^5^ Studies also show that climate change-induced floods contribute to population displacement and disrupt the provision of healthcare services, including life-saving sexual and reproductive health services such as facility-based childbirth, HIV prevention and contraception.^5 8^ Furthermore, experimental studies and reviews reveal that extreme temperatures, floods and droughts directly impact SRHR through several mechanisms. For instance, Hnat *et al* found that among pregnant women, extreme temperatures can lead to increased sweating due to thermoregulation, which can cause dehydration and trigger early labour.^9^ Heat stress also causes a rise in cortisol levels, potentially leading to decreased blood flow to the placenta as blood is diverted for other immediate bodily needs. This can consequently affect fetal growth due to reduced oxygen levels in the fetus.^10^

While the evidence base for gender, health and climate change interlinkages is growing, considerable research gaps remain at the intersection of climate change and SRHR which to date has received inadequate attention.^2^ Previous systematic, scoping and narrative reviews of climate change in LMICs have focused on overall health, specific health areas such as child health, mental health, chronic illnesses and nutritional health or health systems of specific populations (eg, children).^16^,^8^ Yet, to the best of our knowledge, none has documented existing published literature on SRHR and climate change in LMICs. In order to bridge this gap, the UNDP/UNFPA/UNICEF/WHO/World Bank Special Programme of Research, Development and Research Training in Human Reproduction (HRP), housed at the WHO, through its HRP Alliance for research capacity strengthening convened a group of experts to scope existing evidence at this intersection.^11^ The goal is to provide an exploratory mapping of research that links climate change factors or events and SRHR aspects in LMICs and to identify existing research gaps. By highlighting research gaps particular to sexual and reproductive health, we aim to advance evidence-based interventions, mitigation, adaptation and policy improvements. This review charts existing research and thereby can guide the direction of future research in this space.

## Methods

We conducted a scoping review in accordance with the Preferred Reporting Items for Systematic Reviews and Meta-Analysis—scoping reviews extension (PRISMA-ScR, https://www.prisma-statement.org/scoping—see online supplemental file 1, pages 25–27). The protocol was registered in the Open Science Framework available at https://osf.io/a4wm5/

### Search strategy

A structured literature search was conducted in the following databases: Medline, Embase, Web of Science Core Collection, CINAHL, Google Scholar and World Health Organization (WHO) Global Index Medicus in December 2022 and repeated on 6 September 2023, to identify relevant peer-reviewed publications between January 1994 and 6 September 2023. The search strategy was developed on Medline (Ovid) in collaboration with librarians at the Karolinska Institutet University Library. For each search concept, relevant Medical Subject Headings (MeSH terms) and free-text terms were identified. The search was then translated, in part using Polyglot Search Translator,^12^ into the other databases.

The search strategy focused on identifying articles that explored the intersections of climate change and SRHR. Distinct keywords and MeSH terms specific to SRHR (eg, “maternal health” or “abortion”) and climate change (eg, “drought” or “floods”) were cross-combined and searched with search terms specific to LMICs (eg, “Philippines” or “Sierra Leone”). The detailed search strategy, including how deduplication and additional steps for comparing DOIs were done, can be found in online supplemental file 1 (pages 27–50).

We adopted the 2018 definition of SRHR by the Guttmacher-Lancet Commission as ‘a state of physical, emotional, mental and social well-being in relation to all aspects of sexuality and reproduction, not merely the absence of disease, dysfunction or infirmity’, relying on the ‘realisation of sexual and reproductive rights’ that builds on globally established human rights conventions such as the Universal Declaration of Human Rights, the right to health, the rights of the child, the Convention on the Elimination of All Forms of Discrimination against Women and the Sustainable Development Goals (SDGs).^13^ In this review, our understanding of climate change events, such as heat waves, droughts and floods, is based on the Intergovernmental Panel on Climate Change (IPCC) 2022 report.^14^

### Selection criteria

Peer-reviewed articles in any language were considered for inclusion if they empirically measured or explicitly analysed the intersection of climate change and SRHR. We limited the search to publications from January 1994 to reflect when the United Nations Framework Convention on Climate Change was ratified, and when the United Nations International Conference on Population and Development in Cairo adopted a human rights approach to sexual and reproductive health. The final database search and inclusion date was 6 September 2023.

We included only original research articles based on quantitative (eg, cohort-based, cross-sectional and time-series), qualitative and mixed-methods study designs. We included all articles that explored SRHR and climate change in LMICs affecting all populations regardless of age, sex or gender. Since there is no standardised classification of SRHR factors with respect to climate change, we adapted the Guttmacher-Lancet Commission framework together with specific SDG targets focusing on broad sexual and reproductive health domains and related essential services.^13^ Based on this framework, we divided included articles into the following categories: maternal and newborn health, abortion, HIV and other sexually transmitted infections (STIs), contraception, fertility care (including pregnancy intentions and timing, family size, and infertility), reproductive cancers, gender-based violence (GBV), harmful practices (including early/forced marriage and female genital mutilation (FGM), and multiple (a few studies researched several SRHR aspects including maternal and newborn health, GBV, harmful practices and fertility care).

Similarly, due to the absence of a standardised classification for climate change phenomena, we categorised climate change events based on those identified as detrimental to health and well-being by the IPCC.^14^ Articles were included if they (1) explicitly measured how climatic/weather events changed over time by increasing in frequency or intensity or duration in the study context and/or (2) used reference measures to denote how those events were ‘extreme’ (eg, temperature ≥95th percentile or ≤5th percentile) and/or (3) defined that the phenomena analysed were anomalies. If articles did not use the term ‘climate change’ but used the above description, they were still included. Thus, the following major climate change events were considered: extreme temperature (including the subcategories of increasing/extreme heat and decreasing temperature/cold spells), rainfall shocks (including positive and negative shocks), drought, floods, cyclone/typhoons and multiple climate change events (a few studies researched several of the included events such as drought, abnormal temperatures and precipitation, floods and cyclones).^14^

We excluded articles focusing on predictions or projections of future scenarios. Systematic reviews, scoping and literature reviews, meta-analyses, conceptual frameworks, book reviews, news reports, editorials, letters, commentaries, viewpoints, theoretical articles, mathematical models and non-peer-reviewed reports were excluded. Articles that did not report their methodology or focused on populations living in high-income countries were excluded. Articles on child health beyond the perinatal period were also excluded, except if the article(s) related to SRHR outcomes such as in early/forced marriage FGM or GBV. We excluded studies on natural disasters unrelated to anthropogenic climate change (eg, earthquakes and tsunamis), dust storms and air pollution (including those related to volcanic eruptions and wildfires) since their connection to climate change is complex and less direct. Specific to air pollution, the complexity can be seen in how several reviews have examined the impact of air pollution on SRHR including in LMICs without mentioning climate change,^15^ while a most recent review only examined the impacts of air pollution and climate change separately on SRHR.^16^ Further, studies that focused on interventions related to climate change adaptation, mitigation, resilience and coping were excluded unless they were directly linked to SRHR outcomes.

### Study selection

The search results were first imported into an EndNote Library and then uploaded to Rayyan, a web-based software platform for conducting reviews (available from: https://www.rayyan.ai) from where identified duplicates were removed. Three authors (MOA, RS and APFC) screened the articles by titles and abstracts for inclusion and removed any remaining duplicates. This was followed by full-text extraction of relevant articles by two reviewers (RS and APFC) to create a literature database; a third reviewer (MOA) verified all final inclusion decisions and was the tiebreaker in cases of conflict.

### Data abstraction and charting

For all included articles, we extracted information regarding title, year of publication, study country and income level following the World Bank classification, study setting (urban, rural, mixed), study design (quantitative, qualitative or both), sample and data source, SRHR domain, climate change event, climate data source and key findings. Additionally, we categorised all study countries by WHO regional classification.^17^ We also reported the first and corresponding authors’ affiliated institution, country and income classification, and affiliations of any other author. Complete details are presented in online supplemental file 1.

## Results

**Figure 1:**
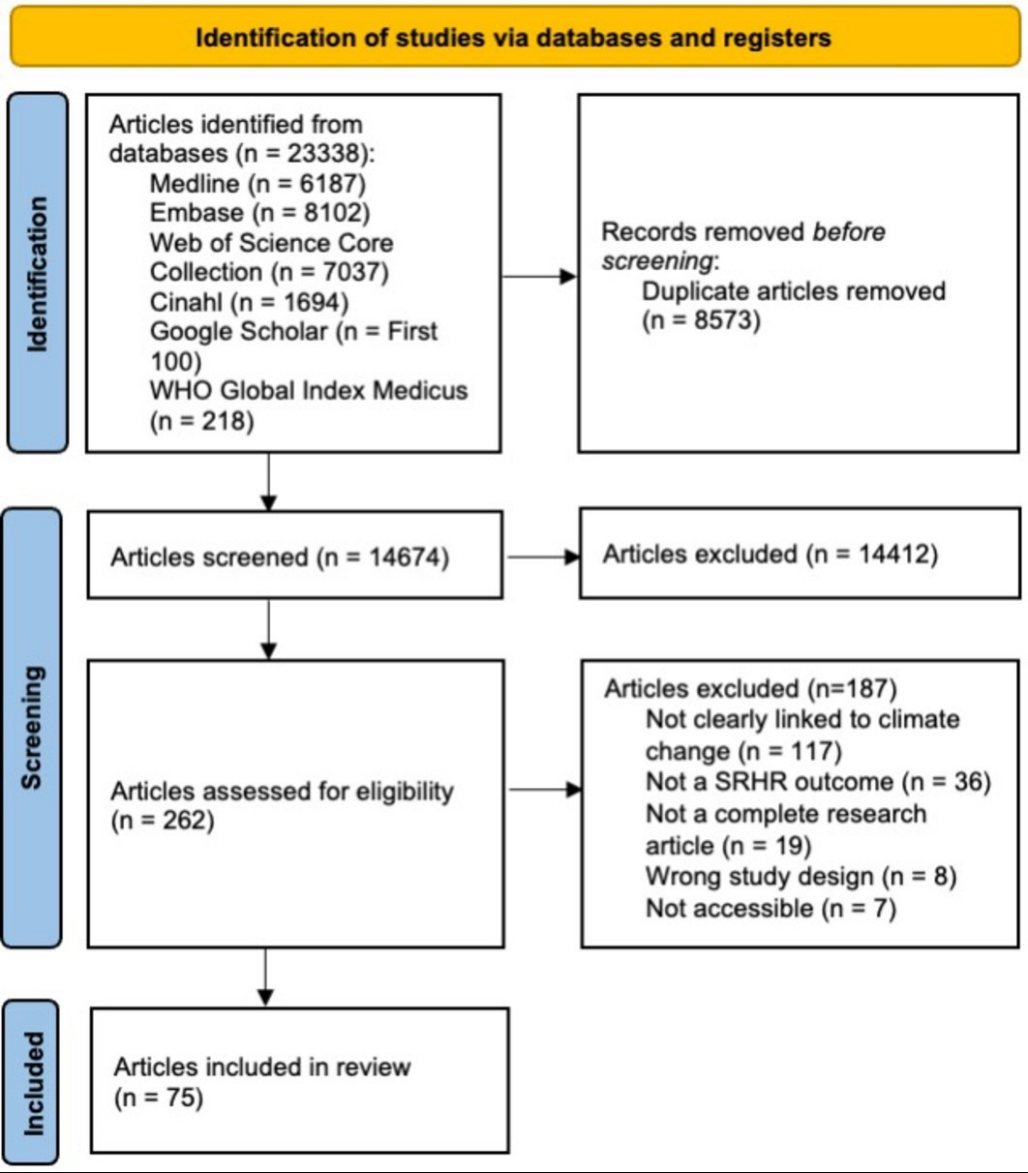
PRISMA diagram showing the study selection process of articles. PRISMA, Preferred Reporting Items for Systematic Reviews and Meta-Analyses; SRHR, sexual and reproductive health and rights.

We identified a total of 23 338 records and after removal of 8573 duplicates, 14 674 were screened for eligibility by title and abstract. 262 records were retrieved for full text review and 75 articles were finally included in this scoping review. Figure 1 displays the PRISMA flow diagram showing a summary of the article selection process for the scoping review.

### Characteristics of included articles

**Figure 2:**
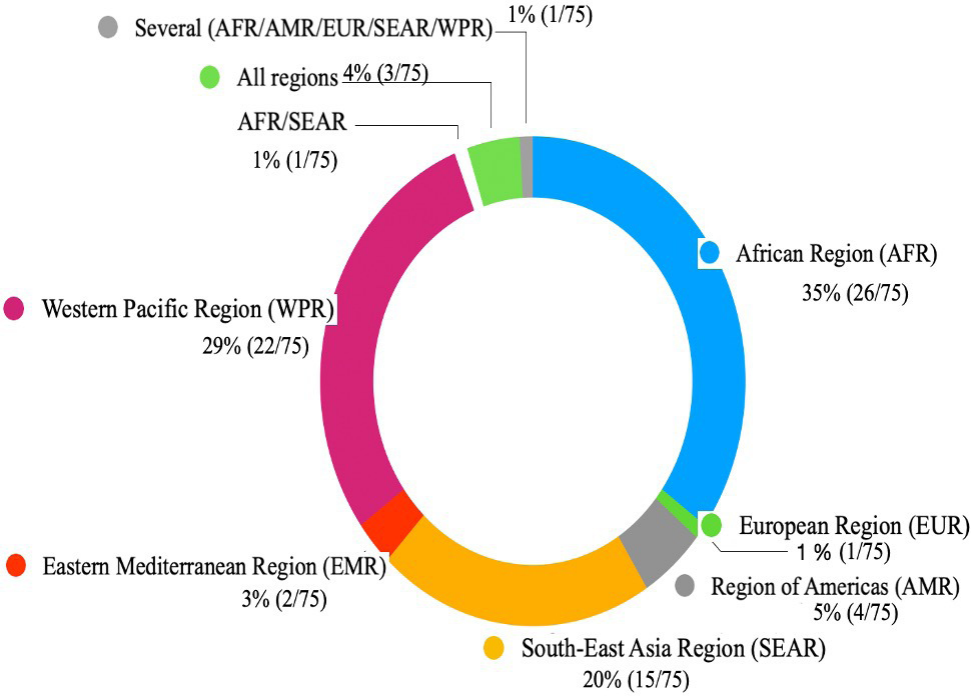
Distribution of 75 articles on climate change and sexual and reproductive health and rights, published 1994–2023 in low-income and middle-income countries by WHO regions.

Table 1 and online supplemental file 2 present an overview of the characteristics of the 75 articles included.^18^,^92^ The search yielded articles across 99 LMICs, with over 80% published between 2018 and 2023. Figure 2 shows that the majority of the articles focused exclusively on African (26), Western Pacific (22) and South-East Asian regions (15) constituting 84% of the total articles. A detailed synthesis of the 75 studies and a list of study countries can be found in online supplemental file 1 (tables A (p 27–50) and B (p 51–52), respectively). 47 (63%) articles had at least one author affiliated with institutions in LMICs, the majority of whom were from the countries where the studies were conducted.

**Table 1:** Key climate change events and sexual and reproductive health and rights characteristics studied by 75 articles, published 1994–2023

Characteristics		No. of articles (%)	References (n=75)
Study design*	Quantitative	57 (76.0)	19 2429 30 33 35
	Qualitative	13 (17.3)	2526 31 34 39 52 59 60 70 73 87 89
	Mixed quantitative and qualitative	05 (6.7)	18 27 28 32 82
Climate change event/factor(s)†	Drought	18 (~24.0)	20 2224 25 30 31 35 71
	Extreme temperatures	33 (~44.0)	2933 36 37 40 45 48
	Floods	10 (~13.3)	19 27 34 38 52 60 70 82 83 87
	Rainfall shocks	14 (~18.7)	2333 37 46 47 58 73 77 79 81 85 90 92
	Cyclones/typhoons	04 (~5.3)	26 27 59 83
	Multiple (3+) climate factors‡	06 (8.0)	18 28 32 39 84 89
Sexual and reproductive health and rights dimension studied	Maternal and newborn health	37 (49.3)	34 6992
	Gender-based violence	09 (12.0)	18 26
	HIV and other STIS	09 (12.0)	70 78
	Fertility care§	07 (9.3)	79 85
	Harmful practices¶	07 (9.3)	27 33
	Contraception	01 (1.3)	91
	Multiple SRHR domains**	05 (6.7)	86 90

* Quantitative (employing surveys, eg, Ddemographic and health surveys, Mmultiple Iindicator Ccluster Ssurveys, birth registries, etc. linked to meteorological or hydrological data); qualitative (employing key informant interviews, in-depth interviews, and focus group discussions in climate change affected/prone areas).

† Climate change figures exceeds totals due to some articles examining two or more climate change factorsevents/factors.

‡ Three or more climate change events (include drought, extreme temperatures, floods, cyclones).

§ Fertility care (includes fertility and infertility).

¶ Harmful practices (includes female genital mutilation and forced/child marriage).

** Multiple SRHR domains (includes maternal and newborn health, gender-based violence, harmful practices, fertility care).

SRHRsexual and reproductive health and rightsSTIssexually transmitted infections

### Populations studied, sample size and study design

**Figure 3:**
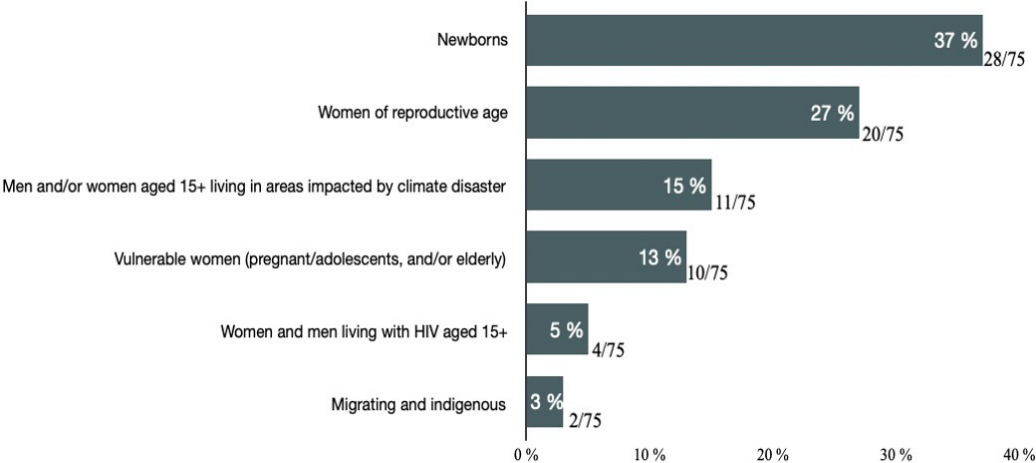
Populations studied by 75 articles on climate change and sexual and reproductive health and rights, published 1994–2023 in low-income and middle-income countries.

Figure 3 displays the distribution of populations studied by a number of articles, with newborns and women of reproductive age accounting for 28 and 20 articles, respectively, constituting more than half (55%) of all the articles. Four (5%) articles focused on men and women living with HIV, aged 15+ years. Quantitative studies constituted three-quarters (57 out of 75) of the articles, with the sample sizes ranging from 105 to 5.44 million and were mainly cross-sectional and retrospective designs. 13 (17%) articles employed qualitative approaches, mainly key informant interviews and focus group discussion, and five (7%) were mixed-methods studies.

### Climate change exposures

The impact of extreme temperatures on SRHR was the most extensively studied area including 33 articles (44.0%) encompassing factors such as heat waves, cold spells and increasing ambient temperatures. This was followed by articles on drought (18), rainfall shocks (14), floods (10) and cyclones/typhoons (4), with a few articles examining more than 2 climate change events. Six articles examining the impact of three or more climate change events on SRHR.

### SRHR outcomes and key findings

**Figure 4:**
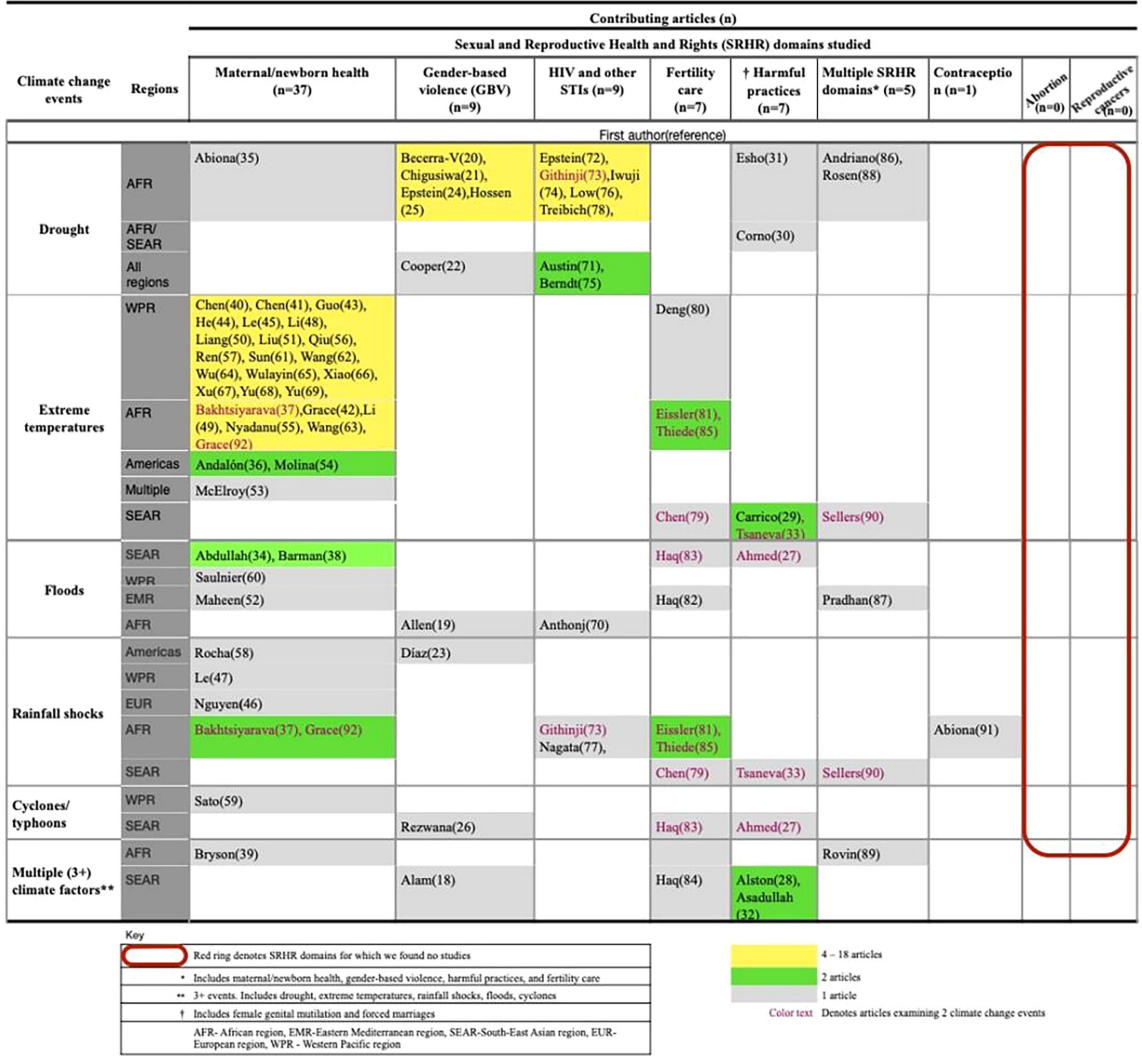
A matrix of climate change events and SRHR categories, studied by 75 articles, published between 1994 and September 2023 in LMICs. LMICs, low-income and middle-income countries.

Figure 4 is a matrix showing SRHR domains studied with respect to specific climate change events.

#### Maternal and newborn health

This was the most studied SRHR domain with 37 (49%) out of 75 articles across various climate change events. Among these, 73% (27/37) focused on the impact of extreme temperatures^3637 40^,^45 48^ on maternal and newborn health, with 21 out of the 27 from the Western Pacific region, primarily China. There were no articles on extreme temperatures from South-East Asian, European and Eastern Mediterranean regions in this SRHR domain. Other studies of climate change factors with respect to maternal/newborn health included drought,^35^ rainfall shocks,^37 46 47 58 92^ floods^34 38 52 60^ and cyclones/typhoons.^59^ One study looked at multiple climatic events.^39^ Generally, the studies reported associations between climate change events such as extreme temperatures (hot or cold), prolonged drought and neonatal outcomes such as reduced or low birth weight,^35^,^3741 42 45^ increased preterm birth,^43 44 50 53 56 57 62 64 66 68^ low Apgar scores,^36 40^ shorter length at birth,^36^ congenital heart defects,^67 69^ macrosomia^49^ and stillbirth.^52 55^ Studies also found associations between flooding, cyclones and typhoons with lack of appropriate pregnancy care, pregnancy complications and maternal death.^34 38 52 59 60^ Further, rainfall shocks were associated with shorter gestational periods, infant mortality^58^ and large birth weights.^37 92^

#### Gender-based violence

Nine articles studied GBV or violence against women and girls in relation to climate change events. Five out of the nine articles were exclusively from the African region. Climate change phenomena researched in this domain included drought,^20^,^2224 25^ floods,^19^ cyclones and typhoons,^26^ rainfall shocks^23^ and multiple climate change events including extreme temperatures.^18^ Studies reported associations between floods, drought, cyclones and GBV including sexual violence,^19 24 26^ psychological^26^ and physical violence.^19 23 24 26^ However, no study reported on sex trafficking or sex work in the context of the climate crisis.

#### HIV and other STIs

Nine articles studied climate change and HIV, seven of which were exclusively from the African region. Drought was the predominant climate factor examined with respect to HIV. Others included floods and rainfall shocks. The studies reported associations between extreme climatic events particularly droughts, floods, rainfall shocks and increased HIV prevalence.^7073 75^,^78^ Studies also found these climate-related events and HIV risk factors such as multiple sexual partners,^77^ condomless sex^72^ and other risky sexual behaviours,^71 78^ as well as decreased antiretroviral therapy adherence and retention in care.^74^ There were few to no studies on the climate change events in this study and other STIs and certain aspects of HIV, including coinfections, discrimination and stigma.

#### Fertility care

Fertility care, encompassing pregnancy intentions and timing, family size and infertility, was studied by eight articles from across all regions. Fertility was studied in the context of extreme temperatures, floods and rainfall shocks.^79^,^8589^ Decreases in total fertility rates^79^ and reduced semen quality^80^ were reported to be associated with extreme temperatures. Increased birth rates were reported during below-average temperatures and above-average rainfall,^83^ but ideal family size lowered with exposure to higher temperatures in Africa.^81^

#### Harmful practices

Seven articles examined harmful practices such as forced marriages, and FGM and climate change. All the articles in this domain were from South-East Asian and African regions. Negative impacts were reported between extreme weather events such as storms,^27 28 32^ extreme temperatures,^29 33^ and drought^30 31^ and forced marriages and FGM.^27^,^33^ Of note, while most studies showed a greater risk of early and forced marriages and FGM, one study reported a 3% decrease in the risk of marriage between ages 12 and 17 in sub-Saharan Africa and a 4% decrease in India due to drought.^30^

#### Contraception

Only one article addressed contraception in this review, reporting associations between rainfall shocks and increased demand for modern and traditional contraceptives.^91^ No studies addressed associations between modern contraception preferences, whether long-acting or short-acting, or permanent as well as access, costs or user satisfaction.

#### Multiple SRHR domains

Five articles studied the interplay of climate change events and multiple SRHR domains. Three studies examined how fertility care, family planning and harmful practices are impacted by drought^86 88^ and extreme temperatures.^90^ One article analysed how floods impact SRHR services, fertility care including unwanted pregnancies, GBV, and maternal and newborn health through the lens of maternal death.^87^ Lastly, one article examined how more than three climatic events combined influence fertility care and contraception use.^89^

#### Main research gaps

Notably, no studies were conducted within the SRHR areas of induced abortion and reproductive cancers in the context of climate change, indicating research gaps that could be explored by future studies.

## Discussion

This scoping review is among the first to map peer-reviewed publications over the past three decades at the intersection of climate change and SRHR in LMICs across WHO regions worldwide. We identified 75 articles investigating the impact of climate change on SRHR. Most of these articles were published in 2018 and were conducted on populations in African, Western Pacific and South-East Asian regions. Most studies employed quantitative methodologies and targeted newborns and women of reproductive age. Maternal and newborn health was the most researched aspect of SRHR in the context of climate change; meanwhile, we did not find any studies exploring the impact of climate change on induced abortion or reproductive cancers. Extreme temperatures and drought were the most studied climate change phenomena, especially as they relate to maternal and newborn health.

The exponential increase in the articles published since 2018 indicates a growing recognition of the need to understand the impact of climate change on SRHR. This aligns with the growing attention on climate change and health as a global research priority, as demonstrated by the 2017/2018 WHO surveys conducted among national health services on health and climate change, involving 101 countries. These highlighted the profiled countries’ expected health impacts of climate change with the aim to raise awareness of health and climate linkages.^93^ The fact that most articles relied on data from cross-sectional surveys implies that the captured climate impacts are either from ongoing or past extreme weather events. This poses challenges in ascertaining some impact associations found due to the risk of recall or social desirability biases and other systematic errors, for example, regarding identifying links between forced marriage and floods in a community where early and forced marriage are already highly prevalent. Using health records from registries on maternal/newborn health, HIV, abortion or reproductive cancers could allow for longitudinal analysis and impact comparisons of extreme climatic events at different times with higher validity.

While the intersection of climate change and maternal and newborn health has received significant attention in published articles, including a recent call to action by several UN actors,^5^ a substantial amount (73%) of these articles focused on the impact of extreme temperatures. Moreover, most of these articles were from the Western Pacific region, particularly China. Conversely, there is limited literature on the impact of floods, rainfall shocks, drought and cyclones on maternal and newborn health in regions prone to these climate change events and where the risk of maternal and newborn morbidity and mortality due to climate change is higher such as in African and South-East Asian regions.^94^ Filling such research gaps in these regions is critical to inform tailored interventions and policies. Further, certain climate change and SRHR areas are predominantly studied in certain regions, perhaps due to heightened vulnerabilities in those regions and subsequent investments for research. For instance, floods and cyclones are well studied in South-East Asia, mainly Bangladesh, likely owing to their susceptibility to such events.^84^ Articles on HIV, GBV and drought from the African region reflect the substantial challenges related to HIV and drought in this region.^73^,^76^ A recent review by Logie *et al*^95^ revealed an association between extreme weather events—such as hurricanes, floods, drought and storms—and adverse HIV outcomes attributable to limited access to antiretroviral treatment and deteriorating mental health. Notably, very few articles focused on Latin America and the Caribbean, although many communities in this region are highly vulnerable to weather extremes and are dependent on weather-sensitive activities such as agriculture and tourism.^96^

This scarcity of studies looking at climate change events and contraception, abortion or reproductive cancers revealed in this review could be partly due to a lack of reliable data across LMICs.^97^ Addressing these data gaps is crucial in the context of climate change. Furthermore, despite only one article that exclusively studied climate change and harmful practices in Kenya,^31^ reliable organisational reports have continued to highlight the implications of climate change on early and forced marriages and FGM across the African region.^98^ While FGM is a practice deeply embedded in culture and social norms, it is also a requirement for marriage in many settings, therefore, an increase in FGM can be a precursor to early marriage in response to existential and livelihood threats posed by climate change.^31^ Certain SRHR domains such as abortion, GBV and harmful practices may require a special long-term framework to study in the context of climate change in LMICs given that reporting and documentation of such incidences may be hindered due to sociocultural limitations in certain communities. Longitudinal research and greater involvement of local researchers and local stakeholders could improve measurement.

In all regions, the predominant study populations were newborns and women of reproductive age. Subsequent studies could endeavour to broaden the scope of research at the intersection of climate change and SRHR in LMICs to include other demographics such as boys, men and individuals with diverse sexual orientations, gender identities/expressions and sex characteristics. Additionally, studies could explore these associations among migrant and indigenous populations. Across all populations, more attention could be given to the ‘rights’ aspect of SRHR.^99^ In essence, the achievement of SRHR relies on adopting a rights-based approach to health; a perspective that emphasises that all individuals have a right to make decisions regarding their bodies and should have access to services that uphold and support that right.^99^

### Implications for future research

Regarding the methods, several studies highlighted limitations related to the available health data for climate change research.^32 36 37^ This emphasises the need for improved climate-related routine health data collection. There were relatively very few articles that employed mixed quantitative and qualitative approaches where findings were merged and triangulated. Researchers could harness the power of both methods for a better understanding of climate change-related and SRHR-related topics. More registry-based data and longitudinal studies could also be helpful for studying rare impacts of climate change events on SRHR in LMICs, including those related to changes in air quality.

Over one-third of the articles were authored exclusively by authors from institutions in high-income countries, with limited involvement of institutions in LMICs. This may be due to imbalances of power and funding resources skewed towards high-income countries and, perhaps, an underappreciation of prioritising indigenous, context-specific knowledge in research.^100^ Considering that climate change disproportionately impacts LMICs, collaborating with local researchers in affected areas can be vital, especially when employing mixed methods and qualitative approaches. Moving forward, fostering cross-institutional collaborations between high-income countries and LMICs, as well as South-South partnerships, would not only enhance research capabilities and the validity, credibility and transferability of findings but also address ethical considerations^100^ and equitable partnerships in this critical field.

### Limitations

There are limitations to this review. First, given the lack of standard classification of climate change factors, there is a possibility that the search may have left out other potential intersection areas, for instance, climate change and SRHR-related infectious diseases and social conflicts. Relatedly, our categorisation of SRHR domains and climate change phenomena is but one way to classify these issues, there could be others. For example, we did not find studies on broader sexual or menstrual health which could alter this categorisation in the future. Second, we excluded studies on projections and predictions which could have provided valuable insights concerning the future impacts of climate change on SRHR. Similarly, we also excluded one important factor related to climate change: air pollution. Given the broad range of air pollution factors, including the confounding or mediating effects, it is complicated to determine the impact of air pollution attributable to climate change. These factors are outside the scope of this review and best placed as a separate standalone review. Third, since this is a scoping review, we leave it to future systematic reviews to conduct critical appraisals to assess the quality of extant research. Fourth, by focusing on studies in LMICs, we excluded studies on forcibly displaced people, asylum seekers, refugees and other migrants originating from LMICs but located in high-income countries.

## Conclusion

The findings of this scoping review underscore the growing, but still emerging, field of research that links climate change and SRHR in LMICs. We mapped existing studies and identified knowledge gaps that could guide future research priorities. In the context of climate change, maternal and newborn health was the most studied while potential climate change links to abortion, reproductive cancers and contraception were the least researched SRHR domains. The review underscores the urgent need to complement existing evidence with targeted research to fill knowledge gaps and strengthen the evidence base to guide interventions and policy development on SRHR and climate change.

## supplementary material

10.1136/bmjph-2024-001090 online supplemental file 1

10.1136/bmjph-2024-001090 online supplemental file 2

## Data Availability

All data relevant to the study are included in the article or uploaded as online supplemental information.
