# Supplementary material for: Climate change and sexual and reproductive health and rights research in low-income and middle-income countries: a scoping review
Source: BMJ Public Health. 2024 Aug 24;2(2):e001090. doi: 10.1136/bmjph-2024-001090 (PMC11816306; doi:10.1136/bmjph-2024-001090)
Supplement: online supplemental file 1 [file bmjph-2-2-s001.pdf]

Supplementary Material Table of Contents

Contents

Detailed literature search strategy (*p* 2-24

PRISMA-ScR Checklist (*p* 25-26)

Table A: Characteristics of included article characteristics, presented by SRHR domains (*p* 27-50)

Table B: List of 99 low- and middle-income countries where included studies were carried out (*p* 51)

Table C: List of 75 included articles showing first/corresponding authors' affiliations by institutions/country and authors' affiliations to any LMIC or country of study (*p* 52-62)

Detailed Literature Search Strategy

Databases:

1. Medline (Ovid)
2. Embase (embase.com)
3. Web of Science Core Collection (Clarivate Analytics)
4. CINAHL (Ebsco)
5. Google Scholar (scholar.google.com)
6. Global Index Medicus (https://www.globalindexmedicus.net/)

Total number of hits:

- Before deduplication: 23 338
- After deduplication: 14 674
-

De-duplication was done using the method described by Bramer et al[1], adding an additional step of comparing DOIs.

1. Medline

Interface: Ovid MEDLINE(R) and Epub Ahead of Print, In-Process & Other Non-Indexed Citations and Daily<br><br>Date of Search: 2023-09-06<br><br>Number of hits: 23 338<br><br>Comment: In Ovid, two or more words are automatically searched as phrases; i.e. no quotation marks are needed	Field labels<br><br>• exp/ = exploded MeSH term<br>• / = non exploded MeSH term<br>• .ti,ab,kf. = title, abstract and author keywords<br>• adjx = within x words, regardless of order<br>• * = truncation of word for alternate endings

Ovid MEDLINE(R) ALL <1946 to September 06, 2023>

1	Abortion, Criminal/	2143
2	exp Abortion, Induced/	42317
3	Birth Weight/ or exp Infant, Low Birth Weight/	74840

[1] Bramer WM, Giustini D, de Jonge GB, et al. De-duplication of database search results for systematic reviews in EndNote. *J Med Libr Assoc* 2016; 104(3), 240-243.

4	exp Breast Feeding/	43043
5	Circumcision, Female/	1635
6	exp Contraception/ or exp Contraceptive agents/ or exp Contraceptive devices/	117783
7	Domestic Violence/	7453
8	Family Characteristics/	29109
9	exp Fertility/	45394
10	exp "Embryonic and Fetal Development"/	306967
11	Follicular Atresia/	964
12	Gender-Based Violence/	520
13	Gender Equity/	480
14	exp Gender Identity/	23241
15	Gender Role/	225
16	Gestational Age/	88269
17	Gestational Weight Gain/	1202
18	Gravidity/ or Parity/	27563
19	exp HIV/ or exp HIV Infections/	345655
20	Infant Health/	1221
21	exp Infertility/	72549
22	exp Intimate Partner Violence/	12674
23	exp Labor Presentation/	7098
24	Lactation/	46832
25	Maternal Age/ or Paternal Age/	21411
26	Maternal-child health center/ or exp Reproductive health services/	45065
27	Maternal Health/ or Maternal welfare/ or Maternal Mortality/	19108
28	Midwifery/	20921
29	Ovarian Reserve/	1603
30	exp Perinatal Care/	11476
31	Peripartum Period/ or Postpartum Period/	31486

32	exp Pregnancy/ or exp Pregnancy Complications/ or Pregnancy Rate/ or exp Pregnancy Trimesters/	1021076
33	Pregnant Woman/	13373
34	Primary health care/ or Universal health care/	90094
35	Reproductive Health/ or Sexual Health/	6509
36	Reproductive Rights/	1112
37	Reproduction/ or exp Reproductive Behavior/ or Coitus/ or Ejaculation/ or exp Fertilization/ or Orgasm/ or exp Ovulation/ or Penile Erection/	143458
38	Reproductive Tract Infections/	642
39	Self Care/	35746
40	Sex/ or exp Sexuality/ or exp Sexual Development/ or exp Menstrual cycle/ or exp Sexual behavior/	260197
41	Sexism/	3163
42	Sex Workers/	2912
43	exp Sexual Dysfunction, Physiological/	32914
44	exp Sexual Dysfunctions, Psychological/	29595
45	exp Sexually Transmitted Diseases/	373347
46	exp Sex Offenses/	27024
47	Sex Education/	9209
48	Uterine Cervical Neoplasms/	83543
49	Women's Rights/	8821
50	(AIDS or chancre or chancroid or chlamydia or condylomata acuminata or donovanosis or genital herpes or genital tract infection* or genital wart? or gonorrhea? or granuloma or great pox or herpes genital* or HIV or reproductive tract infection* or sexually transmitted disease? or sexually transmitted infection? or syphilis or venereal disease? or venereal wart?).ti,ab,kf.	538971
51	(adrenarche or climacteric* or fertile period* or follicular phase or luteal phase or menarche or menopause or menstrua* or ovarian reserve or perimenopause or postmenopause or premenopause or puberty).ti,ab,kf.	137826
52	(bisexualit* or celibacy or coitus or condomless sex or courtship* or ejaculation or extramarital relation* or heterosexualit* or homosexualit* or masturbation or orgasm or penile erection or protected sex or responsible sex or safe sex or sexism or sexualit* or sex* abstinence or sexual development* or sexual intercourse or sexual orientation or unprotected intercourse or unprotected sex or unsafe sex).ti,ab,kf.	70495
53	(dyspareunia or erectile dysfunction or gender dysphoria or gender disorder* or male impotence or premature ejaculation* or psychosexual disorder* or psychosexual dysfunction* or sexual disorder* or sexual dysfuntion* or sexual impotence or vaginismus or venogenic impotence).ti,ab,kf.	27872
54	(maternal health service* or self care or primary health care or primary healthcare or universal health care or universal healthcare).ti,ab,kf.	66958
55	((child or early or forced) adj marriage).ti,ab,kf.	1187

56	(gender adj3 (equalit* or equity or identit* or norm? or role or violence)).ti,ab,kf.	17539
57	(women* adj2 (liberation or right* or status)).ti,ab,kf.	10038
58	((sexual* or reproductive) adj3 (behavio?r* or health or healthcare or justice* or right?)).ti,ab,kf.	76953
59	((domestic or intimate partner? or sexual* or spouse? or women) adj3 (abuse or violence)).ti,ab,kf.	40510
60	((infant* or matern* or mother* or newborn* or paternal or perinatal or prenatal or pre natal) adj3 (age or death* or disease* or injur* or health or morbidity or mortality or obesity or welfare)).ti,ab,kf.	194567
61	((fetal or fetus* or foetus*) adj2 (alcohol effect* or alcohol spectrum disorder or alcohol syndrome or anoxia or death* or demise or disease* or edema or growth restriction or growth retardation or hydrops or hypoxia or malnutrition or macrosomia* or nutrition disorder* or resorption*)).ti,ab,kf.	30152
62	(sex* adj2 (education or harassment* or offense* or work*)).ti,ab,kf.	27300
63	(fertility adj3 (control or effect* or female? or woman or women or outcome?)).ti,ab,kf.	20951
64	(abortion* or antenatal or birth attendant or birth control or birth setting* or birth outcome* or birth spacing or birth weight or breast feed* or breastfeed* or breastfed or breast fed or breech presentation or childbirth* or conception or contraception* or contraceptive* or early pregnancy loss* or expectant mother* or family planning or (fertilization adj2 inhibition) or fetal development* or fetal malpresentation or fetal presentation or fetal viability or fetal weight or follicular atresia or gestation* or gravidity or gynecolog* or infertility or labor presentation or lactation or livebirth or midwife* or midwives or miscarriage* or neonatal death* or obstetric* or ovulation inhibition or ovulation suppression or parity or parturition or perinatal or peripartum or postabortion* or postpartum or postnatal or pregnan* or puerperium or sterilization or stillbirth or term birth).ti,ab,kf.	1266274
65	(clitoridectom* or clitorectom* or female circumcision* or female genital cutting or female genital mutilation or infibulation).ti,ab,kf.	2061
66	((cervix or cervical) adj2 (cancer* or neoplasm*)).ti,ab,kf.	67806
67	or/1-66	3173954
68	exp Air Pollutants/	108022
69	Air Pollution/	38260
70	exp Climate Change/	28245
71	Cyclonic Storms/	2890
72	Droughts/	11379
73	El Nino-Southern Oscillation/	524
74	Greenhouse Effect/	6072
75	Greenhouse Gases/	2077
76	Hot Temperature/	123647
77	Carbon Footprint/	974
78	exp Extreme Weather/	268

79	Extreme Heat/	537
80	Floods/	3735
81	Food Insecurity/	1186
82	Rain/	11853
83	Wildfires/	1024
84	(climate adj3 (adaption* or associat* or change* or changing or crisis or induce* or migrant* or model? or predict* or refugee* or resilience or sensitivity)).ti,ab,kf.	63613
85	(air pollution or air pollutants or air quality or carbon footprint? or drought* or dry corridor* or El Nino or environmental degradation or flood? or food insecur* or global environmental change? or global heating or global warming or greenhouse gas* or greenhouse effect? or heavy precipitation or heatwave* or heat wave* or hurricane* or La nina or landslide* or land slide* or lightning strike* or megadrought* or megafire* or polar amplification or rain? or rainfall or (sea ice adj1 shrink*) or (sea level adj1 ris*) or sea surface warming or storm? or typhoon* or wildfire* or wild fire*).ti,ab,kf.	184996
86	((extreme or destructive or severe) adj3 (heat or storm* or temperature* or weather? or wind?)).ti,ab,kf.	9256
87	((destructive or extreme or global or high or hot or severe or warm) adj2 temperature*).ti,ab,kf.	74896
88	(arctic amplification or arctic shrinkage or avalanche* or brush fire* or carbon offset* or carbon sequestration or carbon sink or carbon sinks or cyclon* or deglaciation or desertification or forest fire* or emissions reduc* or emissions trading or global radiation or hurricane* or ice mass loss* or Kyoto Protocol or mudslide or mud slide* or Paris Accord or polar amplification or thermohaline circulation).ti,ab,kf.	20016
89	(chlorofluorocarbon* adj2 (release or concentration* or atmosphere*)).ti,ab,kf.	9
90	(earth adj2 warming).ti,ab,kf.	31
91	((glacial or glacier or ice cap or permafrost or polar ice) adj3 (retreat* or melt*)).ti,ab,kf.	887
92	(ozone adj2 hole).ti,ab,kf.	152
93	or/68-92	514222
94	Medically Underserved Area/ or Developing Countries/ or Rural Health/ or Rural Population/	171519
95	Afghanistan/ or Albania/ or Algeria/ or Angola/ or Argentina/ or Armenia/ or Azerbaijan/ or Bangladesh/ or Benin/ or "Republic of Belarus"/ or Belize/ or Bhutan/ or Bolivia/ or "Bosnia and Herzegovina"/ or Botswana/ or Brazil/ or Bulgaria/ or Burkina Faso/ or Burundi/ or Cambodia/ or Cameroon/ or Cabo Verde/ or Central African Republic/ or Chad/ or exp China/ or Colombia/ or Comoros/ or Congo/ or Costa Rica/ or Cote d'Ivoire/ or Cuba/ or Djibouti/ or Dominican Republic/ or Ecuador/ or Egypt/ or El Salvador/ or Eritrea/ or Equatorial Guinea/ or Ethiopia/ or Fiji/ or Gabon/ or Gambia/ or Georgia/ or Ghana/ or Grenada/ or Guatemala/ or Guinea/ or Guinea-Bissau/ or Guyana/ or Haiti/ or Honduras/ or India/ or Indonesia/ or Iran/ or Iraq/ or Jamaica/ or Jordan/ or Kazakhstan/ or Kenya/ or "Democratic People's Republic of Korea"/ or Kosovo/ or Kyrgyzstan/ or Laos/ or Lebanon/ or Lesotho/ or Liberia/ or Libya/ or Macedonia/ or Madagascar/ or Malaysia/ or Malawi/ or Mali/ or Mauritania/ or Micronesia/ or Mauritius/ or Mexico/ or Moldova/ or Mongolia/ or Montenegro/ or Morocco/ or Mozambique/ or Myanmar/ or Namibia/ or Nepal/ or Nicaragua/ or Niger/ or Nigeria/ or Pakistan/ or Palau/ or "Papua New Guinea"/ or Paraguay/ or Peru/ or Philippines/ or Romania/ or Russia/ or Rwanda/ or Saint Lucia/ or "Saint Vincent and the Grenadines"/ or "Independent State of Samoa"/ or "Sao Tome and Principe"/ or Senegal/ or Serbia/ or Sierra Leone/ or exp Melanesia/ or Sri Lanka/ or Somalia/ or South Sudan/ or Sudan/ or South Africa/ or Suriname/ or Swaziland/ or Syria/ or Tajikistan/ or Tanzania/ or Timor-Leste/ or Thailand/ or Togo/ or	1245658

	Tonga/ or Tunisia/ or Turkey/ or Turkmenistan/ or Uganda/ or Ukraine/ or exp USSR/ or Uzbekistan/ or Vanuatu/ or Venezuela/ or Vietnam/ or Yemen/ or Zambia/ or Zimbabwe/	
96	(Africa or Asia or Caribbean or West Indies or South America or Latin America or Central America).tw.	240202
97	(Afghanistan or Albania* or Algeria* or American Samoa or Angola* or Argentina or Armenia* or Azerbaijan or Bangladesh or Benin or Byelarus or Byelorussian or Belarus or Belorussian or Belorussia or Belize or Bhutan or Bolivia* or Bosnia* or Herzegovina or Hercegovina or Botswana or Brazil* or Brasil* or Bulgaria* or Burkina Faso or Burkina Fasso or Burundi or Urundi or Cambodia* or Khmer Republic or Kampuchea or Cameroon or Cameroons or Cameron or Camerons or Cape Verde or Cabo Verde or Central African Republic or Chad or China or Colombia* or Comoros or Comoro Islands or Comores or Congo or Costa Rica or Cote d'Ivoire or Ivory Coast or Cuba or Djibouti or Dominica or Dominican Republic or East Timor or East Timur or Timor Leste or Ecuador or Egypt* or El Salvador or Eritrea* or Equatorial Guinea or Eswatini* or Ethiopia* or Fiji or Gabon or Gambia* or Gaza or Georgia Republic or Georgian Republic or Ghana or Grenada or Guatemala* or Guinea or Guyana or Haiti or Honduras or India or Indonesia* or Iran or Iraq or Jamaica* or Jordan* or Kazakhstan or Kenya* or Kiribati or Democratic People's Republic of Korea* or North Korea* or Kosovo or Kyrgyz Republic or Lao PDR or Laos or Lebanon or Lesotho or Liberia* or Libya* or Macedonia* or Madagascar or Malaysia* or Malaya* or Malay or Malawi or Mali or Maldives or Marshall Islands or Mauritania* or Mauritius or Mexico or Mehico or Micronesia* or Middle East or Moldova or Moldovia* or Moldovian* or Mongolia or Montenegro or Morocco or Mozambique or Mocambique or Myanmar or Namibia* or Nauru or Nepal or Nicaragua or Niger or Nigeria* or Pakistan* or Palau or Palestine* or Paraguay or Peru* or Philippines or Philipines or Phillipines or Phillippines or Romania* or Rumania* or Roumania* or Russia or Russian or Rwanda or Ruanda or Saint Lucia or St Lucia or Saint Vincent or St Vincent or Grenadines or Samoa or Samoan Islands or Sao Tome or Senegal or Serbia* or Sierra Leone or Spanish Guinea or Sri Lanka or Ceylon or Solomon or USSR or Soviet Union or "Union of Soviet Socialist Republics" or Somalia* or South Africa* or Sudan or Suriname or Surinam or Swaziland or Syria or Syrian Arab Republic or Tajikistan or Tadzhikistan or Tadjikistan or Tadzhik or Tanzania or Thailand or Togo or Togolese Republic or Tonga or Tunisia* or Turkey or Turkiye or Turkmenistan or Tuvalu or Uganda or Ukrain* or Uzbekistan or Uzbek or Vanuatu or Venezuela or Vietnam or Viet Nam or West Bank or Yemen or Zambia or Zimbabwe).ti,ab,kf.	1482789
98	((developing or emerging or less* developed or under developed or underdeveloped or middle income or low* income or third-world or underserved or under served or deprived or poor*) adj3 (countr* or nation? or population? or world or economy or economies)).ti,ab.	146448
99	(low* adj2 (countr* or gdp or gnp or gross domestic or gross national)).ti,ab.	15548
100	((low* or middle*) adj5 (countr* or nation*)).ti,ab,kf.	62869
101	((rural or remote or nonmetropolitan or non-metropolitan or underserved or under served or deprived or shortage) adj (communit$ or count$ or area? or region? or province? or district?)).ti,ab.	81333
102	(Global South or LIC or LMIC* or LMICs or MIC or South-South or rural health* or rural population*).ti,ab,kf.	81565
103	or/94-102	2228890
104	67 and 93 and 103	6422
105	limit 104 to yr="1994 -Current"	5750

2. Embase

Interface: embase.com<br><br>Date of Search: 06 September 2023<br><br>Number of hits: 8102<br><br>Comment: Emtree is the controlled vocabulary in Embase	Field labels<br><br>• /exp = exploded Emtree term<br>• /de = non exploded Emtree term<br>• ti,ab,kw = title, abstract and author keywords<br>• NEAR/x = within x words, regardless of order<br>• * = truncation of word for alternate endings

No.	Query	Results
#87	#59 AND #77 AND #85 AND [1994-2023]/py	6980
#86	#59 AND #77 AND #85	7256
#85	#78 OR #79 OR #80 OR #81 OR #82 OR #83 OR #84	2033465
#84	'global south':ti,ab,kw OR lic:ti,ab,kw OR lmic*:ti,ab,kw OR lmics:ti,ab,kw OR mic:ti,ab,kw OR 'south south':ti,ab,kw OR 'rural health*':ti,ab,kw OR 'rural population*':ti,ab,kw	105268
#83	((rural OR remote OR nonmetropolitan OR 'non metropolitan' OR underserved OR 'under served' OR deprived OR shortage) NEXT/1 (communit? OR count? OR area$ OR region$ OR province$ OR district$)):ti,ab	87982
#82	((low* OR middle*) NEAR/5 (countr* OR nation*)):ti,ab,kw	77933
#81	(low* NEAR/2 (countr* OR gdp OR gnp OR 'gross domestic' OR 'gross national')):ti,ab	19715
#80	africa:ti,ab OR asia:ti,ab OR caribbean:ti,ab OR 'west indies':ti,ab OR 'south america':ti,ab OR 'latin america':ti,ab OR 'central america':ti,ab	302703
#79	'afghanistan'/de OR 'albania'/de OR 'algeria'/de OR 'angola'/de OR 'argentina'/de OR 'armenia'/de OR 'azerbaijan'/de OR 'bangladesh'/de OR 'benin'/de OR 'belarus'/de OR 'belize'/de OR 'bhutan'/de OR 'bolivia'/de OR 'bosnia and herzegovina'/exp OR 'botswana'/de OR 'brazil'/exp OR 'bulgaria'/de OR 'burkina faso'/de OR 'burundi'/de OR 'cambodia'/de OR 'cameroon'/de OR 'cape verde'/de OR 'central african republic'/de OR 'chad'/de OR 'china'/exp OR 'colombia'/de OR 'comoros'/de OR 'congo'/de OR 'costa rica'/de OR 'cote divoire' OR 'cuba'/de OR 'djibouti'/de OR 'dominican republic'/de OR 'ecuador'/de OR 'egypt'/de OR 'el salvador'/de OR 'eritrea'/de OR 'equatorial guinea'/de OR 'ethiopia'/de OR 'fiji'/de OR 'gabon'/de OR 'gambia'/de OR 'georgia (republic)'/exp OR 'ghana'/de OR 'grenada'/de OR 'guatemala'/de OR 'guinea'/de OR 'guinea bissau'/de OR 'guyana'/de OR 'haiti'/de OR 'honduras'/de OR 'india'/exp OR 'indonesia'/exp OR 'iran'/de OR 'iraq'/exp OR 'jamaica'/de OR 'jordan'/de OR 'kazakhstan'/de OR 'kenya'/de OR 'north korea'/de OR 'kosovo'/de OR 'kyrgyzstan'/de OR 'laos'/de OR 'lebanon'/de OR 'lesotho'/de OR 'liberia'/de OR 'libyan arab jamahiriya'/de OR 'republic of north macedonia'/de OR 'madagascar'/de OR 'malaysia'/exp OR 'malawi'/de OR 'mali'/de OR 'mauritania'/de OR 'federated states of micronesia'/de OR 'mauritius'/de OR 'mexico'/exp OR 'moldova'/de OR 'mongolia'/de OR 'montenegro (republic)'/de OR 'morocco'/de OR 'mozambique'/de OR 'myanmar'/de OR 'namibia'/de OR 'nepal'/de OR 'nicaragua'/de OR 'niger'/de OR 'nigeria'/de OR 'pakistan'/exp OR 'palau'/de OR 'papua new guinea'/de OR 'paraguay'/de OR 'peru'/de OR 'philippines'/de OR 'romania'/de OR 'russian federation'/exp OR 'rwanda'/de OR 'saint lucia'/de OR 'saint vincent and the grenadines'/de OR 'samoa'/de OR 'sao tome and principe'/de OR 'senegal'/de OR 'serbia'/exp OR 'sierra leone'/de OR 'melanesia'/de OR 'sri lanka'/de OR 'somalia'/exp OR 'south sudan'/de OR 'sudan'/de OR 'south africa'/de OR 'suriname'/de OR 'eswatini'/de OR 'syrian arab republic'/de OR 'tajikistan'/de OR 'tanzania'/de OR 'timor leste'/de OR 'thailand'/de OR 'togo'/de OR 'tonga'/de OR 'tunisia'/de OR 'turkey (republic)'/de OR 'turkmenistan'/de OR 'uganda'/de OR 'ukraine'/exp OR 'ussr'/exp OR 'uzbekistan'/exp OR 'vanuatu'/de OR 'venezuela'/de OR 'viet nam'/de OR 'yemen'/de OR 'zambia'/de OR 'zimbabwe'/de	1624128

#78	'developing country'/de OR 'low income country'/de OR 'middle income country' OR 'rural health'/de OR 'rural population'/de	177573
#77	#60 OR #61 OR #62 OR #63 OR #64 OR #65 OR #66 OR #67 OR #68 OR #69 OR #70 OR #71 OR #72 OR #73 OR #74 OR #75 OR #76	499403
#76	(ozone NEAR/2 hole):ti,ab,kw	189
#75	((glacial OR glacier OR 'ice cap' OR permafrost OR 'polar ice') NEAR/3 (retreat* OR melt*)):ti,ab,kw	866
#74	(earth NEAR/2 warming):ti,ab,kw	47
#73	(chlorofluorocarbon* NEAR/2 (release OR concentration* OR atmosphere*)):ti,ab,kw	15
#72	'arctic amplification':ti,ab,kw OR 'arctic shrinkage':ti,ab,kw OR avalanche*:ti,ab,kw OR 'brush fire*':ti,ab,kw OR 'carbon offset*':ti,ab,kw OR 'carbon sequestration':ti,ab,kw OR 'carbon sink':ti,ab,kw OR 'carbon sinks':ti,ab,kw OR cyclon*:ti,ab,kw OR deglaciation:ti,ab,kw OR desertification:ti,ab,kw OR 'forest fire*':ti,ab,kw OR 'emissions reduc*':ti,ab,kw OR 'emissions trading':ti,ab,kw OR 'global radiation':ti,ab,kw OR hurricane*:ti,ab,kw OR 'ice mass loss*':ti,ab,kw OR 'kyoto protocol':ti,ab,kw OR mudslide:ti,ab,kw OR 'mud slide*':ti,ab,kw OR 'paris accord':ti,ab,kw OR 'polar amplification':ti,ab,kw OR 'thermohaline circulation':ti,ab,kw	21450
#71	((destructive OR extreme OR global OR high OR hot OR severe OR warm) NEAR/2 temperature*):ti,ab,kw	68187
#70	((extreme OR destructive OR severe) NEAR/3 (heat OR storm* OR temperature* OR weather$ OR wind$)):ti,ab,kw	9735
#69	'air pollution':ti,ab,kw OR 'air pollutants':ti,ab,kw OR 'air quality':ti,ab,kw OR 'carbon footprint$':ti,ab,kw OR drought*:ti,ab,kw OR 'dry corridor*':ti,ab,kw OR 'el nino':ti,ab,kw OR 'environmental degradation':ti,ab,kw OR flood$:ti,ab,kw OR 'food insecur*':ti,ab,kw OR 'global environmental change$':ti,ab,kw OR 'global heating':ti,ab,kw OR 'global warming':ti,ab,kw OR 'greenhouse gas*':ti,ab,kw OR 'greenhouse effect$':ti,ab,kw OR 'heavy precipitation':ti,ab,kw OR heatwave*:ti,ab,kw OR 'heat wave*':ti,ab,kw OR hurricane*:ti,ab,kw OR 'la nina':ti,ab,kw OR landslide*:ti,ab,kw OR 'land slide*':ti,ab,kw OR 'lightning strike*':ti,ab,kw OR megadrought*:ti,ab,kw OR megafire*:ti,ab,kw OR 'polar amplification':ti,ab,kw OR rain$:ti,ab,kw OR rainfall:ti,ab,kw OR (('sea ice' NEAR/1 shrink*):ti,ab,kw) OR (('sea level' NEAR/1 ris*):ti,ab,kw) OR 'sea surface warming':ti,ab,kw OR storm$:ti,ab,kw OR typhoon*:ti,ab,kw OR wildfire*:ti,ab,kw OR 'wild fire*':ti,ab,kw	216972
#68	(climate NEAR/3 (adaption* OR associat* OR change* OR changing OR crisis OR induce* OR migrant* OR model$ OR predict* OR refugee* OR resilience OR sensitivity)):ti,ab,kw	62114
#67	'food insecurity'/exp	6777
#66	'extreme weather'/exp	827
#65	'carbon footprint'/de	10540
#64	'high temperature'/de	35229
#63	'greenhouse effect'/de	16752
#62	'drought'/de OR 'el nino'/de OR 'flooding'/de OR 'hurricane'/de OR 'rain'/de OR 'wildfire'/exp	51351
#61	'climate change'/exp	53483
#60	'air pollution'/exp	200668
#59	#1 OR #2 OR #3 OR #4 OR #5 OR #6 OR #7 OR #8 OR #9 OR #10 OR #11 OR #12 OR #13 OR #14 OR #15 OR #16 OR #17 OR #18 OR #19 OR #20 OR #21 OR #22 OR #23 OR #24 OR #25 OR #26 OR #27 OR #28 OR #29 OR #30 OR #31 OR #32 OR #33 OR #34 OR #35 OR #36 OR #37 OR #38 OR #39 OR #40 OR #41 OR #42 OR #43 OR #44 OR #45 OR #46 OR #47 OR #48 OR #49 OR #50 OR #51 OR #52 OR #53 OR #54 OR #55 OR #56 OR #57 OR #58	4586972

#58	clitoridectom*:ti,ab,kw OR clitorectom*:ti,ab,kw OR 'female circumcision*':ti,ab,kw OR 'female genital cutting':ti,ab,kw OR 'female genital mutilation':ti,ab,kw OR infibulation:ti,ab,kw OR (((cervix OR cervical) NEAR/2 (cancer* OR neoplasm*)):ti,ab,kw)	93057
#57	abortion*:ti,ab,kw OR antenatal:ti,ab,kw OR 'birth attendant':ti,ab,kw OR 'birth control':ti,ab,kw OR 'birth setting*':ti,ab,kw OR 'birth outcome*':ti,ab,kw OR 'birth spacing':ti,ab,kw OR 'birth weight':ti,ab,kw OR 'breast feed*':ti,ab,kw OR breastfeed*:ti,ab,kw OR breastfed:ti,ab,kw OR 'breast fed':ti,ab,kw OR 'breech presentation':ti,ab,kw OR childbirth*:ti,ab,kw OR conception:ti,ab,kw OR contraception*:ti,ab,kw OR contraceptive*:ti,ab,kw OR 'early pregnancy loss*':ti,ab,kw OR 'expectant mother*':ti,ab,kw OR 'family planning':ti,ab,kw OR ((fertilization NEAR/2 inhibition):ti,ab,kw) OR 'fetal development*':ti,ab,kw OR 'fetal malpresentation':ti,ab,kw OR 'fetal presentation':ti,ab,kw OR 'fetal viability':ti,ab,kw OR 'fetal weight':ti,ab,kw OR 'follicular atresia':ti,ab,kw OR gestation*:ti,ab,kw OR gravidity:ti,ab,kw OR gynecolog*:ti,ab,kw OR infertility:ti,ab,kw OR 'labor presentation':ti,ab,kw OR lactation:ti,ab,kw OR livebirth:ti,ab,kw OR midwife*:ti,ab,kw OR midwives:ti,ab,kw OR miscarriage*:ti,ab,kw OR 'neonatal death*':ti,ab,kw OR obstetric*:ti,ab,kw OR 'ovulation inhibition':ti,ab,kw OR 'ovulation suppression':ti,ab,kw OR parity:ti,ab,kw OR parturition:ti,ab,kw OR perinatal:ti,ab,kw OR peripartum:ti,ab,kw OR postabortion*:ti,ab,kw OR postpartum:ti,ab,kw OR postnatal:ti,ab,kw OR pregnan*:ti,ab,kw OR puerperium:ti,ab,kw OR sterilization:ti,ab,kw OR stillbirth:ti,ab,kw OR 'term birth':ti,ab,kw	1654278
#56	(fertility NEAR/3 (control OR effect* OR female$ OR woman OR women OR outcome$)):ti,ab,kw	20456
#55	(((fetal OR fetus* OR foetus*) NEAR/2 ('alcohol effect*' OR 'alcohol spectrum disorder' OR 'alcohol syndrome' OR anoxia OR death* OR demise OR disease* OR edema OR 'growth restriction' OR 'growth retardation' OR hydrops OR hypoxia OR malnutrition OR macrosomia* OR 'nutrition disorder*' OR resorption*)):ti,ab,kw) OR ((sex* NEAR/2 (education OR harassment* OR offense* OR work*)):ti,ab,kw)	77610
#54	((infant* OR matern* OR mother* OR newborn* OR paternal OR perinatal OR prenatal OR 'pre natal') NEAR/3 (age OR death* OR disease* OR injur* OR health OR morbidity OR mortality OR obesity OR welfare)):ti,ab,kw	242341
#53	((domestic OR 'intimate partner$' OR sexual* OR spouse$ OR women) NEAR/3 (abuse OR violence)):ti,ab,kw	49288
#52	((sexual* OR reproductive) NEAR/3 (behavio$r* OR health OR healthcare OR justice* OR right$)):ti,ab,kw	96968
#51	(women* NEAR/2 (liberation OR right* OR status)):ti,ab,kw	8089
#50	(gender NEAR/3 (equalit* OR equity OR identit* OR norm$ OR role OR violence)):ti,ab,kw	20826
#49	'maternal health service*':ti,ab,kw OR 'self care':ti,ab,kw OR 'primary health care':ti,ab,kw OR 'primary healthcare':ti,ab,kw OR 'universal health care':ti,ab,kw OR 'universal healthcare':ti,ab,kw OR (((child OR early OR forced) NEXT/1 marriage):ti,ab,kw)	83687
#48	dyspareunia:ti,ab,kw OR 'erectile dysfunction':ti,ab,kw OR 'gender dysphoria':ti,ab,kw OR 'gender disorder*':ti,ab,kw OR 'male impotence':ti,ab,kw OR 'premature ejaculation*':ti,ab,kw OR 'psychosexual disorder*':ti,ab,kw OR 'psychosexual dysfunction*':ti,ab,kw OR 'sexual disorder*':ti,ab,kw OR 'sexual dysfuntion*':ti,ab,kw OR 'sexual impotence':ti,ab,kw OR vaginismus:ti,ab,kw OR 'venogenic impotence':ti,ab,kw	46811
#47	bisexualit*:ti,ab,kw OR celibacy:ti,ab,kw OR coitus:ti,ab,kw OR 'condomless sex':ti,ab,kw OR courtship*:ti,ab,kw OR ejaculation:ti,ab,kw OR 'extramarital relation*':ti,ab,kw OR heterosexualit*:ti,ab,kw OR homosexualit*:ti,ab,kw OR masturbation:ti,ab,kw OR orgasm:ti,ab,kw OR 'penile erection':ti,ab,kw OR 'protected sex':ti,ab,kw OR 'responsible sex':ti,ab,kw OR 'safe sex':ti,ab,kw OR sexism:ti,ab,kw OR sexualit*:ti,ab,kw OR 'sex* abstinence':ti,ab,kw OR 'sexual development*':ti,ab,kw OR 'sexual intercourse':ti,ab,kw OR 'sexual orientation':ti,ab,kw OR 'unprotected intercourse':ti,ab,kw OR 'unprotected sex':ti,ab,kw OR 'unsafe sex':ti,ab,kw	93317
#46	adrenarche:ti,ab,kw OR climacteric*:ti,ab,kw OR 'fertile period*':ti,ab,kw OR 'follicular phase':ti,ab,kw OR 'luteal phase':ti,ab,kw OR menarche:ti,ab,kw OR menopause:ti,ab,kw OR menstrua*:ti,ab,kw OR 'ovarian reserve':ti,ab,kw OR perimenopause:ti,ab,kw OR postmenopause:ti,ab,kw OR premenopause:ti,ab,kw OR puberty:ti,ab,kw	193063

#45	aids:ti,ab,kw OR chancre:ti,ab,kw OR chancroid:ti,ab,kw OR chlamydia:ti,ab,kw OR 'condylomata acuminata':ti,ab,kw OR donovanosis:ti,ab,kw OR 'genital herpes':ti,ab,kw OR 'genital tract infection*':ti,ab,kw OR 'genital wart$':ti,ab,kw OR gonorrhea$:ti,ab,kw OR granuloma:ti,ab,kw OR 'great pox':ti,ab,kw OR 'herpes genital*':ti,ab,kw OR hiv:ti,ab,kw OR 'reproductive tract infection*':ti,ab,kw OR 'sexually transmitted disease$':ti,ab,kw OR 'sexually transmitted infection$':ti,ab,kw OR syphilis:ti,ab,kw OR 'venereal disease$':ti,ab,kw OR 'venereal wart$':ti,ab,kw	678006
#44	'womens rights'/exp	7939
#43	'uterine cervix tumor'/exp	142252
#42	'sexual education'/de	14039
#41	'sexual crime'/de OR 'sexual assault'/exp	49806
#40	'sexually transmitted diseases'/exp	117751
#39	'sexual dysfunction'/exp	96970
#38	'sex worker'/de	3225
#37	'sexism'/de	4188
#36	'sex'/exp OR 'menstrual cycle'/exp OR 'sexual behavior'/exp	565888
#35	'self care'/de	71764
#34	'genital tract infection'/exp	22773
#33	'reproduction'/de OR 'reproductive behavior'/de OR 'coitus'/de OR 'ejaculation'/de OR 'fertilization'/de OR 'penis erection'/de	169953
#32	'reproductive rights'/de	1466
#31	'reproductive health'/de OR 'sexual health'/exp	39095
#30	'primary health care'/de OR 'universal health care'/de	77327
#29	'pregnant woman'/de	104322
#28	'pregnancy'/exp OR 'pregnancy disorder'/exp OR 'pregnancy rate'/de	1231633
#27	'perinatal period'/de OR 'puerperium'/de	89242
#26	'postnatal care'/exp OR 'perinatal care'/de	148380
#25	'ovarian reserve'/de	8212
#24	'midwife'/exp	36416
#23	'maternal care'/de OR 'maternal welfare'/de OR 'maternal mortality'/de	59394
#22	'maternal child health care'/de OR 'family service'/de OR 'maternal health service'/de OR 'family planning'/de	47755
#21	'parental age'/exp	49005
#20	'lactation'/de	61879
#19	'infertility'/exp	147844
#18	'human immunodeficiency virus'/exp OR 'human immunodeficiency virus infection'/exp	539211

#17	'parity'/de	44810
#16	'gestational weight gain'/de	4208
#15	'gestational age'/de	172207
#14	'sex role'/de	5500
#13	'gender identity'/de OR 'gender'/exp	360470
#12	'gender equity'/de	1126
#11	'gender based violence'/exp	1459
#10	'ovary follicle atresia'/de	2073
#9	'prenatal development'/exp	258114
#8	'fertility'/exp	92473
#7	'family structure'/de	577
#6	'domestic violence'/exp	71793
#5	'contraception'/exp OR 'contraceptive agents'/exp OR 'contraceptive devices'/exp	348547
#4	'female genital mutilation'/exp	2638
#3	'breast feeding'/exp	64552
#2	'birth weight'/de OR 'low birth weight'/exp	145382
#1	'induced abortion'/exp	38887

3. Web of Science Core Collection

Interface: Clarivate Analytics<br><br>Editions = A&HCI , ESCI , SCI-EXPANDED , SSCI<br><br>Date of Search: 06 September 2023<br><br>Number of hits: 7037	Field labels<br><br>• TS/Topic = title, abstract, author keywords and Keywords Plus<br>• NEAR/x = within x words, regardless of order<br>• * = truncation of word for alternate endings<br><br>Note: the *Exact search*-function was used for all the searches

#	Search Query	Results
1	TS=(AIDS OR chancre OR chancroid OR chlamydia OR "condylomata acuminata" OR donovanosis OR "genital herpes" OR "genital tract infection*" OR "genital wart$" OR gonorrhea$ OR granuloma OR "great pox" OR "herpes genital*" OR HIV OR "reproductive tract infection*" OR "sexually transmitted disease$" OR "sexually transmitted infection$" OR syphilis OR "venereal disease$" OR "venereal wart$")	598898
2	TS=(adrenarche OR climacteric* OR "fertile period*" OR "follicular phase" OR "luteal phase" OR menarche OR menopause OR menstrua* OR "ovarian reserve" OR perimenopause OR postmenopause OR premenopause OR puberty)	141497
3	TS=(bisexualit* OR celibacy OR coitus OR "condomless sex" OR courtship* OR ejaculation OR "extramarital relation*" OR heterosexualit* OR homosexualit* OR masturbation OR orgasm OR "penile erection" OR "protected sex" OR "responsible sex" OR "safe sex" OR sexism OR sexualit* OR "sex* abstinence" OR "sexual development*" OR "sexual intercourse" OR "sexual orientation" OR "unprotected intercourse" OR "unprotected sex" OR "unsafe sex")	121671
4	TS=(dyspareunia OR "erectile dysfunction" OR "gender dysphoria" OR "gender disorder*" OR "male impotence" OR "premature ejaculation*" OR "psychosexual disorder*" OR "psychosexual dysfunction*" OR "sexual disorder*" OR "sexual dysfuntion*" OR "sexual impotence" OR vaginismus OR "venogenic impotence")	34626
5	TS=("maternal health service*" OR "self care" OR "primary health care" OR "primary healthcare" OR "universal health care" OR "universal healthcare")	65244
6	TS=((child OR early OR forced) NEAR/0 marriage)	1832
7	TS=(gender NEAR/2 (equalit* OR equity OR identit* OR norm$ OR role OR violence))	45748
8	TS=(women* NEAR/1 (liberation OR right* OR status))	12479
9	TS=((sexual* OR reproductive) NEAR/2 (behavio$r* OR health OR healthcare OR justice* OR right$))	98756
10	TS=((domestic OR "intimate partner$" OR sexual* OR spouse$ OR women) NEAR/2 (abuse OR violence))	71478
11	TS=((infant* OR matern* OR mother* OR newborn* OR paternal OR perinatal OR prenatal OR "pre natal") NEAR/2 (age OR death* OR disease* OR injur* OR health OR morbidity OR mortality OR obesity OR welfare))	168703
12	TS=((fetal OR fetus* OR foetus*) NEAR/1 ("alcohol effect*" OR "alcohol spectrum disorder" OR "alcohol syndrome" OR anoxia OR death* OR demise OR disease* OR edema OR "growth restriction" OR "growth retardation" OR hydrops OR hypoxia OR malnutrition OR macrosomia* OR "nutrition disorder*" OR resorption*))	28338
13	TS=(sex* NEAR/1 (education OR harassment* OR offense* OR work*))	37834
14	TS=(fertility NEAR/2 (control OR effect* OR female$ OR woman OR women OR outcome$))	17855
15	TS=(abortion* OR antenatal OR "birth attendant" OR "birth control" OR "birth setting*" OR "birth outcome*" OR "birth spacing" OR "birth weight" OR "breast feed*" OR breastfeed* OR breastfed OR "breast fed" OR "breech presentation" OR childbirth* OR conception OR contraception* OR contraceptive* OR "early pregnancy loss*" OR "expectant mother*" OR "family planning" OR (fertilization NEAR/1 inhibition) OR "fetal development*" OR "fetal malpresentation" OR "fetal presentation" OR "fetal viability" OR "fetal weight" OR "follicular atresia" OR gestation* OR gravidity OR gynecolog* OR infertility OR "labor presentation" OR lactation OR livebirth OR midwife* OR midwives OR miscarriage* OR "neonatal death*" OR obstetric* OR "ovulation inhibition" OR "ovulation suppression" OR parity OR parturition OR perinatal OR peripartum OR postabortion* OR postpartum OR postnatal OR pregnan* OR puerperium OR sterilization OR stillbirth OR "term birth")	1350443

16	TS=(clitoridectom* OR clitorectom* OR "female circumcision*" OR "female genital cutting" OR "female genital mutilation" OR infibulation)	2260
17	TS=((cervix OR cervical) NEAR/1 (cancer* OR neoplasm*))	74485
18	#17 OR #16 OR #15 OR #14 OR #13 OR #12 OR #11 OR #10 OR #9 OR #8 OR #7 OR #6 OR #5 OR #4 OR #3 OR #2 OR #1	2466031
19	TS=(climate NEAR/2 (adaption* OR associat* OR change* OR changing OR crisis OR induce* OR migrant* OR model$ OR predict* OR refugee* OR resilience OR sensitivity))	367467
20	TS=("air pollution" OR "air pollutants" OR "air quality" OR "carbon footprint$" OR drought* OR "dry corridor*" OR "El Nino" OR "environmental degradation" OR flood$ OR "food insecur*" OR "global environmental change$" OR "global heating" OR "global warming" OR "greenhouse gas*" OR "greenhouse effect$" OR "heavy precipitation" OR heatwave* OR "heat wave*" OR hurricane* OR "La nina" OR landslide* OR "land slide*" OR "lightning strike*" OR megadrought* OR megafire* OR "polar amplification" OR rain$ OR rainfall OR ("sea ice" NEAR/0 shrink*) OR ("sea level" NEAR/0 ris*) OR "sea surface warming" OR storm$ OR typhoon* OR wildfire* OR "wild fire*")	851279
21	TS=((extreme OR destructive OR severe) NEAR/2 (heat OR storm* OR temperature* OR weather$ OR wind$))	35064
22	TS=((destructive OR extreme OR global OR high OR hot OR severe OR warm) NEAR/1 temperature*)	480147
23	TS=("arctic amplification" OR "arctic shrinkage" OR avalanche* OR "brush fire*" OR "carbon offset*" OR "carbon sequestration" OR "carbon sink" OR "carbon sinks" OR cyclon* OR deglaciation OR desertification OR "forest fire*" OR "emissions reduc*" OR "emissions trading" OR "global radiation" OR hurricane* OR "ice mass loss*" OR "Kyoto Protocol" OR mudslide OR "mud slide*" OR "Paris Accord" OR "polar amplification" OR "thermohaline circulation")	152880
24	TS=(chlorofluorocarbon* NEAR/1 (release OR concentration* OR atmosphere*))	65
25	TS=(earth NEAR/1 warming)	167
26	TS=((glacial OR glacier OR "ice cap" OR permafrost OR "polar ice") NEAR/2 (retreat* OR melt*))	6912
27	TS=(ozone NEAR/1 hole)	1298
28	#19 OR #20 OR #21 OR #22 OR #23 OR #24 OR #25 OR #26 OR #27	1623961
29	(TI=(Africa OR Asia OR Caribbean OR "West Indies" OR "South America" OR "Latin America" OR "Central America") OR AB=(Africa OR Asia OR Caribbean OR "West Indies" OR "South America" OR "Latin America" OR "Central America"))	584522
30	TS=(Afghanistan OR Albania* OR Algeria* OR "American Samoa" OR Angola* OR Argentina OR Armenia* OR Azerbaijan OR Bangladesh OR Benin OR Byelarus OR Byelorussian OR Belarus OR Belorussian OR Belorussia OR Belize OR Bhutan OR Bolivia* OR Bosnia* OR Herzegovina OR Hercegovina OR Botswana OR Brazil* OR Brasil* OR Bulgaria* OR "Burkina Faso" OR "Burkina Fasso" OR Burundi OR Urundi OR Cambodia* OR "Khmer Republic" OR Kampuchea OR Cameroon OR Cameroons OR Cameron OR Camerons OR "Cape Verde" OR "Cabo Verde" OR "Central African Republic" OR Chad OR China OR Colombia* OR Comoros OR "Comoro Islands" OR Comores OR Congo OR "Costa Rica" OR "Cote d'Ivoire" OR "Ivory Coast" OR Cuba OR Djibouti OR Dominica OR "Dominican Republic" OR "East Timor" OR "East Timur" OR "Timor Leste" OR Ecuador OR Egypt* OR "El Salvador" OR Eritrea* OR "Equatorial Guinea" OR Eswatini* OR Ethiopia* OR Fiji OR Gabon OR Gambia* OR Gaza OR "Georgia Republic" OR "Georgian Republic" OR Ghana OR Grenada OR Guatemala* OR Guinea OR Guyana OR Haiti OR Honduras OR India OR Indonesia* OR Iran OR Iraq OR Jamaica* OR Jordan* OR Kazakhstan OR Kenya* OR Kiribati OR "Democratic People's Republic	3777344

	of Korea*" OR "North Korea*" OR Kosovo OR "Kyrgyz Republic" OR "Lao PDR" OR Laos OR Lebanon OR Lesotho OR Liberia* OR Libya* OR Macedonia* OR Madagascar OR Malaysia* OR Malaya* OR Malay OR Malawi OR Mali OR Maldives OR "Marshall Islands" OR Mauritania* OR Mauritius OR Mexico OR Mehico OR Micronesia* OR "Middle East" OR Moldova OR Moldovia* OR Moldovian* OR Mongolia OR Montenegro OR Morocco OR Mozambique OR Mocambique OR Myanmar OR Namibia* OR Nauru OR Nepal OR Nicaragua OR Niger OR Nigeria* OR Pakistan* OR Palau OR Palestine* OR Paraguay OR Peru* OR Philippines OR Philipines OR Phillipines OR Phillippines OR Romania* OR Rumania* OR Roumania* OR Russia OR Russian OR Rwanda OR Ruanda OR "Saint Lucia" OR "St Lucia" OR "Saint Vincent" OR "St Vincent" OR Grenadines OR Samoa OR "Samoan Islands" OR "Sao Tome" OR Senegal OR Serbia* OR "Sierra Leone" OR "Spanish Guinea" OR "Sri Lanka" OR Ceylon OR Solomon OR USSR OR "Soviet Union" OR "Union of Soviet Socialist Republics" OR Somalia* OR "South Africa*" OR Sudan OR Suriname OR Surinam OR Swaziland OR Syria OR "Syrian Arab Republic" OR Tajikistan OR Tadzhikistan OR Tadjikistan OR Tadzhik OR Tanzania OR Thailand OR Togo OR "Togolese Republic" OR Tonga OR Tunisia* OR Turkey OR Turkiye OR Turkmenistan OR Tuvalu OR Uganda OR Ukrain* OR Uzbekistan OR Uzbek OR Vanuatu OR Venezuela OR Vietnam OR "Viet Nam" OR "West Bank" OR Yemen OR Zambia OR Zimbabwe)	
31	(TI=((developing OR emerging OR "less* developed" OR "under developed" OR underdeveloped OR "middle income" OR "low* income" OR third-world OR underserved OR "under served" OR deprived OR poor*) NEAR/3 (countr* OR nation$ OR population$ OR world OR economy OR economies)) OR AB=((developing OR emerging OR "less* developed" OR "under developed" OR underdeveloped OR "middle income" OR "low* income" OR third-world OR underserved OR "under served" OR deprived OR poor*) NEAR/2 (countr* OR nation$ OR population$ OR world OR economy OR economies)))	241865
32	(TI=(low* NEAR/1 (countr* OR gdp OR gnp OR "gross domestic" OR "gross national")) OR AB=(low* NEAR/1 (countr* OR gdp OR gnp OR "gross domestic" OR "gross national")))	22138
33	TS=((low* OR middle*) NEAR/4 (countr* OR nation*))	82922
34	(TI=((rural OR remote OR nonmetropolitan OR non-metropolitan OR underserved OR "under served" OR deprived OR shortage) NEAR/0 (communit? OR count? OR area$ OR region$ OR province$ OR district$)) OR AB=((rural OR remote OR nonmetropolitan OR non-metropolitan OR underserved OR "under served" OR deprived OR shortage) NEAR/0 (communit? OR count? OR area$ OR region$ OR province$ OR district$)))	94968
35	TS=("Global South" OR LIC OR LMIC* OR LMICs OR MIC OR South-South OR "rural health*" OR "rural population*")	97125
36	#29 OR #30 OR #31 OR #32 OR #33 OR #34 OR #35	4305946
37	#36 AND #28 AND #18	6549
38	#36 AND #28 AND #18 Timespan: 1994-01-01 to 2023-01-30	6440

4. Cinahl

Interface: Ebsco<br><br>Date of Search: 06 September 2023<br><br>Number of hits: 1694	Field labels<br><br>• MH+ = exploded Cinahl Heading<br>• MH = non exploded Cinahl Heading<br>• TI = title<br>• AB = abstract<br>• Nx = within x words, regardless of order<br>• * = truncation of word for alternate endings

#	Query	Results
S1	(MH "Abortion, Criminal")	326
S2	(MH "Abortion, Induced+")	11,691
S3	(MH "Birth Weight") OR (MH "Infant, Low Birth Weight+")	25,601
S4	(MH "Breast Feeding+")	26,962
S5	(MH "Female Genital Mutilation")	1,747
S6	(MH Contraception+) OR (MH "Contraceptive agents+") OR (MH "Contraceptive devices+")	43,296
S7	(MH "Domestic Violence")	10,308
S8	(MH "Family Characteristics")	7,795
S9	(MH Fertility+)	6,761
S10	(MH "Fetal Development+")	31,216
S11	(MH "Gender-Based Violence")	771
S12	(MH "Gender Equality")	472
S13	(MH "Gender Identity+")	9,243
S14	(MH "Gender Role")	5,549
S15	(MH "Gestational Weight Gain")	432
S16	(MH Parity)	6,347
S17	(MH “Human Immunodeficiency Virus+”) OR (MH "HIV Infections+")	97,577
S18	(MH Infertility+)	13,868
S19	(MH "Intimate Partner Violence")	12,223
S20	(MH "Labor Presentation+")	2,113
S21	(MH Lactation)	4,850
S22	(MH "Maternal Age+") OR (MH "Paternal Age")	12,171
S23	(MH "Maternal-child health") OR (MH “Maternal Health Services+”)	39,257
S24	(MH "Maternal welfare") OR (MH "Maternal Mortality")	7,778
S25	(MH Midwifery)	19,443
S26	(MH "Maternal-Child Care+")	65,089
S27	(MH "Postnatal Period+")	16,510

S28	(MH Pregnancy+) OR (MH "Pregnancy Complications+") OR (MH "Pregnancy Trimesters+")	258,010
S29	(MH " Expectant Parents+")	13,278
S30	(MH "Primary health care") OR (MH "Universal health care")	73,078
S31	(MH "Reproductive Health") OR (MH "Sexual Health")	15,769
S32	(MH "Reproductive Rights")	188
S33	(MH Reproduction) OR (MH "Reproductive Behavior") OR (MH Ejaculation) OR (MH Fertilization+) OR (MH Orgasm) OR (MH Ovulation+) OR (MH "Penile Erection")	21,432
S34	(MH "Self Care")	44,589
S35	(MH "Menstrual cycle+") OR (MH "Sexual behavior+") OR (MH “Sexual Reproduction Periods+”)	90,453
S36	(MH Sexism+)	6,789
S37	(MH "Sex Work")	4,090
S38	(MH "Sexual Dysfunction, Male+") OR (MH “Sexual Dysfunction, Female+”)	10,761
S39	(MH "Sexual Dysfunction, Psychological")	2,006
S40	(MH "Sexually Transmitted Diseases+")	115,032
S41	(MH "Sexual Abuse+")	20,260
S42	(MH "Sex Education")	5,723
S43	(MH "Cervix Neoplasms")	18,371
S44	(MH "Women's Rights")	2,562
S45	((TI AIDS OR AB AIDS) OR (TI chancre OR AB chancre) OR (TI chancroid OR AB chancroid) OR (TI chlamydia OR AB chlamydia) OR (TI "condylomata acuminata" OR AB "condylomata acuminata") OR (TI donovanosis OR AB donovanosis) OR (TI "genital herpes" OR AB "genital herpes") OR (TI "genital tract infection*" OR AB "genital tract infection*") OR (TI "genital wart#" OR AB "genital wart#") OR (TI gonorrhea# OR AB gonorrhea#) OR (TI granuloma OR AB granuloma) OR (TI "great pox" OR AB "great pox") OR (TI "herpes genital*" OR AB "herpes genital*") OR (TI HIV OR AB HIV) OR (TI "reproductive tract infection*" OR AB "reproductive tract infection*") OR (TI "sexually transmitted disease#" OR AB "sexually transmitted disease#") OR (TI "sexually transmitted infection#" OR AB "sexually transmitted infection#") OR (TI syphilis OR AB syphilis) OR (TI "venereal disease#" OR AB "venereal disease#") OR (TI "venereal wart#" OR AB "venereal wart#"))	150,212
S46	((TI adrenarche OR AB adrenarche) OR (TI climacteric* OR AB climacteric*) OR (TI "fertile period*" OR AB "fertile period*") OR (TI "follicular phase" OR AB "follicular phase") OR (TI "luteal phase" OR AB "luteal phase") OR (TI menarche OR AB menarche) OR (TI menopause OR AB menopause) OR (TI menstrua* OR AB menstrua*) OR (TI "ovarian reserve" OR AB "ovarian reserve") OR (TI perimenopause OR AB perimenopause) OR (TI postmenopause OR AB postmenopause) OR (TI premenopause OR AB premenopause) OR (TI puberty OR AB puberty))	29,976
S47	((TI bisexualit* OR AB bisexualit*) OR (TI celibacy OR AB celibacy) OR (TI coitus OR AB coitus) OR (TI "condomless sex" OR AB "condomless sex") OR (TI courtship* OR AB courtship*) OR (TI ejaculation OR AB ejaculation) OR (TI "extramarital relation*" OR AB "extramarital relation*") OR (TI	25,477

	heterosexualit* OR AB heterosexualit*) OR (TI homosexualit* OR AB homosexualit*) OR (TI masturbation OR AB masturbation) OR (TI orgasm OR AB orgasm) OR (TI "penile erection" OR AB "penile erection") OR (TI "protected sex" OR AB "protected sex") OR (TI "responsible sex" OR AB "responsible sex") OR (TI "safe sex" OR AB "safe sex") OR (TI sexism OR AB sexism) OR (TI sexualit* OR AB sexualit*) OR (TI "sex* abstinence" OR AB "sex* abstinence") OR (TI "sexual development*" OR AB "sexual development*") OR (TI "sexual intercourse" OR AB "sexual intercourse") OR (TI "sexual orientation" OR AB "sexual orientation") OR (TI "unprotected intercourse" OR AB "unprotected intercourse") OR (TI "unprotected sex" OR AB "unprotected sex") OR (TI "unsafe sex" OR AB "unsafe sex"))	
S48	((TI dyspareunia OR AB dyspareunia) OR (TI "erectile dysfunction" OR AB "erectile dysfunction") OR (TI "gender dysphoria" OR AB "gender dysphoria") OR (TI "gender disorder*" OR AB "gender disorder*") OR (TI "male impotence" OR AB "male impotence") OR (TI "premature ejaculation*" OR AB "premature ejaculation*") OR (TI "psychosexual disorder*" OR AB "psychosexual disorder*") OR (TI "psychosexual dysfunction*" OR AB "psychosexual dysfunction*") OR (TI "sexual disorder*" OR AB "sexual disorder*") OR (TI "sexual dysfuntion*" OR AB "sexual dysfuntion*") OR (TI "sexual impotence" OR AB "sexual impotence") OR (TI vaginismus OR AB vaginismus) OR (TI "venogenic impotence" OR AB "venogenic impotence"))	6,430
S49	((TI "maternal health service*" OR AB "maternal health service*") OR (TI "self care" OR AB "self care") OR (TI "primary health care" OR AB "primary health care") OR (TI "primary healthcare" OR AB "primary healthcare") OR (TI "universal health care" OR AB "universal health care") OR (TI "universal healthcare" OR AB "universal healthcare"))	39,078
S50	(((TI child OR AB child) OR (TI early OR AB early) OR (TI forced OR AB forced)) W1 (TI marriage OR AB marriage))	734
S51	((TI gender OR AB gender) N2 ((TI equalit* OR AB equalit*) OR (TI equity OR AB equity) OR (TI identit* OR AB identit*) OR (TI norm# OR AB norm#) OR (TI role OR AB role) OR (TI violence OR AB violence)))	11,176
S52	((TI women* OR AB women*) N1 ((TI liberation OR AB liberation) OR (TI right* OR AB right*) OR (TI status OR AB status)))	3,444
S53	(((TI sexual* OR AB sexual*) OR (TI reproductive OR AB reproductive)) N2 ((TI behavio#r* OR AB behavio#r*) OR (TI health OR AB health) OR (TI healthcare OR AB healthcare) OR (TI justice* OR AB justice*) OR (TI right# OR AB right#)))	33,801
S54	(((TI domestic OR AB domestic) OR (TI "intimate partner#" OR AB "intimate partner#") OR (TI sexual* OR AB sexual*) OR (TI spouse# OR AB spouse#) OR (TI women OR AB women)) N3 ((TI abuse OR AB abuse) OR (TI violence OR AB violence)))	30,925
S55	(((TI infant* OR AB infant*) OR (TI matern* OR AB matern*) OR (TI mother* OR AB mother*) OR (TI newborn* OR AB newborn*) OR (TI paternal OR AB paternal) OR (TI perinatal OR AB perinatal) OR (TI prenatal OR AB prenatal) OR (TI "pre natal" OR AB "pre natal")) N3 ((TI age OR AB age) OR (TI death* OR AB death*) OR (TI disease* OR AB disease*) OR (TI injur* OR AB injur*) OR (TI health OR AB health) OR (TI morbidity OR AB morbidity) OR (TI mortality OR AB mortality) OR (TI obesity OR AB obesity) OR (TI welfare OR AB welfare)))	74,899
S56	(((TI fetal OR AB fetal) OR (TI fetus* OR AB fetus*) OR (TI foetus* OR AB foetus*)) N1 ((TI "alcohol effect*" OR AB "alcohol effect*") OR (TI "alcohol spectrum disorder" OR AB "alcohol spectrum disorder") OR (TI "alcohol syndrome" OR AB "alcohol syndrome") OR (TI anoxia OR AB anoxia) OR (TI death* OR AB death*) OR (TI demise OR AB demise) OR (TI disease* OR AB disease*) OR (TI edema OR AB edema) OR (TI "growth restriction" OR AB "growth restriction") OR (TI "growth retardation" OR AB "growth retardation") OR (TI hydrops OR AB hydrops) OR (TI hypoxia OR AB hypoxia) OR (TI malnutrition OR AB malnutrition) OR (TI macrosomia* OR AB macrosomia*) OR (TI "nutrition disorder*" OR AB "nutrition disorder*") OR (TI resorption* OR AB resorption*)))	7,802

S57	((TI sex* OR AB sex*) N1 ((TI education OR AB education) OR (TI harassment* OR AB harassment*) OR (TI offense* OR AB offense*) OR (TI work* OR AB work*)))	14,113
S58	((TI fertility OR AB fertility) N2 ((TI control OR AB control) OR (TI effect* OR AB effect*) OR (TI female# OR AB female#) OR (TI woman OR AB woman) OR (TI women OR AB women) OR (TI outcome# OR AB outcome#)))	2,952
S59	((TI abortion* OR AB abortion*) OR (TI antenatal OR AB antenatal) OR (TI "birth attendant" OR AB "birth attendant") OR (TI "birth control" OR AB "birth control") OR (TI "birth setting*" OR AB "birth setting*") OR (TI “birth outcome” OR AB “birth outome”) OR (TI “birth spacing” OR AB "birth spacing") OR (TI "birth weight" OR AB "birth weight") OR (TI "breast feed*" OR AB "breast feed*") OR (TI “breastfeed*” OR AB “breastfeed*”) OR (TI breastfed OR AB breastfed) OR (TI “breast fed” OR AB "breast fed") OR (TI "breech presentation" OR AB "breech presentation") OR (TI childbirth* OR AB childbirth*) OR (TI conception OR AB conception) OR (TI contraception* OR AB contraception*) OR (TI contraceptive* OR AB contraceptive*) OR (TI "early pregnancy loss*" OR AB "early pregnancy loss*") OR (TI “expectant mother*” OR AB “expectant mother*”) OR (TI “family planning” OR AB "family planning") OR ((TI fertilization OR AB fertilization) N2 (TI inhibition OR AB inhibition)) OR (TI "fetal development*" OR AB "fetal development*") OR (TI “fetal malpresentation” OR AB "fetal malpresentation") OR (TI "fetal presentation" OR AB "fetal presentation") OR (TI "fetal viability" OR AB "fetal viability") OR (TI "fetal weight" OR AB "fetal weight") OR (TI "follicular atresia" OR AB "follicular atresia") OR (TI gestation* OR AB gestation*) OR (TI gravidity OR AB gravidity) OR (TI gynecolog* OR AB gynecolog*) OR (TI infertility OR AB infertility) OR (TI "labor presentation" OR AB "labor presentation") OR (TI lactation OR AB lactation) OR (TI livebirth OR AB livebirth) OR (TI midwife* OR AB midwife*) OR (TI midwives OR AB midwives) OR (TI miscarriage* OR AB miscarriage*) OR (TI "neonatal death*" OR AB "neonatal death*") OR (TI obstetric* OR AB obstetric*) OR (TI "ovulation inhibition" OR AB "ovulation inhibition") OR (TI "ovulation suppression" OR AB "ovulation suppression") OR (TI parity OR AB parity) OR (TI parturition OR AB parturition) OR (TI perinatal OR AB perinatal) OR (TI peripartum OR AB peripartum) OR (TI postabortion* OR AB postabortion*) OR (TI postpartum OR AB postpartum) OR (TI postnatal OR AB postnatal) OR (TI pregnan* OR AB pregnan*) OR (TI puerperium OR AB puerperium) OR (TI sterilization OR AB sterilization) OR (TI stillbirth OR AB stillbirth) OR (TI "term birth" OR AB "term birth"))	371,298
S60	((TI clitoridectom* OR AB clitoridectom*) OR (TI clitorectom* OR AB clitorectom*) OR (TI "female circumcision*" OR AB "female circumcision*") OR (TI “female genital cutting” OR AB "female genital cutting ") OR (TI "female genital mutilation" OR AB "female genital mutilation") OR (TI infibulation OR AB infibulation))	1,386
S61	(((TI cervix OR AB cervix) OR (TI cervical OR AB cervical)) N1 ((TI cancer* OR AB cancer*) OR (TI neoplasm* OR AB neoplasm*)))	16,076
S62	S1 OR S2 OR S3 OR S4 OR S5 OR S6 OR S7 OR S8 OR S9 OR S10 OR S11 OR S12 OR S13 OR S14 OR S15 OR S16 OR S17 OR S18 OR S19 OR S20 OR S21 OR S22 OR S23 OR S24 OR S25 OR S26 OR S27 OR S28 OR S29 OR S30 OR S31 OR S32 OR S33 OR S34 OR S35 OR S36 OR S37 OR S38 OR S39 OR S40 OR S41 OR S42 OR S43 OR S44 OR S45 OR S46 OR S47 OR S48 OR S49 OR S50 OR S51 OR S52 OR S53 OR S54 OR S55 OR S56 OR S57 OR S58 OR S59 OR S60 OR S61	973,757
S63	(MH "Air Pollutants+")	5,521
S64	(MH "Air Pollution")	6,837
S65	(MH "Climate Change+")	2,837
S66	(MH "Greenhouse Effect")	1,400
S67	(MH "Greenhouse Gases")	182

S68	(MH "Carbon Footprint")	200
S69	(MH "Extreme Weather")	33
S70	(MH "Food security")	4,516
S71	(MH Rain)	415
S72	(MH Wildfires)	220
S73	((TI climate OR AB climate) N2 ((TI adaption* OR AB adaption*) OR (TI associat* OR AB associat*) OR (TI change* OR AB change*) OR (TI changing OR AB changing) OR (TI crisis OR AB crisis) OR (TI induce* OR AB induce*) OR (TI migrant* OR AB migrant*) OR (TI model# OR AB model#) OR (TI predict* OR AB predict*) OR (TI refugee* OR AB refugee*) OR (TI resilience OR AB resilience) OR (TI sensitivity OR AB sensitivity)))	6,893
S74	(TI “arctic amplification” OR AB "arctic amplification") OR (TI "arctic shrinkage" OR AB "arctic shrinkage") OR (TI avalanche* OR AB avalanche*) OR (TI "brush fire*" OR AB "brush fire*") OR (TI “carbon offset* OR AB “carbon offset*”) OR (TI “carbon sequestration” OR AB "carbon sequestration") OR (TI "carbon sink" OR AB "carbon sink") OR (TI "carbon sinks" OR AB "carbon sinks") OR (TI cyclon* OR AB cyclon*) OR (TI deglaciation OR AB deglaciation) OR (TI desertification OR AB desertification) OR (TI "forest fire*" OR AB "forest fire*") OR (TI “emissions reduc*” OR AB “emissions reduc*”) OR (TI “emissions trading” OR AB "emissions trading") OR (TI "global radiation" OR AB "global radiation") OR (TI hurricane* OR AB hurricane*) OR (TI "ice mass loss*" OR AB "ice mass loss*") OR (TI “Kyoto Protocol” OR AB "Kyoto Protocol") OR (TI mudslide OR AB mudslide) OR (TI "mud slide*" OR AB "mud slide*”) OR (TI “Paris accord” OR AB "Paris Accord") OR (TI "thermohaline circulation" OR AB "thermohaline circulation"))	270
S75	(((TI destructive OR AB destructive) OR (TI extreme OR AB extreme) OR (TI global OR AB global) OR (TI high OR AB high) OR (TI hot OR AB hot) OR (TI severe OR AB severe) OR (TI warm OR AB warm)) N2 (TI temperature* OR AB temperature*))	2,533
S76	(((TI extreme OR AB extreme) OR (TI destructive OR AB destructive) OR (TI severe OR AB severe)) N3 ((TI heat OR AB heat) OR (TI storm* OR AB storm*) OR (TI temperature* OR AB temperature*) OR (TI weather# OR AB weather#) OR (TI wind# OR AB wind#)))	1,435
S77	((TI "air pollution" OR AB "air pollution") OR (TI "air pollutants" OR AB "air pollutants") OR (TI "air quality" OR AB "air quality") OR (TI "carbon footprint#" OR AB "carbon footprint#") OR (TI drought* OR AB drought*) OR (TI "dry corridor*" OR AB "dry corridor*") OR (TI “El Nino” OR AB "El Nino") OR (TI "environmental degradation" OR AB "environmental degradation") OR (TI flood# OR AB flood#) OR (TI "food insecur*" OR AB "food insecur*") OR (TI "global environmental change#" OR AB "global environmental change#") OR (TI “global heating” OR AB "global heating") OR (TI "global warming" OR AB "global warming") OR (TI "greenhouse gas*" OR AB "greenhouse gas*") OR (TI "greenhouse effect#" OR AB "greenhouse effect#") OR (TI “heavy precipitation” OR AB "heavy precipitation") OR (TI heatwave* OR AB heatwave*) OR (TI "heat wave*" OR AB "heat wave*") OR (TI “La nina” OR AB "La nina") OR (TI landslide* OR AB landslide*) OR (TI "land slide*" OR AB "land slide*") OR (TI lightning OR AB lightning) OR (TI megadrought* OR AB megadrought*) OR (TI megafire* OR AB megafire*) OR (TI “polar amplification” OR AB "polar amplification") OR (TI rain# OR AB rain#) OR (TI rainfall OR AB rainfall) OR ((TI "sea ice" OR AB "sea ice") N1 (TI shrink* OR AB shrink*)) OR ((TI "sea level" OR AB "sea level") N1 (TI ris* OR AB ris*)) OR (TI "sea surface warming" OR AB "sea surface warming") OR (TI storm# OR AB storm#) OR (TI typhoon* OR AB typhoon*) OR (TI wildfire* OR AB wildfire*) OR (TI "wild fire*" OR AB "wild fire*")	24,587
S78	((TI chlorofluorocarbon* OR AB chlorofluorocarbon*) N2 ((TI release OR AB release) OR (TI concentration* OR AB concentration*) OR (TI atmosphere* OR AB atmosphere*)))	0

S79	((TI earth OR AB earth) N1 (TI warming OR AB warming))	10
S80	(((TI glacial OR AB glacial) OR (TI glacier OR AB glacier) OR (TI "ice cap" OR AB "ice cap") OR (TI permafrost OR AB permafrost) OR (TI "polar ice" OR AB "polar ice")) N2 ((TI retreat* OR AB retreat*) OR (TI melt* OR AB melt*)))	44
S81	((TI ozone OR AB ozone) N2 (TI hole OR AB hole))	13
S82	S63 OR S64 OR S65 OR S66 OR S67 OR S68 OR S69 OR S70 OR S71 OR S72 OR S73 OR S74 OR S75 OR S76 OR S77 OR S78 OR S79 OR S80 OR S81	41,749
S83	(MH "Medically Underserved Area") OR (MH "Developing Countries") OR (MH "Rural Health") OR (MH "Rural Population")	41,461
S84	(MH Afghanistan) OR (MH Albania) OR (MH Algeria) OR (MH Angola) OR (MH Argentina) OR (MH Armenia) OR (MH Azerbaijan) OR (MH Bangladesh) OR (MH Benin) OR (MH "Byelarus") OR (MH Belize) OR (MH Bhutan) OR (MH Bolivia) OR (MH "Bosnia-Herzegovina") OR (MH Botswana) OR (MH Brazil) OR (MH Bulgaria) OR (MH "Burkina Faso") OR (MH Burundi) OR (MH Cambodia) OR (MH Cameroon) OR (MH "Cape Verde") OR (MH "Central African Republic") OR (MH Chad) OR (MH China+) OR (MH Colombia) OR (MH Congo) OR (MH "Costa Rica") OR (MH "Cote d'Ivoire") OR (MH Cuba) OR (MH Djibouti) OR (MH "Dominican Republic") OR (MH Ecuador) OR (MH Egypt) OR (MH "El Salvador") OR (MH Eritrea) OR (MH "Equatorial Guinea") OR (MH Ethiopia) OR (MH Gabon) OR (MH Gambia) OR (MH Georgia) OR (MH Ghana) OR (MH Guatemala) OR (MH Guinea) OR (MH Guinea-Bissau) OR (MH Guyana) OR (MH Haiti) OR (MH Honduras) OR (MH India) OR (MH Indonesia) OR (MH Iran) OR (MH Iraq) OR (MH Jamaica) OR (MH Jordan) OR (MH Kazakhstan) OR (MH Kenya) OR (MH "North Korea") OR (MH Yugoslavia) OR (MH Kyrgyzstan) OR (MH Laos) OR (MH Lebanon) OR (MH Lesotho) OR (MH Liberia) OR (MH Libya) OR (MH “Macedonia (Republic)”) OR (MH Madagascar) OR (MH Malaysia) OR (MH Malawi) OR (MH Mali) OR (MH Mauritania) OR (MH Micronesia) OR (MH “Indian Ocean Islands”) OR (MH Mexico) OR (MH Moldova) OR (MH Mongolia) OR (MH Morocco) OR (MH Mozambique) OR (MH Myanmar) OR (MH Namibia) OR (MH Nepal) OR (MH Nicaragua) OR (MH Niger) OR (MH Nigeria) OR (MH Pakistan) OR (MH "Papua New Guinea") OR (MH Paraguay) OR (MH Peru) OR (MH Philippines) OR (MH Romania) OR (MH Russia) OR (MH Rwanda) OR (MH "Samoa+") OR (MH Senegal) OR (MH Serbia) OR (MH "Sierra Leone") OR (MH Melanesia+) OR (MH "Sri Lanka") OR (MH Somalia) OR (MH Sudan) OR (MH "South Africa") OR (MH Suriname) OR (MH Swaziland) OR (MH Syria) OR (MH Tajikistan) OR (MH Tanzania) OR (MH Timor) OR (MH “East Timor”) OR (MH Thailand) OR (MH Togo) OR (MH Polynesia) OR (MH Tunisia) OR (MH Turkey) OR (MH Turkmenistan) OR (MH Uganda) OR (MH Ukraine) OR (MH USSR+) OR (MH Uzbekistan) OR (MH Venezuela) OR (MH Vietnam) OR (MH Yemen) OR (MH Zambia) OR (MH Zimbabwe)	405,247
S85	((TI Africa OR AB Africa) OR (TI Asia OR AB Asia) OR (TI Caribbean OR AB Caribbean) OR (TI "West Indies" OR AB "West Indies") OR (TI "South America" OR AB "South America") OR (TI "Latin America" OR AB "Latin America") OR (TI "Central America" OR AB "Central America"))	58,148
S86	((TI Afghanistan OR AB Afghanistan) OR (TI Albania* OR AB Albania*) OR (TI Algeria* OR AB Algeria*) OR (TI "American Samoa" OR AB "American Samoa") OR (TI Angola* OR AB Angola*) OR (TI Argentina OR AB Argentina) OR (TI Armenia* OR AB Armenia*) OR (TI Azerbaijan OR AB Azerbaijan) OR (TI Bangladesh OR AB Bangladesh) OR (TI Benin OR AB Benin) OR (TI Byelarus OR AB Byelarus) OR (TI Byelorussian OR AB Byelorussian) OR (TI Belarus OR AB Belarus) OR (TI Belorussian OR AB Belorussian) OR (TI Belorussia OR AB Belorussia) OR (TI Belize OR AB Belize) OR (TI Bhutan OR AB Bhutan) OR (TI Bolivia* OR AB Bolivia*) OR (TI Bosnia* OR AB Bosnia*) OR (TI Herzegovina OR AB Herzegovina) OR (TI Hercegovina OR AB Hercegovina) OR (TI Botswana OR AB Botswana) OR (TI Brazil* OR AB Brazil*) OR (TI Brasil* OR AB Brasil*) OR (TI Bulgaria* OR AB Bulgaria*) OR (TI "Burkina Faso" OR AB "Burkina Faso") OR (TI "Burkina Fasso"	341,290

	OR AB "Burkina Fasso") OR (TI Burundi OR AB Burundi) OR (TI Urundi OR AB Urundi) OR (TI Cambodia* OR AB Cambodia*) OR (TI "Khmer Republic" OR AB "Khmer Republic") OR (TI Kampuchea OR AB Kampuchea) OR (TI Cameroon OR AB Cameroon) OR (TI Cameroons OR AB Cameroons) OR (TI Cameron OR AB Cameron) OR (TI Camerons OR AB Camerons) OR (TI "Cape Verde" OR AB "Cape Verde") OR (TI "Cabo Verde" OR AB "Cabo Verde") OR (TI "Central African Republic" OR AB "Central African Republic") OR (TI Chad OR AB Chad) OR (TI China OR AB China) OR (TI Colombia* OR AB Colombia*) OR (TI Comoros OR AB Comoros) OR (TI "Comoro Islands" OR AB "Comoro Islands") OR (TI Comores OR AB Comores) OR (TI Congo OR AB Congo) OR (TI "Costa Rica" OR AB "Costa Rica") OR (TI "Cote d'Ivoire" OR AB "Cote d'Ivoire") OR (TI "Ivory Coast" OR AB "Ivory Coast") OR (TI Cuba OR AB Cuba) OR (TI Djibouti OR AB Djibouti) OR (TI Dominica OR AB Dominica) OR (TI "Dominican Republic" OR AB "Dominican Republic") OR (TI "East Timor" OR AB "East Timor") OR (TI "East Timur" OR AB "East Timur") OR (TI "Timor Leste" OR AB "Timor Leste") OR (TI Ecuador OR AB Ecuador) OR (TI Egypt* OR AB Egypt*) OR (TI "El Salvador" OR AB "El Salvador") OR (TI Eritrea* OR AB Eritrea*) OR (TI "Equatorial Guinea" OR AB "Equatorial Guinea") OR (TI Eswatini* OR AB Eswatini*) OR (TI Ethiopia* OR AB Ethiopia*) OR (TI Fiji OR AB Fiji) OR (TI Gabon OR AB Gabon) OR (TI Gambia* OR AB Gambia*) OR (TI Gaza OR AB Gaza) OR (TI "Georgia Republic" OR AB "Georgia Republic") OR (TI "Georgian Republic" OR AB "Georgian Republic") OR (TI Ghana OR AB Ghana) OR (TI Grenada OR AB Grenada) OR (TI Guatemala* OR AB Guatemala*) OR (TI Guinea OR AB Guinea) OR (TI Guyana OR AB Guyana) OR (TI Haiti OR AB Haiti) OR (TI Honduras OR AB Honduras) OR (TI India OR AB India) OR (TI Indonesia* OR AB Indonesia*) OR (TI Iran OR AB Iran) OR (TI Iraq OR AB Iraq) OR (TI Jamaica* OR AB Jamaica*) OR (TI Jordan* OR AB Jordan*) OR (TI Kazakhstan OR AB Kazakhstan) OR (TI Kenya* OR AB Kenya*) OR (TI Kiribati OR AB Kiribati) OR (TI "Democratic People's Republic of Korea*" OR AB "Democratic People's Republic of Korea*") OR (TI "North Korea*" OR AB "North Korea*") OR (TI Kosovo OR AB Kosovo) OR (TI "Kyrgyz Republic" OR AB "Kyrgyz Republic") OR (TI "Lao PDR" OR AB "Lao PDR") OR (TI Laos OR AB Laos) OR (TI Lebanon OR AB Lebanon) OR (TI Lesotho OR AB Lesotho) OR (TI Liberia* OR AB Liberia*) OR (TI Libya* OR AB Libya*) OR (TI Macedonia* OR AB Macedonia*) OR (TI Madagascar OR AB Madagascar) OR (TI Malaysia* OR AB Malaysia*) OR (TI Malaya* OR AB Malaya*) OR (TI Malay OR AB Malay) OR (TI Malawi OR AB Malawi) OR (TI Mali OR AB Mali) OR (TI Maldives OR AB Maldives) OR (TI "Marshall Islands" OR AB "Marshall Islands") OR (TI Mauritania* OR AB Mauritania*) OR (TI Mauritius OR AB Mauritius) OR (TI Mexico OR AB Mexico) OR (TI Mehico OR AB Mehico) OR (TI Micronesia* OR AB Micronesia*) OR (TI "Middle East" OR AB "Middle East") OR (TI Moldova OR AB Moldova) OR (TI Moldovia* OR AB Moldovia*) OR (TI Moldovian* OR AB Moldovian*) OR (TI Mongolia OR AB Mongolia) OR (TI Montenegro OR AB Montenegro) OR (TI Morocco OR AB Morocco) OR (TI Mozambique OR AB Mozambique) OR (TI Mocambique OR AB Mocambique) OR (TI Myanmar OR AB Myanmar) OR (TI Namibia* OR AB Namibia*) OR (TI Nauru OR AB Nauru) OR (TI Nepal OR AB Nepal) OR (TI Nicaragua OR AB Nicaragua) OR (TI Niger OR AB Niger) OR (TI Nigeria* OR AB Nigeria*) OR (TI Pakistan* OR AB Pakistan*) OR (TI Palau OR AB Palau) OR (TI Palestine* OR AB Palestine*) OR (TI Paraguay OR AB Paraguay) OR (TI Peru* OR AB Peru*) OR (TI Philippines OR AB Philippines) OR (TI Philipines OR AB Philipines) OR (TI Phillipines OR AB Phillipines) OR (TI Phillippines OR AB Phillippines) OR (TI Romania* OR AB Romania*) OR (TI Rumania* OR AB Rumania*) OR (TI Roumania* OR AB Roumania*) OR (TI Russia OR AB Russia) OR (TI Russian OR AB Russian) OR (TI Rwanda OR AB Rwanda) OR (TI Ruanda OR AB Ruanda) OR (TI "Saint Lucia" OR AB "Saint Lucia") OR (TI "St Lucia" OR AB "St Lucia") OR (TI "Saint Vincent" OR AB "Saint Vincent") OR (TI "St Vincent" OR AB "St Vincent") OR (TI Grenadines OR AB Grenadines) OR (TI Samoa OR AB Samoa) OR (TI "Samoan Islands" OR AB "Samoan Islands") OR (TI "Sao Tome" OR AB "Sao Tome") OR (TI Senegal OR AB Senegal) OR (TI Serbia* OR AB Serbia*) OR (TI "Sierra Leone" OR AB "Sierra Leone") OR (TI "Spanish Guinea" OR AB "Spanish Guinea") OR (TI "Sri Lanka" OR AB "Sri Lanka") OR (TI Ceylon OR AB Ceylon) OR (TI Solomon OR AB Solomon) OR		

	(TI USSR OR AB USSR) OR (TI "Soviet Union" OR AB "Soviet Union") OR (TI "Union of Soviet Socialist Republics" OR AB "Union of Soviet Socialist Republics") OR (TI Somalia* OR AB Somalia*) OR (TI "South Africa*" OR AB "South Africa*") OR (TI Sudan OR AB Sudan) OR (TI Suriname OR AB Suriname) OR (TI Surinam OR AB Surinam) OR (TI Swaziland OR AB Swaziland) OR (TI Syria OR AB Syria) OR (TI "Syrian Arab Republic" OR AB "Syrian Arab Republic") OR (TI Tajikistan OR AB Tajikistan) OR (TI Tadzhikistan OR AB Tadzhikistan) OR (TI Tadjikistan OR AB Tadjikistan) OR (TI Tadzhik OR AB Tadzhik) OR (TI Tanzania OR AB Tanzania) OR (TI Thailand OR AB Thailand) OR (TI Togo OR AB Togo) OR (TI "Togolese Republic" OR AB "Togolese Republic") OR (TI Tonga OR AB Tonga) OR (TI Tunisia* OR AB Tunisia*) OR (TI Turkey OR AB Turkey) OR (TI Turkiye OR AB Turkiye) OR (TI Turkmenistan OR AB Turkmenistan) OR (TI Tuvalu OR AB Tuvalu) OR (TI Uganda OR AB Uganda) OR (TI Ukrain* OR AB Ukrain*) OR (TI Uzbekistan OR AB Uzbekistan) OR (TI Uzbek OR AB Uzbek) OR (TI Vanuatu OR AB Vanuatu) OR (TI Venezuela OR AB Venezuela) OR (TI Vietnam OR AB Vietnam) OR (TI "Viet Nam" OR AB "Viet Nam") OR (TI "West Bank" OR AB "West Bank") OR (TI Yemen OR AB Yemen) OR (TI Zambia OR AB Zambia) OR (TI Zimbabwe OR AB Zimbabwe))	
S87	(((TI developing OR AB developing) OR (TI emerging OR AB emerging) OR (TI "less* developed" OR AB "less* developed") OR (TI "under developed" OR AB "under developed") OR (TI underdeveloped OR AB underdeveloped) OR (TI "middle income" OR AB "middle income") OR (TI "low* income" OR AB "low* income") OR (TI third-world OR AB third-world) OR (TI underserved OR AB underserved) OR (TI "under served" OR AB "under served") OR (TI deprived OR AB deprived) OR (TI poor* OR AB poor*)) N1 ((TI countr* OR AB countr*) OR (TI nation# OR AB nation#) OR (TI population# OR AB population#) OR (TI world OR AB world) OR (TI economy OR AB economy) OR (TI economies OR AB economies)))	44,171
S88	((TI low* OR AB low*) N1 ((TI countr* OR AB countr*) OR (TI gdp OR AB gdp) OR (TI gnp OR AB gnp) OR (TI "gross domestic" OR AB "gross domestic") OR (TI "gross national" OR AB "gross national")))	5,699
S89	(((TI low* OR AB low*) OR (TI middle* OR AB middle*)) N4 ((TI countr* OR AB countr*) OR (TI nation* OR AB nation*)))	23,698
S90	(((TI rural OR AB rural) OR (TI remote OR AB remote) OR (TI nonmetropolitan OR AB nonmetropolitan) OR (TI non-metropolitan OR AB non-metropolitan) OR (TI underserved OR AB underserved) OR (TI "under served" OR AB "under served") OR (TI deprived OR AB deprived) OR (TI shortage OR AB shortage)) W1 ((TI communit? OR AB communit?) OR (TI count? OR AB count?) OR (TI area# OR AB area#) OR (TI region# OR AB region#) OR (TI province# OR AB province#) OR (TI district# OR AB district#)))	23,726
S91	((TI "Global South" OR AB "Global South") OR (TI LIC OR AB LIC) OR (TI LMIC* OR AB LMIC*) OR (TI LMICs OR AB LMICs) OR (TI MIC OR AB MIC) OR (TI South-South OR AB South-South) OR (TI "rural health*" OR AB "rural health*") OR (TI "rural population* OR AB "rural population*"))	13,241
S92	S83 OR S84 OR S85 OR S86 OR S87 OR S88 OR S89 OR S90 OR S91	592,893
S93	S62 AND S82 AND S92	1,618
S94	S62 AND S82 AND S92	1,615

5. Google Scholar

Interface: Google Scholar web interface<br>scholar.google.com<br><br>Date of Search: 06 September 2023<br><br>Number of hits: First 100	Field labels<br><br>• | = OR
"climate change" OR "air pollution" OR "global warming" OR "sea level rise" OR "food insecurity" AND pregnancy OR contraception OR "reproductive health" OR sexual OR infant OR mother OR maternal AND low income OR middle income OR developing countries<br><br>Publications from 1994-2023<br><br>Note: patent and citations excluded	

6. WHO Global Index Medicus

Interface: Global Index Medicus web interface<br>https://www.globalindexmedicus.net/<br><br>Date of Search: 06 Septempber 2023<br><br>Number of hits: 218	Field labels<br><br>• Tw = Title, Abstract, Subject
(("climate change" OR "global warming" OR "air pollution" OR "food insecurity") AND ("pregnancy" OR "Reproductive Health" OR "Sexual Health" OR "Contraception" OR "Maternal Health" OR "Infant Health" OR "Reproductive Behavior" OR "Sexual behavior")) AND (year_cluster:[1994 TO 2022])	

PRISMA-ScR Checklist

SECTION	ITEM	PRISMA-ScR CHECKLIST ITEM	REPORTED ON PAGE #
**TITLE**			
Title	1	Identify the report as a scoping review.	1
**ABSTRACT**			
Structured summary	2	Provide a structured summary that includes (as applicable): background, objectives, eligibility criteria, sources of evidence, charting methods, results, and conclusions that relate to the review questions and objectives.	2
**INTRODUCTION**			
Rationale	3	Describe the rationale for the review in the context of what is already known. Explain why the review questions/objectives lend themselves to a scoping review approach.	3-4
Objectives	4	Provide an explicit statement of the questions and objectives being addressed with reference to their key elements (e.g., population or participants, concepts, and context) or other relevant key elements used to conceptualize the review questions and/or objectives.	4
**METHODS**			
Protocol and registration	5	Indicate whether a review protocol exists; state if and where it can be accessed (e.g., a Web address); and if available, provide registration information, including the registration number.	4
Eligibility criteria	6	Specify characteristics of the sources of evidence used as eligibility criteria (e.g., years considered, language, and publication status), and provide a rationale.	5-7
Information sources*	7	Describe all information sources in the search (e.g., databases with dates of coverage and contact with authors to identify additional	4-5

		sources), as well as the date the most recent search was executed.	
Search	8	Present the full electronic search strategy for at least 1 database, including any limits used, such that it could be repeated.	4-5
Selection of sources of evidence†	9	State the process for selecting sources of evidence (i.e., screening and eligibility) included in the scoping review.	6
Data charting process‡	10	Describe the methods of charting data from the included sources of evidence (e.g., calibrated forms or forms that have been tested by the team before their use, and whether data charting was done independently or in duplicate) and any processes for obtaining and confirming data from investigators.	7
Data items	11	List and define all variables for which data were sought and any assumptions and simplifications made.	7
Critical appraisal of individual sources of evidence§	12	If done, provide a rationale for conducting a critical appraisal of included sources of evidence; describe the methods used and how this information was used in any data synthesis (if appropriate).	NA
Synthesis of results	13	Describe the methods of handling and summarizing the data that were charted.	7
**RESULTS**			
Selection of sources of evidence	14	Give numbers of sources of evidence screened, assessed for eligibility, and included in the review, with reasons for exclusions at each stage, ideally using a flow diagram.	8
Characteristics of sources of evidence	15	For each source of evidence, present characteristics for which data were charted and provide the citations.	7-9
Critical appraisal within sources of evidence	16	If done, present data on critical appraisal of included sources of evidence (see item 12).	NA
Results of individual sources of evidence	17	For each included source of evidence, present the relevant data that were charted that relate to the review questions and objectives.	9
Synthesis of results	18	Summarize and/or present the charting results as they relate to the review questions and objectives.	9-11
**DISCUSSION**			
Summary of evidence	19	Summarize the main results (including an overview of concepts, themes, and types of evidence available), link to the review questions and objectives, and consider the relevance to key groups.	12-14

Limitations	20	Discuss the limitations of the scoping review process.	14
Conclusions	21	Provide a general interpretation of the results with respect to the review questions and objectives, as well as potential implications and/or next steps.	15
**FUNDING**			
Funding	22	Describe sources of funding for the included sources of evidence, as well as sources of funding for the scoping review. Describe the role of the funders of the scoping review.	15-16

JBI = Joanna Briggs Institute; PRISMA-ScR = Preferred Reporting Items for Systematic reviews and Meta-Analyses extension for Scoping Reviews.

Table A. Characteristics of included articles, presented by SRHR domains (secondarily by climate factor).

Author	Title	Year	Country (income level) [n=# of study countries]	Study Setting	Study Design	Sample and data source [n= # of participants]	Climate data sources (non-exhaustive)	Climate change factors	Key findings
**MATERNAL AND NEWBORN HEALTH (n=37)**									
Grace et al.	Exploring strategies for investigating the mechanisms linking climate and individual-level child health outcomes: An analysis of birth weight in Mali	2021	Mali (low)	Mixed	Quantitative	Newborns included in the Mali DHS in 2000, 2006, and 2012 [sample size not found]	CHIRPS dataset, Princeton University's Terrestrial Hydrology Research group, Normalized Difference Vegetation Index from the Integrated Climate Data Center at University of Hamburg	Extreme temperature (increasing/extreme heat)	-High temperatures and low levels of agricultural production are consistently associated with lower birth weights, and exposure to malarious conditions may increase likelihood of stillbirths.<br>-An infant is likely to have lower birth weight when exposed to more days over 100°F during the third trimester ($p < .01$) and first trimester ($p = .06$).
Guo et al.	Association of daytime-only, nighttime-only, and compound heat waves with preterm birth by urban-rural area and	2023	China (upper middle)	Mixed	Quantitative	Singleton newborns delivered in the warm seasons from April to October registered in the National Maternal Near Miss Surveillance System between 2012 and 2019 [n=5 446 088]	National Climate Center of China Meteorological Administration, ECMWF	Extreme temperature (increasing/extreme heat)	- Exposure to heat waves in rural areas was associated with a higher risk for preterm birth (PTB) than in urban areas, except for compound heat waves in higher indexes.<br>- Pregnant women were at a 1.6% to 3.7% higher risk for PTB when exposed to

	regional socioeconomic status in China								compound heat waves during the last week before delivery and a 2.7% to 4.2% higher risk when exposed to daytime only heat waves, compared with those who were unexposed.
He et al.	Association of maternal exposure to compound hot extreme during pregnancy with preterm birth and the potential biological mechanisms in Guangzhou	2022	China (upper middle)	Urban	Quantitative	Pregnant women who had a premature and full-term birth in the warm season (May-October) from 2014 to 2017 who were apart of the Prenatal Environment and Offspring Health Prospective Birth Cohort [n=311]	China Meteorological Science Data Sharing Service Platform	Extreme temperature (increasing/extreme heat)	-Pregnant women exposed to day-night composite fever during pregnancy may increase the risk of premature birth.<br>-Day-night composite fever is positively correlated with maternal serum CRP, ET-1 and MDA levels.<br>-These biomarkers are positively correlated with preterm birth.
Le et al.	The impacts of temperature shocks on birth weight in Vietnam	2021	Vietnam (lower middle)	Rural	Quantitative	Newborns born during the period of 2004-2011 reported in the Vietnam Multiple Indicator Cluster Survey (waves 3 and 4) [n=1961]	Meteorological and Hydrological Administration for Vietnam's temperature records	Extreme temperature (increasing/extreme heat)	-One standard deviation increase in temperature relative to the local norm during the first trimester of pregnancy reduces the child's weight at birth by 2.2 percent or 67 g.<br>-The impacts of rising temperature started to kick in around 28°C during the first trimester.
Li et al.	Maternal exposure to extreme high-temperature, particulate air pollution and macrosomia in 14 countries of Africa	2023	African continent [n=14] (See article for more details)	Mixed	Quantitative	Newborns with complete birth records recorded in the DHS from 2015-2020 across each study country [n=106382]	NOAA Physical Sciences Laboratory, Climate Prediction Center of the National Center for Environmental Prediction	Extreme temperatures (increasing/extreme temperature)	-There is a significant positive association between extreme-high temperature (EHT), PM2.5 and macrosomia.<br>-EHT had a higher effect on macrosomia in middle/later stages of pregnancy.<br>-Exposure to EHT during pregnancy was significantly associated with a risk of macrosomia compared with non-exposure maternal, with the largest RR in Eastern Africa.
Liu et al.	Same environment, stratified impacts? Air pollution, extreme temperatures,	2022	China (upper middle)	Urban	Quantitative	Newborns reported in the registry of birth certificates in one district in Guangzhou from 2009-2011 [n=53879]	Universal thermal climate indices from Copernicus and the ECMWF, NOAA	Extreme temperatures (increasing/extreme heat)	-The negative association between ambient exposures and birth weight is twice as large at lower conditional quantiles of birth weights as at the median.<br>-The protection associated with college-educated mothers with

	and birth weight in South China								respect to pollution and extreme heat is heterogeneous and potentially substantial: between 0.02 and 0.34 standard deviations of birth weights, depending on the conditional quantiles.
McElroy et al.	Extreme heat, preterm birth, and stillbirth: A global analysis across 14 lower-middle income countries	2021	LMICs across regions [n=14] (See article for details)	Mixed	Quantitative	Newborns as reported by women aged 15–49 who responded to DHS surveys (phase 7) in 14 LMICs conducted between 2014-2018 [n=103535]	Climate Prediction Center's Global Daily Temperature data	Extreme temperatures (increasing/extreme heat)	-Higher risk of preterm and stillbirth among women who were exposed to extreme heat within the seven days before giving birth or experienced diurnal temperature ranges (i.e., difference between daily maximum and minimum temperatures) of less than 16 ◦C
Nyadanu et al.	Prenatal exposure to long-term heat stress and stillbirth in Ghana: A within-space time-series analysis	2023	Ghana (lower middle)	Mixed	Quantitative	Mothers who had a stillbirth recorded by the Centre for Health Information Management of the Ghana Health Service from 2012-2020 [n=90532]	ECMWF	Extreme temperatures (increasing/extreme heat)	-Long-term exposure to moderate heat stress showed a higher risk of stillbirth.<br>-There are possible effects of heat stress during the preconception period.<br>-The risk was slightly greater during the dry and dusty winter season (harmattan) than during the wet rainy summer season.<br>-Districts with low population density, low gross domestic product, and low air pollution which collectively defined rural districts were at higher risk as compared to those in the high level (urban districts).
Qiu et al.	Extreme temperature exposure increases the risk of preterm birth in women with abnormal pre-pregnancy body mass index: A cohort study in a southern province of China	2023	China (upper middle)	Urban	Quantitative	Women with abnormal pre-pregnancy body mass index who participated in the National Free Preconception Health Examination Project and had successful pregnancies in the Guangdong Province from 2014-2017 [n=251257]	National Weather Data Sharing System	Extreme temperatures (increasing/extreme heat)	-The risk of preterm birth (PTB) rose with extreme temperature exposure during the third trimester but decreased with cold exposure in the first and two trimesters.<br>-The association with low temperature was stronger for underweight women, but high temperature was stronger for obese or overweight women.

Ren et al.	Exploration of the preterm birth risk-related heat event thresholds for pregnant women: A population-based cohort study in China	2023	China (upper middle)	Mixed	Quantitative	Singleton newborns recorded in the National Maternal and Newborn Health Monitoring Project from 2014-2018 [n=210798]	China Meteorological Data Service Center	Extreme temperatures (increasing/extreme heat)	-The risks of preterm birth (PTB) increased with exposures to heat events in gestational week 1–4, 21–32, and the four weeks before delivery.<br>- The threshold was determined as the daily maximum temperature at 90th percentile of distribution or 30ºC lasting for at least one day, lower than the current definition of heatwave recommended by the China Meteorological Administration.<br>-Approximately 15% or 17% of the number of total PTB cases could be avoided if women avoided the heat exposures after activated early warning systems triggered by these thresholds.
Wang et al.	Temperature variability and birthweight: Epidemiological evidence from Africa	2023	African continent [n=37] (See article for more details)	Mixed	Quantitative	Newborns to mothers aged 15-49 recorded in the DHS across study countries from 1990-2020 [n=333618]	ECMWF	Extreme temperatures (increasing/extreme heat)	-Compared to the reference temperature variability (TV) where the lowest risk was observed, extremely high (97.5th percentile) overall, intraday, and interday TV during the entire pregnancy increased the odds of low birth weight (LBW) birth by 37.3%, 24.1%, and 15.1% respectively.<br>-In total, 7.3% of all LBW births in Africa were attributable to elevated overall TV.<br>-These associations were observed in dry climate zones, but not in tropical or temperate zones.
Wu et al.	Effects of ambient temperature and relative humidity on preterm birth during early pregnancy and before parturition in China from 2010 to 2018: A population-based large-	2023	China (upper middle)	Urban	Quantitative	Women of childbearing age (18-49 yrs) who responded to the National Free Preconception Health Examination Project from 2010 to 2018 [n=205771]	China National Meteorological Information Center	Extreme temperatures (increasing/extreme heat)	- High temperatures and extremely high temperatures were both risk factors for preterm labour during all four exposure windows.<br>-Extremely low temperatures and low temperatures at 1 and 4 weeks of pregnancy were both risk factors for preterm birth (PTB).<br>-Extremely low temperatures and low temperatures at 1 and 4 weeks before delivery were protective factors for PTB.

	sample cohort study								-Low humidity was a protective factor for PTB.<br>-High humidity was a risk factor for PTB.
Yu et al.	Associations between ambient heat exposure early in pregnancy and risk of congenital heart defects: A large population-based study	2021	China (upper middle)	Mixed	Quantitative	Newborns born between 2015- 2019 in Guangdong to mothers aged 16-50 reported in the Provincial Women and Children Health Information System [n=1918105]	China Meteorological Data Sharing system	Extreme temperatures (increasing/extreme heat)	-Extreme heat events between 2- and 8-weeks post conception were associated with increased prevalence of overall congenital heart disease (CHD) in the offspring.<br>-The risks of CHD were higher among those who were exposed to more extreme heat or in a higher frequency, duration, or cumulative days of the extreme heat events.
Andalón et al.	Weather shocks and health at birth in Colombia	2014	Colombia (upper middle)	Rural	Quantitative	Newborns from rural and semi-rural areas registered in the Colombian national registry from 1999-2008 [n=1500000]	Climate Research Unit of the University of East Anglia	Extreme temperatures (increasing/extreme heat & decreasing temperature/ cold spells)	-Exposure to moderate low-temperature shocks (cold waves) during the first and second trimesters of pregnancy was associated with lower length at birth, irrespective of mother's age or education level.<br>-Exposure to moderate heat waves (high- temperature shocks) during the third trimester of pregnancy increased the risk of having below average birthweight.<br>-Significant negative effects of high-temperature shocks on normal Apgar scores
Chen et al.	Effect on the health of newborns caused by extreme temperature in Guangzhou	2022	China (upper middle)	Urban	Quantitative	Neonates from a large hospital in Guangzhou between 2017-2019 [n=64270]	China Meteorological Administration	Extreme temperatures (increasing/extreme heat & decreasing temperature/ cold spells)	-The Apgar score of the newborns decreased by 0.008 (0.029%) when the duration of extreme temperature was extended by one day.
Chen et al.	The effects of prenatal exposure to temperature extremes on birth outcomes: The case of China	2020	China (upper middle)	Rural	Quantitative	Newborns, live singleton births collected from China's National Disease Surveillance Points system from 1991-2000 in 31 rural Chinese provinces from 1991-2000 [n=637033]	China National Meteorological Data Service Center	Extreme temperatures (increasing/extreme heat & decreasing temperature/ cold spells)	-Spending an additional day in the gestation period with a temperature above 28 °C, relative to a day in the 0–4 °C range, leads to a reduction in birth weight by 0.050 percent (1.66 grams).<br>-Exposure to an additional hot day above 28 °C during gestation increased the risk of low birthweight by 0.035

									percentage points.<br>-No significantly detrimental effect on birth weight for survived newborns who are exposed to extremely cold days in utero.
Li et al.	The relationship between extreme ambient temperature and small for gestational age: A cohort study of 1,436,480 singleton term births in China	2023	China (upper middle)	Mixed	Quantitative	Singleton newborns to mothers living in the Hubei Province that weighed between 1000 and 5500g recorded by the Health Commission of Hubei Province from 2014-2016 [n=1436480]	National Meteorological Information Center of China	Extreme temperatures (increasing/extreme heat & decreasing temperature/ cold spells)	-Small for gestational age is significantly associated with both cold and heat exposure in the eastern region, primarily with heat exposure in the middle region, and with neither cold nor heat exposure in the western region.<br>-Significant association mostly occurred in the third trimester.
Molina et al.	The perils of climate change: In utero exposure to temperature variability and birth outcomes in the Andean region	2016	Bolivia (lower middle), Colombia (upper middle) and Peru (upper middle)	Mixed	Quantitative	Newborns, single birth children of weight 500-600g whose mothers (aged 15-45) lived in the municipality for at least two years before the child's birth who responded to the Bolivia, Colombia, and Peru DHS rom 1990-2013 [n=86021]	Terrestrial Air Temperature: 1900-2019 Gridded Monthly Time Series Version 3.01 (University of Delaware's Center for Climatic Research: 0.5 X 0.5° Matsurra & Willmott series)	Extreme temperatures (increasing/extreme heat & decreasing temperature/ cold spells)	-Exposure in utero to higher long-term local temperature reduces birth weight and increases the risk of being born with low weight.
Sun et al.	Potential impact of ambient temperature on maternal blood pressure and hyptertensive disorders of pregnancy: A nationwide multicenter study based on the China birth cohort	2023	China (upper middle)	Mixed	Quantitative	People with normal blood pressure before pregnancy enrolled in the China Birth Cohort Study from 2017-2021 [n=105063]	ECMWF	Extreme temperatures (increasing/extreme heat & decreasing temperature/ cold spells)	-There are positive associations of cold exposure in the second and third trimesters with maternal blood pressure as well as odds of hypertensive disorders prevalence.<br>- Pregnant women aged ≥35 years and those coming from North China may be more vulnerable to the hypertensive effects of cold exposure.
Wang et al.	Effects of gestational	2023	China (upper middle)	Urban	Quantitative	Sibling pairs born as singletons with a	China Meteorological	Extreme temperatures	-Maternal heat and cold exposure were associated with

	ambient extreme temperature exposures on the risk of preterm birth in China: A sibling-matched study based on a multi-center prospective cohort					gestation age of > 28 weeks and <44 weeks to mothers > 18 yrs and <50 yrs recorded in the National Maternal and Newborn Health Monitoring Project from 2013-2018 [n=10826]	Data Service Center	(increasing/extreme heat & decreasing temperature/ cold spells)	an increased risk of preterm birth (PTB).<br>-Effect of heat exposure on PTB was stronger for the firstborns.<br>-Effect of cold exposure was stronger for the second-borns.<br>-Second-borns with short inter-pregnancy intervals may be more susceptible to heat exposure.
Wulayin et al.	The mediation of the placenta on the association between maternal ambient temperature exposure and birth weight	2023	China (upper middle)	Urban	Quantitative	Pregnant women aged 18-50 recruited at a hospital in Guangzhou from 2017-2020 [n=3349]	China Meteorological Administration Land Data Assimilation System	Extreme temperatures (increasing/extreme heat & decreasing temperature/ cold spells)	-High and low temperatures during the pregnancy were associated with lower birth weight, placental weight, placental volume, and a higher placental-to-birth weight ratio.<br>-The placental weight partially mediated the association between suboptimal temperature and birth weight.
Xiao et al.	Evidence of interactive effects of late-pregnancy exposure to air pollution and extreme temperature on preterm birth in China: A nationwide study	2023	China (upper middle)	Mixed	Quantitative	Newborns recorded in the China Birth Cohort Study from 2017- 2020 [n=103040]	ECMWF, ground observations	Extreme temperatures (increasing/extreme heat & decreasing temperature/ cold spells)	-Higher temperature levels were observed among preterm birth (PTB) cases.<br>-Cold temperature extremes seemed to pose higher adverse effects on PTB in south than north China.
Xu et al.	The prenatal weekly temperature exposure and neonatal congenital heart disease: A large population-based observational study in China	2023	China (upper middle)	Urban	Quantitative	Newborns with congenital heart disease (CHD) reported in the Network Platform for Congenital Heart Diseases across 11 cities in eastern China from 2019-2020 [n=5904]	Shanghai Qingyue	Extreme temperatures (increasing/extreme heat & decreasing temperature/ cold spells)	- The temperature-CHD combination performed positive significance in two exposure windows, gestational weeks 10–16 and 26–31, and reached the maximum effect in the 28th week.<br>-Compared with extreme cold, these effects were higher in extreme heat.<br>- A degree centigrade increase in temperature exposure was associated with the increment of CHD risk in the first and second

									trimesters, especially in extreme heat.<br>-Neonates born in lower education regions were more vulnerable to temperature-related CHDs.
Yu et al.	Extreme temperature exposure and risks of preterm birth subtypes based on a nationwide survey in China	2023	China (upper middle)	Mixed	Quantitative	Singleton newborns with gestational age of ≥24 weeks or ≥500 grams whose mother was aged 15-49 years recorded in the China Labor and Delivery Survey from 2015-2017 [n=70818]	National Meteorological Data Center	Extreme temperatures (increasing/extreme heat & decreasing temperature/ cold spells)	-Exposure to extreme cold temperatures during pregnancy increase the preterm birth (PTB) risk, particularly for late PTB, spontaneous PTB, and PPROM and are more pronounced in the western and northern regions and for women with female fetuses.<br>- The association of heat waves with PTB was unstable.
Liang et al.	Effect of the 2008 cold spell on preterm births in two subtropical cities of Guangdong Province, Southern China	2018	China (upper middle)	Urban	Quantitative	Newborns reported in the birth registry database from health facilities in Dongguan and Shenzhen from 2006-2010 [n=904795]	Local meteorological bureaus	Extreme temperatures (decreasing temperature/ cold spells)	-Cold spell could increase the risk of preterm birth in Dongguan and Shenzhen.<br>-The effect of the cold spell on preterm births lasts for more than one week.<br>-Pregnant women of 34–36 gestational weeks and maternal age <35 years were more susceptible to the cold spell on the risk of preterm birth.
Bakhtsiyarava et al.	Climate, birth weight, and agricultural livelihoods in Kenya and Mali	2018	Mali (low) and Kenya (lower middle)	Mixed	Quantitative	Newborns recorded in Kenya DHS in 2008 and 2014 [n=9584] and Mali DHS in 2006 and 2012 [n=3416]	Integrated Public Use Microdata Series -Terra, Normalized Difference Vegetation Index from Moderate Resolution Imaging Spectroradiometer	1. Extreme temperature (increasing/extreme heat)<br>2. Rainfall shocks (negative)	-An additional 100 mm of rainfall during the 12-month before birth was associated with increase in birth weight among farmers.<br>-Every additional hot month in food-cropping communities in Kenya was associated with a significant decrease in birth weight.<br>-Average temperatures above 35 °C in a year preceding birth was associated with reduced birth weight among farmers in Kenya.<br>-Observed a positive significant association between birth weight and precipitation for food croppers in both Kenya and Mali.

Grace et al.	Linking climate change and health outcomes: Examining the relationship between temperature, precipitation and birth weight	2015	African continent [n=19] (See article for details)	Mixed	Quantitative	Newborns reported in the DHS across 19 African countries [n≈70000] and their mothers who are siblings, had at least two birth in the five years prior to the survey, and have resided in the same community for the past five years [sample size not found]	CHIRPS, The National Center for Environmental Prediction's reanalysis	1. Extreme temperature (increasing/extreme heat) 2. Rainfall shocks (negative & positive)	-Higher amounts of precipitation indicate larger birth weights in all trimesters. -An increase in the number of days above 100 F during any trimester corresponds to a decrease in birth weight. There is a larger effect when the temperature threshold is increased for the count of hot days to 105 F. -More days above 100 F during the first through third trimesters are correlated to lower birth weights. This has a larger effect for days above 105 F.
Abdullah et al.	Effects of climate change and maternal morality: Perspective from case studies in the rural area of Bangladesh	2019	Bangladesh (lower middle)	Rural	Qualitative	IDIs with guardians of pregnant or recently delivered mothers, village doctors, and birth attendants [n=8] FGDs with neighbours of the deceased mother's family, male and female guardians of pregnant and recently delivered mothers, pregnant women, community group members, school teachers, religious leaders, Union Parisad members, and elite people of the society who have an idea on the incidence of maternal death in 2015 [n= between 27 and 33]	United Nations, Bangladesh Water Development Board, Ministry of Disaster Management and Relief	Floods	-Major causes of maternal death during floods: negligence of maternal healthcare, unavailability of facilities and proper care services, dependency on unqualified doctors, communication and transportation problems, and barriers to referral of the pregnant women experiencing complications during floods
Barman et al.	Association between natural hazards and postnatal care among the neonates in India: A step towards full coverage using	2023	India (lower middle)	Mixed	Quantitative	Children born five years preceding the survey and who are the youngest child whose mothers responded to the National Family Health Survey from 2019-2021 [n=176843]	The Vulnerability Atlas of India	Floods	-Children in flood effected areas were 0.15 times less likely to get postnatal care (PNC) coverage than non-flood areas compared to the reference category. -High floods and high PNC access can be observed in Punjab, and low PNC and high flood clusters are concentrated in easter Uttar Pradesh, Bihar and West Bengal.

	geospatial approach								-PNC services in hazard-prone areas are much lower than the national average.
Maheen et al.	Rural women's experience of living and giving birth in relief camps in Pakistan	2017	Pakistan (lower middle)	Rural	Qualitative	IDIs with women who gave birth during the floods in Sindh Province in 2011 [n = 15]	Context-specific literature	Floods	-Women reported lack of support, of autonomy to make decisions, and of SRHR services, making them give birth in inappropriate conditions.<br>-An increase in stillbirth was observed for pregnant women who experienced a day within the week prior to giving birth with smaller diurnal temperature ranges though socioeconomic status also influences these risks.
Saulnier et al.	Staying afloat: Community perspectives on health system resilience in the management of pregnancy and childbirth care during floods in Cambodia	2020	Cambodia (lower middle)	Rural	Qualitative	IDIs with village chiefs, village health support groups, traditional birth attendants, paternal or maternal grandmothers and commune administrators who were expected to have experience with maternal health needs or contact with pregnant women during floods [n=17]<br>FGDs with women aged 18+ who were pregnant or delivered during the most recent flood or men whose partner met the same criteria [n=41] in 2018	Context-specific literature	Floods	-Main themes to handle difficulty during flood were hoping for support from others but not relying on it and navigating mistrust in the system.<br>-Information sharing appeared as a mostly one-way, top-down process from the health system to community members, and community members lacked the power to advocate for their preferred care.<br>-Although the community was able to manage their health needs during floods, the ostensible lack of involvement in decision-making may be putting their capability at risk.
Abiona et al.	The impact of timing of in utero drought shocks on birth outcomes in rural households: Evidence from Sierra Leone	2022	Sierra Leone (low)	Rural	Quantitative	Newborns born to women aged 15-49 who responded to the Sierra Leone DHS in 2008 and 2013 [n=4357]	University of Delaware's Center for Climatic Research: 0.5 X 0.5° Matsurra & Willmott series	Rainfall shocks (negative & positive)	-Harvest drought shock leads to an overall decrease of around 4% in birthweight (corresponding to an approximately 59% increase in low birthweight incidence) compared to the baseline mean.<br>-Contemporaneous gestation drought shock is correlated with an increase in low birthweight incidence of approximately 54% when compared to the mean.
Le et al.	The impacts of rainfall shocks	2022	Vietnam (lower middle)	Mixed	Quantitative	Newborns born between 2004-2011	Meteorological and Hydrological	Rainfall shocks	-Excessive and deficient rainfall shocks in the second trimester

	on birthweight in Vietnam					reported in the Vietnam Multiple Indicator Cluster Survey (MICS3 and MICS4) [n=1961]	Administration of Vietnam	(negative & positive)	reduces child's weight at birth by 3.5% (97 grams) and 3.1% (87 grams).<br>-Infants born to poor, rural, and low-educated mothers are especially vulnerable.
Nguyen et al.	Rainfall and birth outcome: Evidence from Kyrgyzstan	2022	Kyrgyz Rep. (lower middle)	Mixed	Quantitative	Newborns with mothers aged 15–49 who responded to the Kyrgyzstan DHS in 2012 (wave 6) [n=3962]	Global Historical Climatology Network-monthly Climatic Research Unit Time Series (provided by the United Kingdom's National Center for Atmospheric Science)	Rainfall shocks (positive)	-Prenatal exposure to rainfall shocks was negatively associated with birth weight.<br>-Children whose mothers are poor and/or live in rural areas were more affected.
Rocha et al.	Water scarcity and birth outcomes in the Brazilian semiarid	2014	Brazil (upper middle)	Mixed	Quantitative	Newborns in the Brazilian semiarid region born from 1996 to 2010 and were registered in the Brazilian National System of Information on Birth Records or in the Brazilian National System of Mortality Records [n=~5400000]	Terrestrial Air Temperature and Terrestrial Precipitation: 1990-2010 Gridded Monthly Time Series 3.01 and 3.02 (University of Delaware's Center for Climatic Research: 0.5 X 0.5° Matsurra & Willmott series), Brazilian Censuses	Rainfall shocks (negative)	-Negative rainfall shocks are associated with higher infant mortality, lower birth weight, and shorter gestation periods.<br>-The rainfall shocks' effect are higher when it occurs during the 2nd trimester of gestation and when the birth occurs during dry season.
Sato et al.	Immediate needs and concerns among pregnant women during and after Typhoon Haiyan (Yolanda)	2016	Philippines (lower middle)	Rural	Qualitative	FGDs with women who were pregnant at the time of the typhoon Haiayan in 2014 [n = 53]	Context-specific literature	Cyclones/ typhoons	-Pregnant women reported experiencing abnormal pregnancy symptoms during evacuation, lack of medical care and uncertainty regarding pregnancy status.
Bryson et al.	Seasonality, climate change, and food security	2021	Uganda (low)	Rural	Qualitative	FGDs with women aged 18+ from the indigenous Batwa community [n=24] or	Context-specific literature	Multiple	-The interaction between physical health and food security was cyclic; illness during pregnancy decreased food

	during pregnancy among indigenous and non-indigenous women in rural Uganda: Implications for maternal-infant health					the non-indigenous Bakiga community [n=22] who have had one pregnancy or are currently pregnant during 2017			procurement, and consequent undernutrition intensified illness, making it even harder for women to access food<br>-Indigenous Batwa women described a heightened sensitivity to climate-related decreases in food security compared to neighbouring Bakiga women.
**GENDER-BASED VIOLENCE (n= 9)**									
Becerra-Valbuena et al.	Gendered migration responses to drought in Malawi	2021	Malawi (low)	Mixed	Quantitative	Men and women migrants between districts [n=127000] and within districts [n=182000] who responded to the Living Standards Measurement Study-Integrated Surveys on Agriculture (LSMS-ISA) in in 2013	Standardized indices calculated from gridded satellite data including the standardized precipitation index (SPI) and the standardized precipitation-evapotranspiration index (SPEI)	Drought	-Evidence of increases in children's migration for work following drought, mainly for boys, and to a lesser extent for girls.<br>-Only when combining the work and marriage-related motives that we find a sizeable increase in the probability of migration of girls aged 10–17 following drought.
Chigusiwa et al.	Drought and social conflict in rural Zimbabwe: Does the burden fall on women and girls?	2022	Zimbabwe (lower middle)	Rural	Quantitative	Men and women from rural households who responded to the Zimbabwe Vulnerability Assessment Committee survey in 2020 [n=11971]	Zimbabwe Vulnerability Assessment Committee survey	Drought	-Drought shock associated with increased household propensity to experience water point violence.<br>-Severity of the drought shock increased the risk of experiencing water point violence.<br>-Drought shock–induced water point violence is only statistically valid for households where the water-fetchers are woman or girl.
Cooper et al.	Re-examining the effects of drought on intimate-partner violence	2021	Sub-saharan Africa, Latin America and Caribbean [n=40] (See article for details)	Mixed	Quantitative	Women between the ages of 15-49 who responded to the DHS across 40 countries in SSA, LAC, and Asia from 2000-2018 [n=363428]	CHIRPS dataset	Drought	-No significant association between any level of drought and emotional or physical violence on any continent.<br>-Drought had a strong and significant association with controlling behaviours on all three continents.
Epstein et al.	Drought and intimate partner	2020	Sub-Saharan Africa [n=19]	Rural	Quantitative	Women aged 15-49 who are married or partnered and	CHIRPS dataset	Drought	-Women living in severe drought had higher risk of reporting a controlling partner,

	violence towards women in 19 countries in sub-Saharan Africa during 2011-2018: A population-based study		(See article for details)			responded to the DHS from 2011-2018 across 19 sub-Saharan African countries [n=83990]			and physical and sexual violence compared with women not experiencing drought.<br>-Women living in mild/moderate drought had higher risk of reporting physical and sexual violence compared with those not living in drought.<br>-3 settings where drought was protective for at least 1 measure of intimate partner violence: Namibia, Tanzania, and Uganda.<br>-Stronger associations between drought and intimate partner violence among adolescent girls and unemployed women.
Hossen et al.	Gendered perspectives on climate change adaptation: A quest for social sustainability in Badlagaree Village, Bangladesh	2021	Bangladesh (lower middle)	Rural	Qualitative	FGDs with women aged 40+ living in Badlargaree village (Gaibandha district) in 2018 [n=47]	Context-specific literature	Drought	-Women in Bangladesh are subject to multiple vulnerabilities related to drought. They include unequal wages and access to employment, unsafe employment, domestic violence, food insecurity, inequitable access to education, discrimination via the dowry system and gender-based violence.<br>-Many of these vulnerabilities are pre-existing and while not specifically caused by drought are nonetheless being magnified by its environmental impacts due to embedded social and cultural practices.
Rezwana et al.	Gender-based violence before, during, and after cyclones: Slow violence and layered disasters	2020	Bangladesh (lower middle)	Mixed	Qualitative	KIIs with local residents, municipal and local governments, and NGOs IDIs with female survivors of GBV aged 17-50 and males including GBV	Comprehensive Disaster Management Programme, Network for Information, Response and Preparedness	Cyclones/Typhoons	-GBV (early marriage, sexual abuse and rape, physical and psychological violence, etc.) was already present before the cyclone hit the Barguna district but was aggravated by the cyclone.<br>-GBV made women more

						perpetrators and survivors [n=45]<br>FGDs [Sample size not found]<br>Periods of observation in 2016	Activities on Disaster		vulnerable to negative impacts of future natural disasters.
Allen et al.	Kenyan women bearing the cost of climate change	2021	Kenya (lower middle)	Mixed	Quantitative	One woman from each household of childbearing age (15-49 yrs) and had ever been married or lived with a man who responded to the Kenya DHS in 2008 [n=4903] and 2014 [n=4512]	EM-DAT	Floods	-There are greater odds of reporting intimate partner violence (IPV) in counties that experienced a severe flood when compared to counties that did not experience a severe flood, specifically physical and sexual violence.
Alam et al.	Women in natural disasters: A case study from southern coastal region of Bangladesh	2014	Bangladesh (lower middle)	Rural	Quantitative, qualitative	Quantitative: vulnerable women who responded to a household questionnaire in 2013 [n=105],<br><br>Qualitative:<br>In 2013, KIIs with Union Parishad (UP) Chairman, male and female UP members, UP secretary, schoolteacher, and social workers/volunteers [n=10]<br>FGDs with community people from different groups, occupations, knowledgeable persons and local NGOs' officials [4 FGDs]<br>Public consultation with community people from different groups, occupations, incomes and knowledgeable persons [series]<br>Case study with vulnerable women [n=6]	World Bank, Government of Bangladesh	Multiple	-Disasters impact pregnant women, adolescent, and elderly women (e.g., poor transport system and deliver without care).<br>-High risk for sexual harassment for women due to isolation/separated from family members, not under observation, ask for support or become male-dependent, and are in close physical proximity to males.<br>- Male members are not interested to take women to cyclone shelters due to an insecure and non-private environment.<br>- Lack of privacy at the shelter including no separate toilet and washroom facilities, insufficient medicine supply, lack of fresh water supply, harassment, and lack of consideration for women's special needs.
Diaz et al.	A drop of love? Rainfall shocks and	2023	Peru (upper middle)	Rural	Quantitative	Female household heads of reproductive age (15-49 yrs) that are	University of Delaware's Terrestrial	Rainfall shocks	-The prevalence of recent physical intimate partner violence increases by 65 percent

	spousal abuse: Evidence from rural Peru					married/cohabitating with their partners who have lived in the municipality for at least one year and responded to the Peru DHS from 2005-2014 [n=15110]	Precipitation: Gridded Monthly Time Series C. 5.01	(negative & positive)	after exposure to a dry, but not a wet, shock during the last cropping season.<br>-There is a 75 percent increase in the probability that women experience sequelae from physical abuse in the form of bruises and lesions on their bodies.<br>-Economic insecurity, poverty-related stress, emotional well-being, and women's empowerment are central to explaining this relationship.
**HIV AND OTHER STIS (n=9)**									
Austin et al.	Drying climates and gendered suffering: Links between drought, food insecurity, and women's HIV in less-developed countries	2021	All countries that occupy the lower 3 quartiles of GDP per capita in 2010 across regions [n=91] (See article for details)	Mixed	Quantitative	Females aged 15+ living with HIV across 91 lower-income countries as captured by 2019 World Bank data [sample size not found]	EM-DAT	Drought	-Suffering from drought puts women at disproportionate risk of acquiring HIV in less-developed countries through increased food insecurity.<br>-Food insecurity reduces women's socio-health status, which in turn, is associated with lower contraceptive use.
Epstein et al.	Drought, HIV testing, and HIV transmission risk behaviors: A population-based study in 10 high HIV prevalence countries in SSA	2022	Sub-Saharan Africa [n=10] (See article for details)	Mixed	Quantitative	Women and men aged 15-49 who responded to the DHS between 2011-2016 across 10 countries with high HIV prevalence in Sub-Saharan Africa [n=206205]	CHIRPS dataset	Drought	-Drought was associated with lower HIV testing over the past 12 months and higher probability of condomless sex at their last sexual encounter.<br>-The negative association between drought and HIV testing in the past 12 months was strongest among men, adolescents, and people living in urban areas.
Iwuji et al.	The impact of drought on HIV care in rural South Africa: An interrupted time series analysis	2023	South Africa (upper middle)	Rural	Quantitative	Individuals aged 15-59 who were registered for HIV care and started ART in one of the 17 public ART clinics in the Hlabisa sub-districts between 2010-2018 and clinic visits through 2019 [n=40714]	Climate Research Unit Version 4.06 dataset	Drought	-There was a marked decrease in ART adherence as measured by MPR during the drought years with no full recovery of adherence during the wet years.<br>-The decrease in adherence differed by sex and age with adherence being worse in women than men and in those aged between 15 and 24.

									-While adherence in women returned to the level in men in Jan 2017 to Dec 2018, this recovery in adherence did not occur in younger individuals.<br>-There was a marked drop in retention in care at the start of the drought years followed by some recovery and then, a dip again towards the end of the observation period.<br>-The decrease in retention in care was more marked for women than men and in younger individuals than older individuals.
Kuulei Berndt et al.	Drought and disproportionate disease: An investigation of gendered vulnerabilities to HIV/AIDS in less-developed nations	2020	Countries falling within lower three quartiles of World Bank income classification across regions [n=69] (See article for details)	Mixed	Quantitative	Women and men aged 15-49 living with HIV across 65 lower-income countries as captured by 2018 World Bank data [sample size not found]	EM-DAT	Drought	-Evidence of a robust, consistent positively statistically significant relationship between droughts and women's proportion of the population living with HIV in less-developed countries, net of other factors.<br>-There is an inconsistent and relatively modest impact on HIV prevalence.
Low et al.	Association between severe drought and HIV prevention and care behaviors in Lesotho: A population-based survey 2016–2017	2019	Lesotho (lower middle)	Mixed	Quantitative	Female and male adults aged 15–59 who responded to the Lesotho Population-Based HIV Impact Assessment in 2016-2017 [n=12887]	CHIRPS dataset	Drought	-Drought in Lesotho was associated with higher HIV prevalence in girls 15–19 years old in rural areas and with lower educational attainment and riskier sexual behaviour in rural females 15–24 years old.
Treibich et al.	From a drought to HIV: An analysis of the effect of droughts on transactional sex and STIs in Malawi	2022	Malawi (low)	Mixed	Quantitative	Unmarried women aged 15–24 and men aged 15-54 who responded to the Malawi DHS in 2015-216 [n=17088]	Global Precipitation Climate Centre monthly precipitation dataset	Drought	-Economic shocks increase risky sexual behaviours among women, especially among those working in agriculture.<br>-Among them, the likelihood of engaging in transactional sex is doubled during drought time and the HIV prevalence is increased by 15%.<br>-Droughts also increased the prevalence of HIV among

									women who do not work in agriculture.
Githinji et al.	Compound vulnerabilities: The intersection of climate variability and HIV/AIDS in northwestern Tanzania	2014	Tanzania (lower middle)	Rural	Qualitative	IDIs with heads of households in Nsisha including those who were married, single, and widowed men and women 2005-2006 [n=311]	Context-specific literature	1) Drought 2) Rainfall shocks (positive)	-Climate change exacerbates deepening poverty, food and nutrition insecurity and poor health which compounds vulnerability to HIV/AIDS (ex. single mothers forced to sell sex).
Nagata et al.	Analysis of heavy rainfall in Sub-Saharan Africa and HIV transmission risk, HIV prevalence, and sexually transmitted infections, 2005-2017	2022	Sub-Saharan Africa [n=21] (See article for details)	Mixed	Quantitative	All women aged 15-49 and men aged 15 to 59 years who responded to the DHS across 21 countries in sub-Saharan Africa in 2005-2017 [n =288333]	Standardized Precipitation Index accessed from the Columbia Climate School International Research Institute Climate Data Library	Rainfall shocks (positive)	-Exposure to rainfall was positively associated with higher odds of HIV, other STIs and number of sexual partners.
Anthonj et al.	The impact of flooding on people living with HIV: A case study from the Ohangwena Region Namibia	2015	Namibia (upper middle)	Rural	Qualitative	IDIs with women living with HIV [n=7]<br>FGDs with civil society organization and public sector institution representatives [n=16]<br>KIIs with disaster risk management officer, representative of Emergency Management Unit, primary healthcare supervisor, NGO chairperson, representative of the Red Cross [n=5]<br>National Feedback Meeting [sample size not found]	Context-specific literature	Floods	-Conditions that normally threaten people living with HIV are intensified by flood-related breakdown of infrastructure, insecurity, malnutrition, diseases.<br>-Increased risk both of infection and disease due to the inaccessibility of health services and antiretroviral treatment.
**FERTILITY CARE (n=7)**									
Thiede et al.	Climate anomalies and birth rates in	2022	Sub-Saharan Africa [n=23] (See article for details)	Mixed	Quantitative	Newborns reported in the DHS across 23 countries in sub-Saharan Africa from	CHIRPS dataset, Terrestrial Hydrology Research Group	1) Extreme temperatures (increasing/extreme heat	-Women who experienced above-average temperatures or below-average precipitation during a given 12-month period

	sub-Saharan Africa					1982-2017 [sample size was 4,961,381 person-years of observations]	at Princeton University, Climate Research Unit's Time Series, National Centers for Environmental Prediction-National Center for Atmospheric Research Reanalysis	& decreasing temperature/ cold spells) 2) Rainfall shocks (positive & negative)	are significantly less likely to experience a live birth in the subsequent year than comparable women exposed to the average conditions for their locality.<br>-Spells of below-average temperatures and above- average precipitation were associated with statistically significant increases in birth rates during the following year.
Chen et al.	The link between climate change, food security and fertility: The case of Bangladesh	2021	Bangladesh (lower middle)	Mixed	Quantitative	Newborns in Bangladesh from 1966-2015 as reported by the United Nations Population Division [sample size not found]	Bangladesh Meteorological Department	1) Extreme temperature (increasing/extreme heat) 2) Rainfall shocks (positive)	-The total effect of maximum temperature on total fertility rate (TFR) is significantly negative, mainly because of the negative direct effect. It means that as maximum temperature increases, TFR decreases.<br>-Fertility is found to respond negatively to maximum temperature in the short run but positively in the long run.<br>-Fertility responds positively to the mean rainfall in the short term, it is insensitive to the long term.
Eissler et al.	Climatic variability and changing reproductive goals in Sub-saharan Africa	2019	Sub-Saharan Africa (low- and lower-middle) [n=18] (See article for details)	Mixed	Quantitative	Women aged 15-49 who married or began to cohabitate with a partner for the first within 10 years prior to the survey and responded to the DHS across 18 countries in sub-Saharan Africa between 1990-2015 [n=70879]	University of East Anglia Climate Research Unit's Time Series (version 3.24)	1) Extreme temperatures (increasing/extreme heat) 2) Rainfall shocks (positive & negative)	-Exposure to higher temperatures during the 12- and 60-month periods prior to the DHS interview is associated with lower ideal family size (IFS)<br>-Overall exposure to higher temperatures reduced women's desire to want a first or additional child.<br>-Exposure to precipitation anomalies during the 12 months prior to each survey is associated with a significant reduction in IFS, but positive effects of precipitation anomalies on IFS were found.<br>-Unusually high temperatures are associated with declines in IFS and preferences only in rural areas, where unfavourably warm conditions can hinder agricultural production.

									-The effects of short-term temperature shocks were consistent across parities, highlighting differences between short- and long-term ideational responses among high-parity women.<br>-In West Africa, women's fertility goals vary (positively) with precipitation patterns, while in East and Southern Africa, fertility goals are primarily driven by changing temperatures.
Deng et al.	Heat wave exposure and semen quality in sperm donation volunteers: A retrospective longitudinal study in south China	2023	China (upper middle)	Urban	Quantitative	Sperm donation volunteers who lived in Guangdong and intended to donate sperm at the Guangdong provincial human sperm bank between June 22, 2018 and December 31, 2019 [n=2183]	China Meteorological Administration Land Data Assimilation System	Extreme temperatures (Increasing/extreme heat)	-Exposure to heat waves during 0-90 days before ejaculation was significantly associated with a reduction in semen quality (windows of susceptibility varied for sperm count, motility, and morphology).
Haq et al.	Underlying causes and the impacts of disaster events (floods) on fertility decisions in rural Bangladesh	2018	Bangladesh (lower middle)	Rural	Quantitative, qualitative	Quantitative: married and unmarried men and women in Sharat Pur aged 16-65+ who responded to a questionnaire [n=158]<br>Qualitative:<br>IDIs with female and male Sharat Pur residents [n=15]	Context-specific literature	Floods	-People who are more concerned about the impacts of extreme weather events (floods) are more likely to reduce their preference for having many children due to perceived difficulties during flood events such as managing food and moving children/belongings.<br>-Respondents expressed their desire to have more sons or at least as many children as they have as they are only concerned about extreme weather events<br>-Sharat Pur is highly vulnerable and at risk of extreme floods, and people living in the village do not perceive the risk of their children dying to influence them having more children.
Haq et al.	Is fertility preference related to perception of the risk of child	2019	Bangladesh (lower middle)	Rural	Quantitative	Quantitative: ever-married women of reproductive age (15–49 years) who had given birth at least once and were currently	Context-specific literature	1) Floods<br>2) Cyclones	-An experience of child death, a perception of the risk of child death, and fertility preferences are higher in areas vulnerable to extreme weather events.

	mortality, changes in landholding, and type of family? A comparative study on populations vulnerable and not vulnerable to extreme weather events in Bangladesh					living together with their husbands who responded to a questionnaire [n=759]			-There is an association of perception of risk of child death and fertility preference which is only significant in areas that are vulnerable to extreme weather events.<br>- In areas vulnerable to extreme weather events, fertility preferences (the preference to have more children) is nearly six times higher for women with a perceived risk of child death.
Haq	The impact of extreme weather events on fertility preference and gender preference in Bangladesh	2023	Bangladesh (lower middle)	Mixed	Quantitative	Women aged 20-24 and men aged 30-34 with at least one child who live in flood-prone, drought-prone and cyclone-prone places [n=177]	Context-specific literature	Multiple	-The gender of the first child, the perceived risk of infant death due to extreme weather event (EWE), the opinion on having more children to recover from the damage and losses caused by EWE, government and non-governmental organization (NGO) support during EWE, and the intended timing of childbearing (after or before EWE) are all significant factors influencing fertility preferences and gender preferences.<br>-There were larger differences between flood-prone areas and drought- and cyclone-prone areas in regard to preferences for fertility and gender.
**HARMFUL PRACTICES (n=7)**									
Corno et al.	Age of marriage, weather shocks, and the direction of marriage payments	2020	Sub-Saharan Africa and India [n=32] (See article for details)	Mixed	Quantitative	All women in the household aged 15-49 who were born between 1950 and 1989 and were married or unmarried who responded to the Sub-Saharan Africa DHS from 1994-2013 [n=326645] and ever-married women aged 15-49 who responded to the India DHS in 1998 and India Development Survey in 2004-2005 [n=66466]	University of Delaware Air Temperature and Precipitation project, Food and Agricultural Organization, World Bank	Drought	-Drought raised the annual hazard of marriage between ages 12 and 17 by 3% in sub-Saharan Africa (SSA), and it decreased the hazard by 4% in India.<br>-In SSA, a drought was associated with a 4% increase in the annual probability of childbearing before turning 18.<br>- Experience of drought during the teenage years increased the total number of children a woman reports by 0.06 or 1% in SSA.<br>-Droughts significantly associated with child marriage in

									countries and ethnic groups that traditionally pay bride price.<br>-When droughts have a greater impact on a household consumption, they also have a stronger effect on child marriage.
Esho et al.	Intersections between climate change and female genital mutilation among the Maasai of Kajiado County, Kenya	2021	Kenya (lower middle)	Rural	Qualitative	KIIs with county executives and officers representing ministries of Kajiado County, community elders and parents [n=12]<br>FGDs with young women beneficiaries of a TVET program implemented by the country government aged 18-25 in 2020 [n=8]	Global Climate Modelling data	Drought	-High prevalence of female genital mutilation and child marriage among the Maasai of Kajiado county may be largely perpetuated by the poorly understood intersections between climate change and the widening gender inequalities, which render girls and women more vulnerable to harmful practices and socio-economic disempowerment due to a lack of education.
Carrico et al.	Extreme weather and marriage among girls and women in Bangladesh	2020	Bangladesh (lower middle)	Rural	Quantitative	Female household heads who entered into a first marriage between 1989 and 2013 and were either born in the study area or migrated into the area at least one year prior to the first marriage who responded to the Bangladesh Environment Survey in 2014 [n=505]	Bangladesh Meteorology Department	1) Extreme temperatures (increasing/extreme heat)	-Girls and women between the ages of 11 and 23 were at an increased risk of marrying in the year of, or after, moderate to severe heat waves.<br>-Relative to women of comparable age and education, those who wed during a year with a heat wave married poorer and less educated husbands.<br>-Women who married in years with an extended dry spell married husbands who, on average, had less education and were more supportive of domestic violence.
Tsaneva et al.	The effect of weather variability on child marriage in Bangladesh	2020	Bangladesh (lower middle)	Mixed	Quantitative	Ever-married women aged 25-49 who responded to the Bangladesh DHS in 1999, 2004, 2007, 2011 or 2014 [sample size not found]	University of Delaware's Center for Climatic Research: 0.5 X 0.5° Matsurra & Willmott series	1) Extreme temperatures (increasing/extreme heat)<br>2) Rainfall shocks (negative)	- Higher number of dry months in a given year significantly increases the probability of child marriage.<br>-Precipitation fluctuations only affect rural not urban populations.<br>-The cohorts of women born in the 1950s and 1960s were the most affected one, pointing an increased practice of dowries or different coping mechanisms.

Alston et al.	Are climate challenges reinforcing child and forced marriage and dowry as adaptation strategies in the context of Bangladesh?	2014	Bangladesh (lower middle)	Rural	Quantitative, qualitative	Quantitative: Respondents to a questionnaire across the regions of Gaibandha and Satkhira in 2013 [n=617]<br><br>Qualitative: IDIs [n=23],<br>FGDs [n=29],<br>KIIs with Dhaka civil society representatives from 2011-2012 [n=10]	Context-specific literature	Multiple	-Dowry payments for young girls are cheaper and because of increased food insecurity from climate changes, families pursue child marriage for financial gains.<br>-Climate change challenges lead to school dropouts, forced marriages of very young girls, and experience of violence.
Niaz Asadullah et al.	Child marriage, climate vulnerability and natural disasters in coastal Bangladesh	2020	Bangladesh (lower middle)	Mixed	Quantitative, qualitative	Quantitative: Women from coastal and non-coastal regions who responded to the nationwide survey of women (WiLCAS) in 2014 [n=6272]<br><br>Qualitative:<br>IDIs with women aged 17-45 and married before age 18 [n=75]<br>FGDs with males and females in 2016 [n=48]	Government of the People's Republic of Bangladesh, United Nations Development Programme Bangladesh	Multiple	-Women in coastal districts who experienced more climate vulnerabilities compared to women in non-coastal districts showed a systematically higher early marriage prevalence.<br>-Increased risk of child marriage to cope with shared vulnerability to climate change.
Ahmed et al.	The nexus between extreme weather events, sexual violence, and early marriage: A study of vulnerable populations in Bangladesh	2019	Bangladesh (lower middle)	Rural	Quantitative, qualitative	Quantitative: Male and female household heads aged 34-67 years from Chandi [n=78] and Alipur [n=42] who responded to a questionnaire in 2015<br><br>Qualitative: IDIs with male and female heads of household who had an experience of sexual violence against one of their daughters, they fear sexual violence against their girls, and/or had at least one daughter unmarried or married off before 18 [n=40]	Context specific literature, Upazila Disaster Management Committee	1) Floods<br>2) Cyclones/typhoons	-Early marriage of daughters significantly linked to extreme weather events.<br>-Households hope to minimize related household expenses and to re-allocate resources to pay for damages caused by extreme weather.<br>-Unmarried daughters may be subject to sexual violence during a crisis, especially in temporary shelters, which would harm both the family's and daughter's reputation and prevent future marriage.

**CONTRACEPTION (n=1)**									
Abiona	The impact of unanticipated economic shocks on the demand for contraceptives : Evidence from Uganda	2017	Uganda (low)	Rural	Quantitative	Married women aged between 15-50 who responded to the Ugandan National Panel Survey between 2009-2012 [n=15975]	World Bank, University of Delaware Center for Climatology Research	Rainfall shocks (negative & positive)	-Women (and their husbands) strategically increase the demand for contraceptive measures to not have a baby during periods of shock.<br>-A 0.10 log-points negative rainfall shock induces an increase in the likelihood of the demand for contraceptives by women by approximately 6.7% on average.
**MULTIPLE (n=5)**									
Andriano et al.	The effects of growing-season drought on young women's life course transitions in a sub-Saharan context	2020	Malawi (low)	Rural	Quantitative	Women aged 15-24 who responded to the Malawi DHS in 2000, 2004, 2010, 2015-2016 who had lived in the region since age 9 [n=17033]	ERA-Interim produced by the ECMWF	Drought	-Exposure to growing-season drought significantly increases young women's transitions into first unions, especially when going from no drought exposure to drought exposure for the entire growing season.<br>-Exposure to growing-season drought in adolescence is associated with a significant acceleration in young women's first births conceived within a union.
Rosen et al.	'Burnt by the scorching sun': climate-induced livelihood transformations, reproductive health, and fertility trajectories in drought-affected communities of Zambia	2018	Zambia (low)	Mixed	Qualitative	IDIs [n=20] and FGDs [n=145] with adult women and men in five drought-affected districts<br>KIIs with civic leaders and healthcare providers [n=16] in 2020	Context-specific literature	Drought	-Financial insecurity resulted in early marriage of girls, and exchange or sell of sex to obtain money or supplies<br>-Fertility intentions were changed to due to negative economic situation, family planning service (including contraception use) were highly affected, either due to difficulties to transport to the healthcare facilities or lack of money to continue the contraception use.
Sellers et al.	Climate shocks constrain human fertility in Indonesia	2019	Indonesia (upper middle)	Mixed	Quantitative	Ever-married women of reproductive age (15–49) who responded to the Indonesian Family Life Survey in 1993/94, 1997/98, 2000, 2007/08, or 2014/15 [sample size not found]	Modern-Era Retrospective Analysis for Research and Applications Version 2 from NASA	1) Extreme temperatures (increasing/extreme heat)<br>2) Rainfall shocks (negative)	-Women working on farms were more likely to use family planning and to not give birth when temperatures were unusually higher.<br>-When monsoons onset delayed, the fertility intentions were increased and the family planning was used less,

									especially among those with higher socioeconomic status.
Pradhan et al.	District health systems capacity to maintain healthcare service delivery in Pakistan during floods: A qualitative study	2022	Pakistan (lower middle)	Mixed	Qualitative	KIIs with district stakeholders (District Health Officer/designee, MCH and EPI staff, District Emergency Operation Officer, representative from DDMA and NGOs) and healthcare providers [n=79]<br><br>FGDs with lady health workers [n=56] of five flood-prone districts in Pakistan	Context-specific literature	Floods	-Most of the healthcare facilities had their SRHR services negatively affected by flood.<br>-That resulted in unwanted pregnancies due to unavailability of family planning services; cases of domestic violence, gender-based violence and rape (especially when using toilets at night or collecting supplies, e.g., firewood and water); increased home deliveries, miscarriages and maternal death due to lack of transportation.
Rovin et al.	Linking population, fertility, and family planning with adaptation to climate change: Perspectives from Ethiopia	2013	Ethiopia (low)	Rural	Qualitative	IDIs with community members and leaders [n=24] and policymakers, government representatives, and other key leaders [n=14]<br>FGDs with men and women [n=96] in peri-urban and rural areas in 2008-2009	Vulnerability-Resilience Indicators Model	Multiple	-Affected individuals are considered having less children as a measure to cope with climate change challenges. However, family planning still faces important social barriers.

Table B. List of 99 low- and middle-income countries where included studies were carried out.

African Region (AFR) (n=44)		Region of the Americas (AMR) (n=16)	Eastern Mediterranean Region (EMR) (n=11)	European Region (EUR) (n=11)	South-East Asian Region (SEAR) (n=9)	Western Pacific Region (WPR) (n=8)
Algeria	Lesotho	Belize	Afghanistan	Armenia	Bangladesh	Cambodia
Angola	Liberia	Bolivia	Djibouti	Bosnia Herzegovina	Bhutan	China
Benin	Madagascar	Brazil	Egypt	Georgia	India	Lao PDR
Botswana	Malawi	Colombia	Jordan	Kyrgyzstan	Indonesia	Malaysia
Burkina Faso	Mali	Costa Rica	Morocco	Macedonia, FYR	Myanmar	Mongolia
Burundi	Mauritania	Cuba	Pakistan	Moldova	Nepal	Papua New Guinea
Cabo Verde	Mauritius	Dominican Republic	Somalia	Montenegro	Sri Lanka	Philippines
Cameroon	Mozambique	Ecuador	Sudan	Serbia	Thailand	Vietnam
Central African Republic	Namibia	El Salvador	Syria	Tajikistan	Timor-Leste	
Chad	Niger	Guatemala	Tunisia	Ukraine		
Comoros	Nigeria	Haiti	Yemen	Uzbekistan		
Congo, DR	Rwanda	Honduras				
Congo, Republic	Senegal	Jamaica				

Côte d'Ivoire	Sierra Leone	Nicaragua				
Eritrea	South Africa	Paraguay				
Eswatini	South Sudan	Peru				
Ethiopia	Tanzania					
Gabon	The Gambia					
Ghana	Togo					
Guinea	Uganda					
Guinea-Bissau	Zambia					
Kenya	Zimbabwe					

Table C. List of 75 included articles showing first/corresponding authors' affiliations by institutions/country and authors' affiliations to any LMIC or country of study (in alphabetical order by article title).

Article Title	Name and Institution<br>*(1)= first author*<br>*(C)= corresponding author*	Country of Institution	Income Classification-Country of Institution	Any author(s) affiliated in an LMIC?	Any author(s) affiliated in at least 1 country of study?
A drop of love? Rainfall shocks and spousal abuse: Evidence from rural Peru	Juan-José Díaz (1): Grupo de Análisis para el Desarrollo<br>Victor Saldarriage (C): Paris School of Economics	Peru, France	Middle, High	YES	YES
Age of marriage, weather shocks, and the direction of marriage payments	Lucia Corno (1,C): Cattolica University<br>Nicole Hildebrandt (C): Boston Consulting Group<br>Alessandra Voena (C): The University of Chicago	Italy, USA, USA	High	NO	NO

Analysis of heavy rainfall in Sub-Saharan Africa and HIV transmission risk, HIV prevalence, and sexually transmitted infections, 2005-2017	Jason Nagata (1,C): University of California San Francisco	USA	High	NO	NO
Are climate challenges reinforcing child and forced marriage and dowry as adaptation strategies in the context of Bangladesh?	Margaret Alston (1,C): Department of Social Work & Monash University	Australia	High	NO	NO
Association between natural hazards and postnatal care among the neonates in India: A step towards full coverage using geospatial approach	Papai Barman (1,C): International Institute for Population Sciences	India	Middle	YES	YES
Association between severe drought and HIV prevention and care behaviors in Lesotho: A population-based survey 2016–2017	Andrea Low (1,C): Columbia University	USA	High	YES	YES
Association of daytime-only, nighttime-only, and compound heat waves with preterm birth by urban-rural area and regional socioeconomic status in China	Peiran Chen (1): Ministry of Education<br>Yafei Guo (1) and Qiyong Liu (C): Chinese Center for Disease Control and Prevention<br>Peiran Chen (1) and Juan Liang (C): National Office for Maternal and Child Health Surveillance of China & West China Secondary University Hospital & Sichuan University	China	Middle	YES	YES
Associations between ambient heat exposure early in pregnancy and risk of congenital heart defects: A large population-based study	Xiaolin Yu (1): Shantou University Medical College<br>Pi Guo (C): Shantou University Medical College	China	Middle	YES	YES

Association of maternal exposure to compound hot extreme during pregnancy with preterm birth and the potential biological mechanisms in Guangzhou	Zhou He (1) and Liu Tao (C): Guangdong Provincial Institute of Public Health<br>Zhou He (1): Guangdong Pharmaceutical University<br>Liu Tao (C): Jinan University	China	Middle	YES	YES
"Burnt by the scorching sun": Climate-induced livelihood transformations, reproductive health, and fertility trajectories in drought-affected communities of Zambia	Joseph Rosen (1) and Drosin Mulenga (1) and Lyson Phiri (1) and Nachela Chelwa (1) and Michael Mbizvo (1,C): Population Council of Zambia	Zambia	Middle	YES	YES
Child marriage, climate vulnerability and natural disasters in coastal Bangladesh	M. Niaz Asadullah (1,C): University of Malaya	Malaysia	Middle	YES	NO
Climate anomalies and birth rates in sub-Saharan Africa	Brian Thiede (1,C): Pennsylvania State University	USA	High	NO	NO
Climate shocks constrain human fertility in Indonesia	Samuel Sellers (1,C): University of Washington	USA	High	NO	NO
Climate, birth weight, and agricultural livelihoods in Kenya and Mali	Maryia Bakhtsiyarava (1,C): University of Minnesota	USA	High	NO	NO
Climatic variability and changing reproductive goals in Sub-Saharan Africa	Sarah Eissler (1) Brian C. Thiede (C): Pennsylvania State University	USA	High	NO	NO
Compound vulnerabilities: The intersection of climate variability and HIV/AIDS in northwestern Tanzania	Valerie Githinji (1,C): Wageningen University	Netherlands	High	NO	NO
District health systems capacity to maintain healthcare service delivery in Pakistan during floods: A qualitative study	Nousheen Akber Pradhan (1,C): Aga Khan University	Pakistan	Middle	YES	YES

Drought and disproportionate disease: an investigation of gendered vulnerabilities to HIV/AIDS in less-developed nations	Virginia Kuulei Berndt (1,C): University of Delaware	USA	High	NO	NO
Drought and intimate partner violence towards women in 19 countries in sub-Saharan Africa during 2011-2018: A population-based study	Adrienne Epstein (1,C): University of California, San Francisco	USA	High	NO	NO
Drought and social conflict in rural Zimbabwe: Does the burden fall on women and girls?	Lloyd Chigusiwa (1) and Terrence Kairiza (C): University of Science Education	Zimbabwe	Middle	YES	YES
Drought, HIV testing, and HIV transmission risk behaviors: A Population-based study in 10 high HIV prevalence countries in Sub-Saharan Africa	Adrienne Epstein (1,C): University of California, San Francisco & Liverpool School of Tropical Medicine<br>Jason M. Nagata (1): University of California, San Francisco	USA/England,USA	High	NO	NO
Drying climates and gendered suffering: Links between drought, food insecurity, and women's HIV in less-developed countries	Kelly F. Austin (C,1): Lehigh University	USA	High	NO	NO
Effect of the 2008 cold spell on preterm births in two subtropical cities of Guangdong Province, Southern China	Peng Wang (1) and Zhijiang Liang (1) and Ji-Yuan Zhou (C): Southern Medical University<br>Zhijiang Liang (1): Guangdong Women and Children Hospital	China	Middle	YES	YES
Effect on the health of newborns caused by extreme temperature in Guangzhou	Fanglin Chen (1): Peking University<br>Meiling Liu (1) and Chuanzi Yang (1): Guanzhou Medical University<br>Zongfei Chen (C): Jinan University	China	Middle	YES	YES

Effects of ambient temperature and relative humidity on preterm birth during early pregnancy and before parturition in China from 2010-2018: A population-based large-sample cohort study	Yu Wu (1) and Min Liu (C): Peking University	China	Middle	YES	YES
Effects of climate change and maternal morality: Perspective from case studies in the rural area of Bangladesh	Abu Sayeed Md. Abdullah (1): Center for Injury Prevention and Research Bangladesh<br>Koustuv Dalal (C): Mid Sweden University	Bangladesh, Sweden	Middle, High	YES	YES
Effects of gestational ambient extreme temperature exposures on the risk of preterm birth in China: A sibling-matched study based on a multi-center prospective cohort	Qiong Wang (1) and Haochen Wu (1): Sun Yat-sen University<br>Lina Yin (1) and Aiqun Huang (C): Chinese Center for Disease Control and Prevention<br>Cunrui Huang (C): Tsinghua University	China	Middle	YES	YES
Evidence of interactive effects of late-pregnancy exposure to air pollution and extreme temperature on preterm birth in China: A nationwide study	Xiang Xiao (1) and Meng Gao (C): Hong Kong Baptist University<br>Xiang Xiao (1) and Guang-Hui Dong (C): Sun Yat-sen University<br>Ruixia Liu (1) and Chenghong Yin (C): Capital Medical University<br>Chenghong Yin (C): Capital Medical University<br>Yunjiang Yu (1): Ministry of Environmental Protection	China	Middle	YES	YES

Exploration of the preterm birth risk-related heat event thresholds for pregnant women: A population-based cohort study in China	Meng Ren (1) and Cunrui Huang (C): Sun Yat-sen University<br>Meng Ren (1): Shanghai Meteorological Service, China Meteorological Administration<br>Chunying Zhang (1) and Aiqun Huang (C): Chinese Center for Disease Control and Prevention<br>Cunrui Huang (C): Tsinghua University	China	Middle	YES	YES
Exploring strategies for investigating the mechanisms linking climate and individual-level child health outcomes: An analysis of birth weight in Mali	Kathryn Grace (1,C): University of Minnesota	USA	High	NO	NO
Extreme heat, preterm birth, and stillbirth: A global analysis across 14 lower-middle income countries	Sara McElroy (1,C): University of California San Diego & San Diego State University, & Scripps Institution	USA	High	NO	NO
Extreme temperature exposure and risks of preterm birth subtypes based on a nationwide survey in China	Guoqi Yu (1) and Jun Zhang (C): Shanghai Jiao Tong University School of Medicine<br>Ling Yang (1) and Lichun Fan (C): Hainan Women and Children's Medical Center<br>Ming Liu (1): Tongji University School of Medicine<br>Jun Zhang (C): Shanghai Jiao Tong University School of Public Health	China	Middle	YES	YES
Extreme temperature exposure increases the risk of preterm birth in women with abnormal pre-pregnancy body mass index: A cohort study in a southern province of China	Jialing Qiu (1) and Zhijiang Liang (1) and Xianqiong Luo (C): Guangdong Women and Children Hospital<br>Qingguo Zhao (C): Family Planning Research Institute of Guangdong Province, Guangdong Province Fertility Hospital	China	Middle	YES	YES

Extreme weather and marriage among girls and women in Bangladesh	Amanda R. Carrico (1,C): University of Colorado at Boulder	USA	High	NO	NO
From a drought to HIV: An analysis of the effect of droughts on transactional sex and sexually transmitted infections in Malawi	Carole Treibich (1,C): Université Grenoble Alpes	France	High	NO	NO
Gender-based violence before, during, and after cyclones: Slow violence and layered disasters	Nahid Rezwana (1,C): University of Dhaka	Bangladesh	Middle	YES	YES
Gendered migration responses to drought in Malawi	Luis G. Becerra-Valbuena (1) and Katrin Millock (C): Paris School of Economics Katrin Millock (C): CNRS	France	High	NO	NO
Gendered perspectives on climate change adaptation: A quest for social sustainability in Badlagaree Village, Bangladesh	M. Anwar Hossen (1,C): University of Dhaka	Bangladesh	Middle	YES	YES
Heat wave exposure and semen quality in sperm donation volunteers: A retrospective longitudinal study in south China	Xinyi Deng (1) and Yuewei Lu (C): Sun Yat-sen University Qiling Wang (1) and Xinzong Zhang (C): Guangdong Provincial Fertility Hospital	China	Middle	YES	YES
Immediate needs and concerns among pregnant women during and after Typhoon Haiyan (Yolanda)	Mari Sato (1): Tohoku University	Japan	High	NO	NO
Intersections between climate change and female genital mutilation among the Maasai of Kajiado County, Kenya	Tammary Esho (1,C): Amref Health Africa	Kenya	Middle	YES	YES

Is fertility preference related to perception of the risk of child mortality, changes in landholding, and type of family? A comparative study on populations vulnerable and not vulnerable to extreme weather events in Bangladesh	Shah Md. Atiqul Haq (1,C): Université Catholique de Louvain & Shahjalal University of Science and Technology	Belgium/Bangladesh	High/Middle	YES	YES
Kenyan women bearing the cost of climate change	Elizabeth M. Allen (1,C) and Leso Munala (C): St. Catherine University	USA	High	NO	NO
Linking climate change and health outcomes: Examining the relationship between temperature, precipitation and birth weight in Africa	Kathryn Grace (1,C): University of Utah	USA	High	NO	NO
Linking population, fertility, and family planning with adaptation to climate change: Perspectives from Ethiopia	Kimberly Rovin (1,C): Michigan State University	USA	High	YES	YES
Maternal exposure to extreme high-temperature, particulate air pollution and macrosomia in 14 countries of Africa	Guoao Li (1) and Wenlei Hu (1) and Jiahu Hao (C): Anhui Medical University	China	Middle	YES	NO
Potential impact of ambient temperature on maternal blood pressure and hypertensive disorders of pregnancy: A nationwide multicenter study based on the China birth cohort	Yongqing Sun (1) and Chenghong Yin (C) and Wentao Yue (C) and Qingqing Wu (C) and Ruixia Liu (C): Beijing Maternal and Child Health Care Hospital	China	Middle	YES	NO
Prenatal exposure to long-term heat stress and stillbirth in Ghana: A within-space time-series analysis	Sylvester Dodzi Nyadanu (1,C): Curtin University	Australia	High	YES	YES
Rainfall and birth outcome: Evidence from Kyrgyzstan	My Nguyen (1,C) and Kien Le (1): Ho Chi Minh Open University	Vietnam	Middle	YES	NO
Re-examining the effects of drought on intimate-partner violence	Matthew Cooper (1,C): University of Maryland, Harvard University	USA	High	NO	NO

Rural women's experience of living and giving birth in relief camps in Pakistan	Humaira Maheen (1,C): University of Melbourne	Australia	High	NO	NO
Same environment, stratified impacts? Air pollution, extreme temperatures, and birth weight in South China	Xiaoying Liu (1) and Emily Hannum (C): University of Pennsylvania<br>Qingguao Zhao (C): Research Institute of Guangdong Province	USA, China	High, Middle	YES	YES
Seasonality, climate change, and food security during pregnancy among indigenous and non-indigenous women in rural Uganda: Implications for maternal-infant health	Julia M. Bryson (1,C): University of Guelph, McMaster University	Canada	High	YES	YES
Staying afloat: Community perspectives on health system resilience in the management of pregnancy and childbirth care during floods in Cambodia	Dell D Saulnier (1,C): Karolinska Institutet	Sweden	High	YES	YES
Temperature variability and birthweight: Epidemiological evidence from Africa	Ping Wang (1,C): Yale School of Public Health	USA	High	NO	NO
The effect of weather variability on child marriage in Bangladesh	Magda Tsaneva (1,C): Clark University	USA	High	NO	NO
The effects of growing-season drought on young women's life course transitions in a sub-Saharan context	Liliana Andriano (1,C): University of Oxford	United Kingdom	High	NO	NO
The effects of prenatal exposure to temperature extremes on birth outcomes: The case of China	Xi Chen (1): Yale University<br>Xin Zhang (C): Beijing Normal University	USA, China	High, Middle	YES	YES
The impact of drought on HIV care in rural South Africa: An interrupted time series analysis	Collins C. Iwuji (C,1): Africa Health Research Institute, University of Sussex	South Africa, England	Middle, High	YES	YES

The impact of extreme weather events on fertility preference and gender preference in Bangladesh	Shah Md Atiqul Haq (1,C): Shahjalal University of Science and Technology, Université Catholique de Louvain, National Fund for Scientific Research	Bangladesh, France, Belgium	Middle, High, High	YES	YES
The impact of flooding on people living with HIV: A case study from the Ohangwena Region Namibia	Carmen Anthonj (1,C): University of Bonn	Germany	High	YES	YES
The impact of timing of in utero drought shocks on birth outcomes in rural households: Evidence from Sierra Leone	Olukorede Abiona (1,C): Macquarie University & University of Sydney	Australia	High	NO	NO
The impact of unanticipated economic shocks on the demand for contraceptives: Evidence from Uganda	Olukorede Abiona (C): University of Leicester	United Kingdom	High	NO	NO
The impacts of rainfall shocks on birthweight in Vietnam	Kien Le (1) and My Nguyen (C): Ho Chi Minh City Open University	Vietnam	Middle	YES	YES
The impacts of temperature shocks on birth weight in Vietnam	My Nguyen (1,C) and Kien Le (1): Ho Chi Minh City Open University	Vietnam	Middle	YES	YES
The link between climate change, food security and fertility: The case of Bangladesh	Mengi Chen (1): Copenhagen University<br>Shah Md Atiqul Haq (C): Université Catholique de Louvain, Shahjalal University of Science and Technology	Denmark, Belgium/Bangladesh	High, High/Middle	YES	YES
The mediation of the placenta on the association between maternal ambient temperature exposure and birth weight	Maimaitiminjiang Wulayin (1) and Zhenghong Zhu (1) and Qiong Wang (C): Sun Yat-sen University	China	Middle	YES	YES
The nexus between extreme weather events, sexual violence, and early marriage: A study of vulnerable populations in Bangladesh	Khandaker Jafor Ahmed (1): The University of Adelaide<br>Shah Md Atiqul Haq (C): Université Catholique de Louvain, Shahjalal University of Science and Technology	Australia, Belgium/Bangladesh	High, High/Middle	YES	YES

The perils of climate change: In utero exposure to temperature variability and birth outcomes in the Andean region	Oswaldo Molina (1,C): Universidad del Pacifico	Peru	Middle	YES	YES
The prenatal weekly temperature exposure and neonatal congenital heat disease: A large population-based observational study in China	Weize Xu (1) and Die Li (1) and Qiang Shu (C): National Clinical Research Center for Child Health<br>Zehua Shao (1): Henan Provincial People's Hospital<br>Yanqin You (1): PLA General Hospital	China	Middle	YES	YES
The relationship between extreme ambient temperature and small for gestational age: A cohort study of 1,436,480 singleton term births in China	Xiang Li (1) and Jing Ma (C): Beijing Normal University<br>Guanpeng Dong (C): Henan University	China	Middle	YES	YES
Underlying causes and the impacts of disaster events (floods) on fertility decision in rural Bangladesh	Shah Md. Atiqul Haq (1,C): Catholic University of Louvain, Shahjalal University of Science and Technology	Belgium/Bangladesh	High/Middle	YES	YES
Water scarcity and birth outcomes in the Brazilian semiarid	Rudi Rocha (1,C): Universidade Federal do Rio de Janeiro	Brazil	Middle	YES	YES
Weather shocks and health at birth in Colombia	Mabel Andalón (1,C): Institute for the Study of Labor	Germany	High	NO	NO
Women in natural disasters: A case study from southern coastal region of Bangladesh	Khurshed Alam (1,C): Bangladesh Institute of Social Research	Bangladesh	Middle	YES	YES
