# Supplementary material for: Climate change and sexual and reproductive health and rights research in low-income and middle-income countries: a scoping review
Source: BMJ Public Health. 2024 Aug 24;2(2):e001090. doi: 10.1136/bmjph-2024-001090 (PMC11816306; doi:10.1136/bmjph-2024-001090)
Supplement: online supplemental file 2 [file bmjph-2-2-s002.pdf]

Table showing a summary of characteristics of 75 articles on climate change and sexual and reproductive health and rights, published between 1994 and 2023 included in this review.

Characteristics		No. of articles (%)	References (N=75)
Year published	2023	19 (25·3)	23,38,43,48,49,55-57,61-68,74,80,84
	2022	12 (16·0)	21,35,40,44,46,47,51,72,77,78,85,87
	2021	12 (16·0)	19,20,22,25,31,39,42,45,53,69,71,79
	2020	10 (13·3)	24,26,29,30,32,33,41,60,75,86
	2019	6 (8·0)	27,34,76,81,83,90
	2014	5 (6·7)	18,28,36,58,73
	2018	4 (5·3)	37,50,82,88
	2017	2 (2·7)	52,91
	2016	2 (2·7)	54,59
	2015	2 (2·7)	70,92
	2013	1 (1·3)	89
Regions	African (AFR)	26 (34·7)	19-21,24,31,35,37,39,42,49,55,63,70,72-74,76-78,81,85,86,88-91
	Western Pacific (WPR)	22 (29·3)	40,41,43-45,47,48,50,51,56,57,59-62,64-69,80
	South-East Asia (SEAR)	15 (20·0)	18,25-29,32-34,38,79,82-84,90
	Americas (AMR)	04 (5·3)	23,36,54,58
	All regions	03 (4·0)	22,71,75
	Eastern Mediterranean	02 (2·7)	52,87
	(EMR)	01 (1·3)	46
	European (EUR)	01 (1·3)	30
	AFR/SEAR	01 (1·3)	53
	AFR/AMR/EUR/SEAR/WPR		
Study country income level[¶] by articles	Lower-middle only	27 (36·0)	18,19,21,25-29,31,32,33,34,38,45-47,52,55,59,60,73,76,79,82-84,87
	Upper-middle only	24 (32·0)	23,36,40,41,43,44,48,50,51,56-58,61,62,64-70,74,80,90
	Mixed low income, lower-middle and upper-middle	11 (14·7)	22,24,30,49,53,63,71,72,75,77,85
	Low income only	09 (12·0)	20,35,39,42,78,86,88,89,91
	Mixed low and lower-middle	03 (4·0)	37,81,92
	Mixed lower-middle and	01 (1·3)	54

Characteristics		No. of articles (%)	References (N=75)
	upper-middle		
Study setting	Mixed (rural and urban)	39 (52·0)	19,20,22,26,30,32,33,37,38,42,43,46-49,53-55,57,58,61,63,66,68,69,71,72,75-79,81,84,85,87,88,90,92
	Rural	26 (34·7)	18,21,23,24,25,27,28,29,31,34-36,39,41,45,52,59,60,70,73,74,82,83,86,89,91
	Urban	10 (13·3)	40,44,50,51,56,62,64,65,67,80
Study design*	Quantitative	57 (76·0)	19-24,29,30,33,35-38,40-51,53-58,61-69,71,72,74-81,83-86,90-92
	Qualitative	13 (17·3)	25,26,31,34,39,52,59,60,70,73,87-89
	Mixed quantitative and qualitative	05 (6·7)	18,27,28,32,82
Climate change event**	Drought	18 (~24·0)	20-22,24,25,30,31,35,71-76,78,86,88
	Extreme temperatures	33 (~44·0)	29,33,36,37,40-45,48-51,53-57,61-69,79,80,81,85,90,92
	Floods	10 (~13·3)	19,27,34,38,52,60,70,82,83,87
	Rainfall shocks	14 (~18·7)	23,33,37,46,47,58,73,77,79,81,85,90-92
	Cyclones/typhoons	04 (~5·3)	26,27,59,83
	Multiple (3+) climate factors***	06 (8·0)	18,28,32,39,84,89
Sexual and reproductive health and rights dimension studied	Maternal and newborn health	37 (49·3)	34-69,92
	Gender-based violence	09 (12·0)	18-26
	HIV and other STIS	09 (12·0)	70-78
	Fertility care[1]	07 (9·3)	79-85
	Harmful practices[2]	07 (9·3)	27-33
	Contraception	01 (1·3)	91
	Multiple SRHR domains[3]	05 (6·7)	86-90
Number of countries studied	1	60 (80·0)	18-21,23, 25-29,31-36, 38-48, 50-52, 55-62, 64-70,73,74,76,78-80, 82-84,86-91
	2-3	02 (2·7)	37, 54
	4-20	06 (8·0)	24,49,53,72,81,92
	21-40+	07 (9·3)	22,30,63,71,75,77,85
Country income classification¶ of	None (Both from high)	34 (45·3)	19,20,22,24,28-30,33,35-37,39,42,52,53,55,59,60,63,70-73,75,76,78,81,85,86,89-92

Characteristics		No. of articles (%)	References (N=75)
first and corresponding authors' institution	Upper-middle	20 (26·7)	32,40,43,44,48,49,50,54,56-58,61,62,64-69,80
	Lower-middle	11 (14·7)	18,21,25,26,31,38,45-47,87,88
	Lower-middle/high	06 (8·0)	27,34,79,83,83,84
	Upper-middle/high	04 (5·3)	23,41,51,74
	Low	0 (0·0)	
Any author affiliated with an LMIC institution?	Yes	47 (62·7)	18,21,23,25,26,27,31,32,34,38,39,40,41,43,44,45,46,47,48,49,50 ,51,54,55,56,57, 58,60,61,62,64,65,66,67,68,69,70,74,76,79,80,82,83,84,87,88,89 ,91
	No	28 (37·3)	19,20,22,24,28,29,30,33,35,36,37,42,52,53,59,63,71,72,73,75,77 ,78,81,85,86,90,92
Author (any) affiliated with an institution located in country under study	Yes	43 (57·3)	18,21,23,25-27,31,34,38,39-41,43-45,47,48,50,51,54-58,60,62,64-67,68,69,70,74,76,79,80,82-84, 87-89
	No	32 (42·7)	19,20,22,24,28,29,30,32,33,35,36,37,42,46,49,52,53,59,61,63,71 ,72,73,75,77,78,81,85,86, 90-92
Populations studied	Newborns	28 (37·3)	35-38,40-43,45-51,53,54,57,58,62,63,66-69,79,85,92[††]
	Women of reproductive age	20 (26·6)	19,22,23,24, 25,26,30,31,32,33,64,72,77,78,81,83,84,86,90,91
	Men and/or women(non-pregnant) aged 15+ years	11 (14·7)	21,27, 28,29,73,76,80,82,87-89
	Pregnant women	10 (13·3)	18,34,44,52,55,56,59,60,61,65
	People living with HIV aged 15+ years	04 (5·3)	70,71,74,75
	Migrating and indigenous populations	02 (2·6)	20,39[††]

* Quantitative (employing surveys e.g., Demographic and health surveys, Multiple Indicator Cluster Surveys, birth registries, etc. linked to meteorological or hydrological data); qualitative (employing key informant interviews, in-depth interviews, and focus group discussions in climate change affected/prone areas)

** Climate change figures exceeds totals due to some articles examining two or more climate change event

*** Three or more climate change events (includes drought, extreme temperatures, floods, cyclones)

[1] Fertility care (includes fertility and infertility)

[2] Harmful practices (includes female genital mutilation and forced/child marriage);

[3] Multiple SRHR domains (includes maternal and newborn health, gender-based violence, harmful practices, fertility care)

Characteristics	No. of articles (%)	References (N=75)
[††]Article also includes pregnant and elderly women, and adolescent girls		

[¶] Country income level according to the World Bank country classification for 2023: https://blogs.worldbank.org/opendata/new-world-bank-country-classifications-income-level-2022-2023
